# Prevalence and correlates of substance use and associations with HIV-related outcomes among trans women in the San Francisco Bay Area

**DOI:** 10.1186/s12879-022-07868-4

**Published:** 2022-11-26

**Authors:** Elaine Hsiang, Akua Gyamerah, Glenda Baguso, Jennifer Jain, Willi McFarland, Erin C. Wilson, Glenn-Milo Santos

**Affiliations:** 1 Department of Emergency Medicine, University of California, San Francisco, 505 Parnassus Avenue, M24, Box 203, San Francisco, CA 94143 USA; 2 Department of Community Health Systems, University of California, San Francisco, San Francisco, CA USA; 3 Center for Public Health Research, San Francisco Department of Public Health, University of California, San Francisco, San Francisco, CA USA; 4 Department of Psychiatry, University of California, San Francisco, San Francisco, CA USA; 5 Department of Epidemiology and Biostatistics, University of California, San Francisco, San Francisco, CA USA

**Keywords:** Substance use, Transgender persons, HIV, Social determinants of health, Discrimination

## Abstract

**Background:**

Trans women face tremendous social inequities as well as disproportionate rates of HIV and substance use, yet disaggregated substance use data specific to trans women remain limited.

**Methods:**

We conducted a secondary analysis of baseline data from the Trans*National Study (2016–2017) surveying trans women in the San Francisco Bay Area (n = 629). Multivariable logistic regression was used to analyze socio-demographic and environmental correlates of substance use, and bivariate associations between substance use and HIV-associated outcomes are presented alongside prevalence data.

**Results:**

Over half (52.9%) reported using substances in the prior year, most frequently marijuana, methamphetamine, and crack/cocaine. Those with unmet gender-affirming health care needs, lack of insurance, or a history of experiencing racial violence, transphobic violence, adult housing instability, or extreme poverty had higher odds of substance use. Sex work and condomless anal sex were also independently associated with substance use.

**Conclusions:**

Among this sample of trans women, substance use and substance use concurrent with sex were highly prevalent, and associated with a number of socioeconomic and health care needs. These findings corroborate the need for holistic and intersectional efforts to reduce substance use and HIV risk.

**Supplementary Information:**

The online version contains supplementary material available at 10.1186/s12879-022-07868-4.

## Introduction

Substance use is a key public health issue with wide-ranging health causes and consequences globally and in the United States (US), and is prevalent among trans women [1–3]. Trans women have a higher prevalence of substance use disorder diagnoses when compared to trans men and cisgender adults [4]. Recent 2019–2020 National HIV Behavioral Surveillance data found that 59–66% of trans women in the US report recent non-injection substance use (excluding alcohol), most frequently marijuana, crack/cocaine, and methamphetamine, patterns that are similarly reflected in a previous study based in the San Francisco Bay Area [5, 6].

Among trans women, there are associations between substance use and sexual risk behaviors (e.g., condomless anal sex), sex work, and HIV prevalence [6–8]. Trans women experience high rates of HIV; in San Francisco, HIV prevalence estimates among trans women have remained > 30% since 1997 [9, 10]. Substance use concurrent with sex increases the risk of HIV acquisition through increased chances of condomless anal sex, and data suggest an association specifically between stimulant use (e.g., methamphetamine, crack, and cocaine) and condomless anal sex in this population [11–14].

These health disparities occur in context of pervasive violence and discrimination against trans women. Transphobic violence has been commonly linked to substance use, particularly binge drinking [15–18]. Housing instability has been associated with higher rates of stimulant and marijuana use, as well as lower odds of anti-retroviral therapy use and HIV viral suppression [19–21]. For trans women who do sex work to meet their survival needs, underlying factors that shape sex work risk environments include stigma, interpersonal violence, economic marginalization, and the use of substances to cope with trauma that arises from these experiences [8, 22–24]. Owing to racism, these social and structural barriers to health are compounded for trans women of color, who experience disproportionately high rates of HIV compared to White trans women [10, 15, 25, 26]. Additionally, lack of access to gender affirming health care, including hormone therapy and gender affirming surgeries, is associated with poor mental health and HIV-related outcomes; however, the association between gender affirming care and specific substance use outcomes has not been well studied [17, 27, 28].

While this is a growing body of literature, substance use data for trans women remain severely limited. A recent systematic review identified only 41 studies describing the prevalence and/or correlates of substance use among trans people [17]. This is a dearth of data when compared to the broader body of substance use literature, which has largely focused on cisgender populations, and is compounded by the aggregation of identities and substance use outcomes. Trans women are often grouped with men who have sex with men in HIV studies, and with other sexual and gender minorities in substance use studies, limiting our critical appraisal of specific factors driving substance use among a population uniquely affected by HIV and multiple forms of discrimination [29–31].

Additionally, few studies inclusive of trans women disaggregate types of substance use in presenting substance use patterns or HIV risk data, leaving gaps in our ability to understand the impact of individual substance classes [10, 17, 32, 33]. Effective evidence-based interventions are typically substance-specific and take into account the sociocultural needs of their target populations, necessitating robust data that do not collapse populations of interest or substance categories. As suggested by a 2017 systematic review that identified only two studies describing substance use interventions for trans people, as well as a more recent scoping review demonstrating that substance use interventions for sexual and gender minorities remain heavily focused on sexual minority men, the relationship between the paucity of data to inform the development of substance use interventions for trans individuals cannot be ignored [30, 34].

To address some of these gaps in the literature, we analyzed data from the Trans*National study to identify the prevalence, patterns, and correlates of disaggregated substance use outcomes among trans women in San Francisco. We also examined the relationship between substance use and HIV-related risk behaviors. We included for study substances other than tobacco and alcohol, which are analyzed elsewhere. Together, these efforts may help facilitate the development of substance use interventions that consider the individual and environmental priorities of the community.

## Methods

We performed a secondary analysis of baseline data collected from 2016 to 2017 from the San Francisco site of the Trans*National Study (n = 629), a community-recruited cohort study on HIV incidence and correlates spanning multiple countries (USA, Brazil, Iran, China, Paraguay, and Namibia). We used respondent-driven sampling (RDS), which relies on peer referrals, to maximize diverse recruitment of this often hidden and stigmatized population [35]. Participants were eligible if they were 18 years or older, assigned male sex at birth and did not currently identify as men, and lived in the San Francisco Bay Area. For RDS, we began with 16 seeds, obtained information on participants’ social network size and willingness to refer the study to other trans women in their networks, and tracked referrals among participants, for a final sample size of 629. We purposefully used a large number of starting seeds to approximate population-based sampling. Further detailed recruitment methods are described elsewhere [9]. All participants provided written informed consent to participate in the surveys and most (n = 627) consented to and received HIV testing. The study was approved by the Institutional Review Board at the University of California, San Francisco.

### Measures and outcomes

The baseline questionnaire and codebook are included as Additional file 1. Our primary substance use outcomes were dichotomized: [1] any substance use in the past 12 months, defined as marijuana, methamphetamine, crack/cocaine, downers, prescription painkillers, heroin, club drugs, hallucinogens, synthetic cannabinoids, or poppers, and the subcategories of: (a) marijuana use in the past 12 months, (b) methamphetamine use in the past 12 months, and (c) crack/cocaine use in the past 12 months; [2] any substance use concurrent with sex in the past 12 months, with the same subcategories as above; [3] polysubstance use, defined as two or more substances excluding tobacco and alcohol, in the past 12 months; and [4] any injection drug use in the past 12 months. As a secondary aim, we also assessed substance use as potential predictors of current HIV status and condomless anal sex.

Demographic characteristics included age, gender identity, currently living full-time as gender identity, race/ethnicity, adult housing instability, current living situation, education, sexual orientation, marital status, and whether the participant ever participated in sex work. Ever experiencing adult housing instability was defined as living on the street, in the shelter, or in a single room occupancy hotel at any point age 18 or older. Income was dichotomized based on the US Department of Housing and Urban Development definition of extremely low income limits for San Francisco County (US $27,650 in 2017) [36].

Health characteristics of interest included condomless anal sex in the past 6 months, laboratory-confirmed HIV status, health insurance status, hormone use, and history of gender-affirming surgery. We used questions on whether trans-specific health care and mental health care needs were met to represent access to gender-affirming care in our analyses.

A modified Experiences of Discrimination scale was used to measure discrimination based on race and trans identity in a variety of public settings (e.g., work, school, health care), collapsed into binaries for the purposes of this analysis [37]. Racial and transphobic violence were assessed through specific questions on ever experiencing hate crimes, verbal abuse, and physical assault due to gender and/or racial identities.

### Statistical analysis

Statistical analyses were performed using STATA version 16.1 (College Station, TX). Bivariate logistic regression was performed between the different substance use outcomes and socio-demographic, health, and discrimination measures. Our multivariable logistic regression models followed the recommendations by Hosmer and Lemeshow, whereby covariates with a p-value < 0.25 were included for multivariate analysis, and a stepwise backward elimination approach was utilized to fit the most parsimonious model [38, 39]. Due to collinearity, separate models were fitted for each of our primary substance use outcomes, controlling for age, race, income, and HIV status. The following sociodemographic and health-related covariates were included in the final multivariate models: ever experiencing housing instability as an adult, trans-related discrimination, race-based violence, history of sex work, unmet gender-affirming health care needs, and insurance status. Lastly, we retained bivariate analyses for evaluating substance use and HIV-associated outcomes as the exposures for our various substance use measures were markedly collinear. Missing data were excluded from analysis; all variables analyzed had < 1% missing responses with the exceptions of trans-specific health (3.7% missing) and mental health (13.0% missing) needs. Multivariate results are reported as adjusted odds ratios (AOR) with corresponding 95% confidence intervals (CI) and p-values. Bivariate results are reported as odds ratios (OR) with corresponding 95% CIs and p-values.

## Results

### Descriptive results

Participant demographic characteristics are presented in Table 1. The mean age was 40.5 years (standard deviation = 13, range = 18–75). Within our sample, 32.6% of participants identified as Hispanic or Latinx, 28.9% as White, 17.0% as Black or African American, 3.2% as Asian or Asian American, 1.4% as Native Hawaiian or Pacific Islander, 0.6% as Native American, and 16.2% identified as another or multiple races. At the time of the survey, 95.7% were living full-time as their gender identity (female/woman/transgender, genderqueer/genderfluid, androgynous/ambigender, questioning, or other). Fewer than half (46.9%) of participants rented or owned their home, and 72.5% had experienced some form of adult housing instability. Most (80.3%) participants had completed at least high school or their General Educational Development (GED) tests. The vast majority (77.6%) had an annual income at or below extreme low-income limits as defined above, and 65.1% had a history of sex work.

**Table 1:** Demographic characteristics of trans women at recruitment, San Francisco, 2016–2017 (N = 629)

Characteristic	n	%
Age group in years (Mean = 40.5, Standard Deviation = 13)		
18–24	70	11.1
25–34	168	26.7
35–44	144	22.9
45–54	139	22.1
55–64	89	14.1
65 +	19	3.0
Gender identity		
Female, woman, or transgender	572	90.9
Genderqueer/Genderfluid	23	3.7
Androgynous/Ambigender	3	0.5
Questioning	2	0.3
Other	29	4.6
Currently living full-time as gender identity		
No	25	4.0
Yes	602	95.7
Race/ethnicity		
Hispanic/Latinx	205	32.6
White	182	28.9
Black/African American	107	17.0
Asian/Asian American	20	3.2
Native Hawaiian/Pacific Islander	9	1.4
Native American	4	0.6
Other or Multiple	102	16.2
Ever experienced adult housing instability^a^	456	72.5
Current living situation		
Own or rent	295	46.9
Single room occupancy hotel	122	19.4
Homeless/shelter	106	16.9
Couch surfing or other	69	11.0
Transitional/supportive housing	20	3.2
Residential treatment facility	17	2.7
Education		
Grades 1–8	33	5.2
Grades 9–11	91	14.5
Completed high school or GED^b^	179	28.5
Some college, Associates in Arts degree, or technical degree	208	33.1
Bachelor’s degree	83	13.2
Any postgraduate studies	35	5.6
Annual income		
< $12,060	362	57.6
$12,060–20,000	86	13.7
$20,000–30,000	84	13.4
$30,000–45,000	40	6.4
$45,000–70,000	22	3.5
> $70,000	35	5.6
At or below extremely low-income limit for San Francisco ($27,650 per year)	488	77.6
Sexual orientation		
Straight/Heterosexual	269	42.8
Bisexual	96	15.3
Gay/Lesbian	89	14.1
Queer	73	11.6
Pansexual	52	8.3
Questioning	11	1.7
Other	36	5.7
Marital status		
Never married	436	69.3
Separated or divorced	89	14.1
Married	47	7.5
Living together as married	34	5.4
Widowed	23	3.7
Ever sex work	406	64.5

^a^Defined as ever living on the street, in the shelter, or in a single room occupancy hotel as an adult

^b^General Educational Development tests, an equivalent alternative to the United States high school diploma

Health, health care, and experiences with discrimination and violence are reported in Table 2. The prevalence of HIV in our sample was 29.3%, and 35.1% reported condomless anal sex in the previous 6 months. A minority of participants (5.5%) was uninsured or did not know their insurance status. In terms of gender-affirming care, 90.5% of participants had ever used hormones, and 31.2% had had some form of gender-affirming surgery (e.g. orchiectomy, vaginoplasty, breast implants); 14.3% reported that their health care access did not meet their trans-specific needs, and 12.7% did not feel their mental health care adequately met trans-specific needs. The vast majority (93.2%) of participants had experienced some form of trans-related discrimination, and a majority (77.9% of participants of color) had experienced race-based discrimination. Rates of transphobic and race-based violence were also high, at 88.1% of all participants and 53.9% of participants of color, respectively.

**Table 2:** Health-related characteristics and experiences of discrimination among trans women, San Francisco, 2016–2017 (N = 629)

Characteristic	n	%
HIV status		
Negative	443	70.4
Positive	184	29.3
Condomless anal sex in the past 6 months	221	35.1
Health insurance status		
Uninsured	33	5.2
Insured	591	94.0
Don’t know	2	0.3
Ever hormone use	569	90.5
Had any prior gender-affirming surgery	196	31.2
Health care access met trans-specific needs		
No, but I did not need/access them	14	2.2
No, and I needed them	90	14.3
Yes	502	79.8
Mental health care met trans-specific needs		
No, but I did not need/access them	104	16.5
No, and I needed them	80	12.7
Yes	363	57.7
Ever experienced trans-related discrimination	586	93.2
Ever experienced race-based discrimination	348	55.3^a^
Ever experienced transphobic violence	554	88.1
Ever experienced race-based violence	241	38.3^a^

^a^Percentages are calculated using overall number of participants (n = 629). Of participants of color, 77.9% experienced race-based discrimination and 53.9% experienced race-based violence

More than half (52.9%) of the participants reported substance use in the prior 12 months (Table 3). Marijuana (37.8%), methamphetamine (24.3%), and crack/cocaine (18.1%) were the most frequently used substances. The substances most commonly used with sex were marijuana (24.6%), methamphetamine (18.9%), crack/cocaine (9.5%), and poppers (8.9%). Almost two-thirds (65.8%) of those who used substances reported the use of two or more substances (34.8% of the overall sample). Ever injecting drugs was reported by 27.7%; 9.2% reported injection drug use in the previous 12 months.

**Table 3:** Substance use patterns among trans women, San Francisco, 2016–2017 (N = 629)

Substance Use	n	%
Substances used in the past 12 months		
Marijuana	238	37.8
Methamphetamine	153	24.3
Crack/Cocaine	114	18.1
Club drugs	79	12.6
Hallucinogens	73	11.6
Poppers	67	10.7
Downers	49	7.8
Prescription painkillers	49	7.8
Heroin	36	5.7
Synthetic cannabinoids	15	2.4
Substance use concurrent with sex		
Marijuana	155	24.6
Methamphetamine	119	18.9
Crack/Cocaine	60	9.5
Poppers	56	8.9
Club drugs	44	7.0
Downers	19	3.0
Heroin	17	2.7
Prescription painkillers	14	2.2
Hallucinogens	11	1.7
Synthetic cannabinoids	3	0.5
Any	249	39.6
Number of substances used in the past 12 months		
0	296	47.1
1	114	18.1
2 or more	219	34.8
Ever used injection drugs	174	27.7
Injected drugs in the past 12 months	58	9.2

### Analytic results

**Fig. 1:**
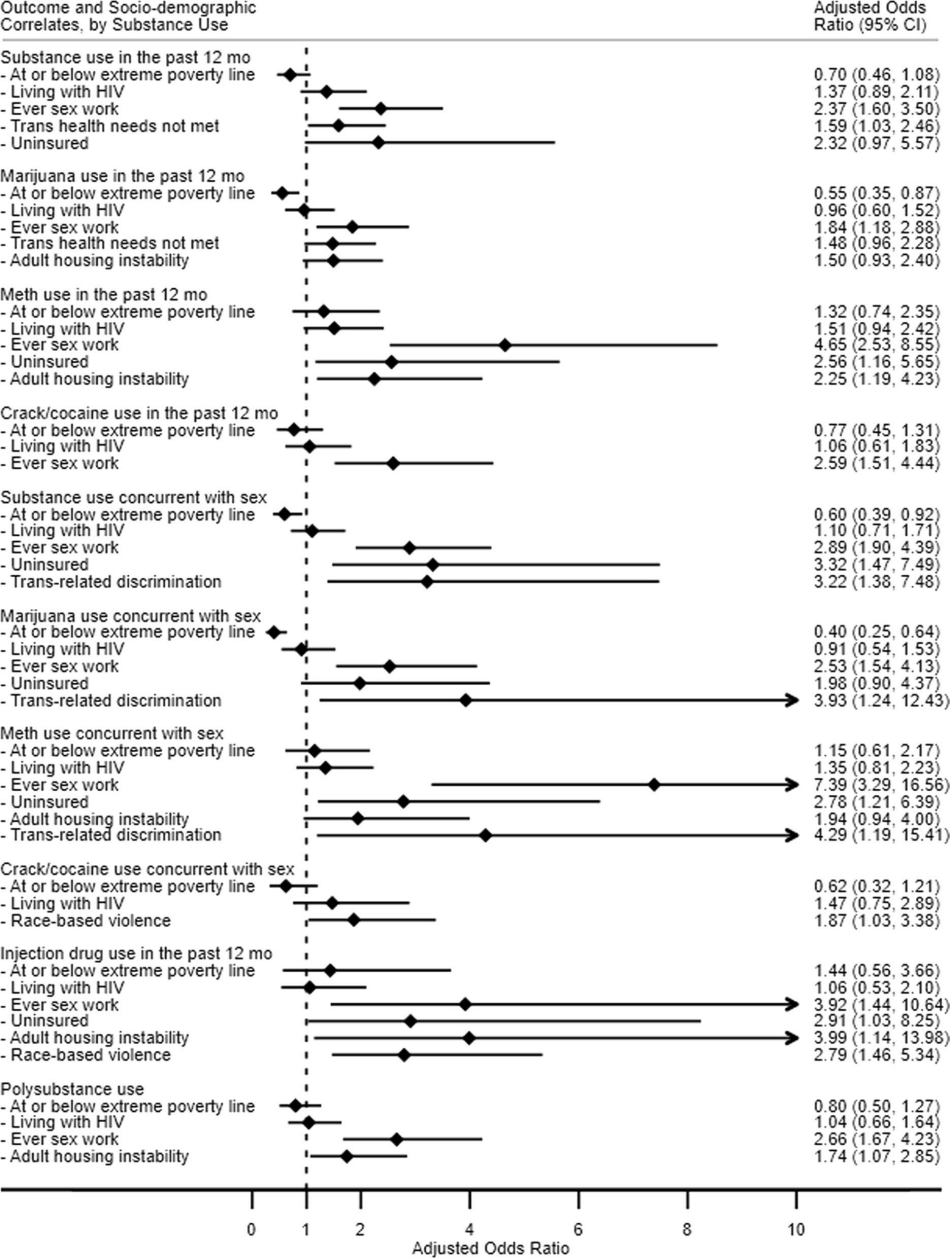
Multivariable logistic regression models for correlates of substance use among trans women, San Francisco, 2016–2017 (N = 629)

Results of the multivariable logistic regressions assessing socio-demographic, health, and discrimination correlates of substance use are shown in Fig. 1. Initial bivariate logistic regressions from which we selected variables for multivariate analysis can be found in Additional file 2: Table S1. Several key factors are associated with different measures of substance use.

Having unmet gender-affirming health care needs was associated with increased adjusted odds of any substance use in the past 12 months (AOR 1.59, 95% CI 1.03–2.46, p = 0.037), compared to those who reported adequate access to gender-affirming health care or those who did not have those needs.

Being uninsured was associated with increased adjusted odds of methamphetamine use in the past 12 months (AOR 2.56, 95% CI 1.16–5.65, p = 0.020), substance use concurrent with sex (AOR 3.32, 95% CI 1.47–7.49, p = 0.004), methamphetamine use concurrent with sex (AOR 2.78, 95% CI 1.21–6.40, p = 0.016), and injection drug use in the past 12 months (AOR 2.91, 95% CI 1.03–8.25, p = 0.044) compared to having insurance.

Ever experiencing housing instability as an adult was associated with higher adjusted odds of methamphetamine use in the past 12 months (AOR 2.25, 95% CI 1.19–4.23, p = 0.012), injection drug use in the past 12 months (AOR 3.99, 95% CI 1.14–13.98, p = 0.031), and polysubstance use (AOR 1.74, 95% CI 1.07–2.85, p = 0.027) compared to those who were stably housed throughout adulthood.

Experiencing trans-related discrimination was associated with increased adjusted odds of any substance use concurrent with sex (AOR 3.22, 95% CI 1.38–7.48, p = 0.007), marijuana use concurrent with sex (AOR 3.93, 95% CI 1.24–12.43, p = 0.020), and methamphetamine use concurrent with sex (AOR 4.29, 95% CI 1.19–15.41, p = 0.026) compared to those who did not report experiencing discrimination based on gender identity or presentation.

Experiencing race-based violence was associated with higher adjusted odds of crack/cocaine use concurrent with sex (AOR 1.87, 95% CI 1.04–3.38, p = 0.038) and injection drug use in the past 12 months (AOR 2.79, 95% CI = 1.46–5.34, p = 0.002), compared to participants who did not report this experience.

**Fig. 2:**
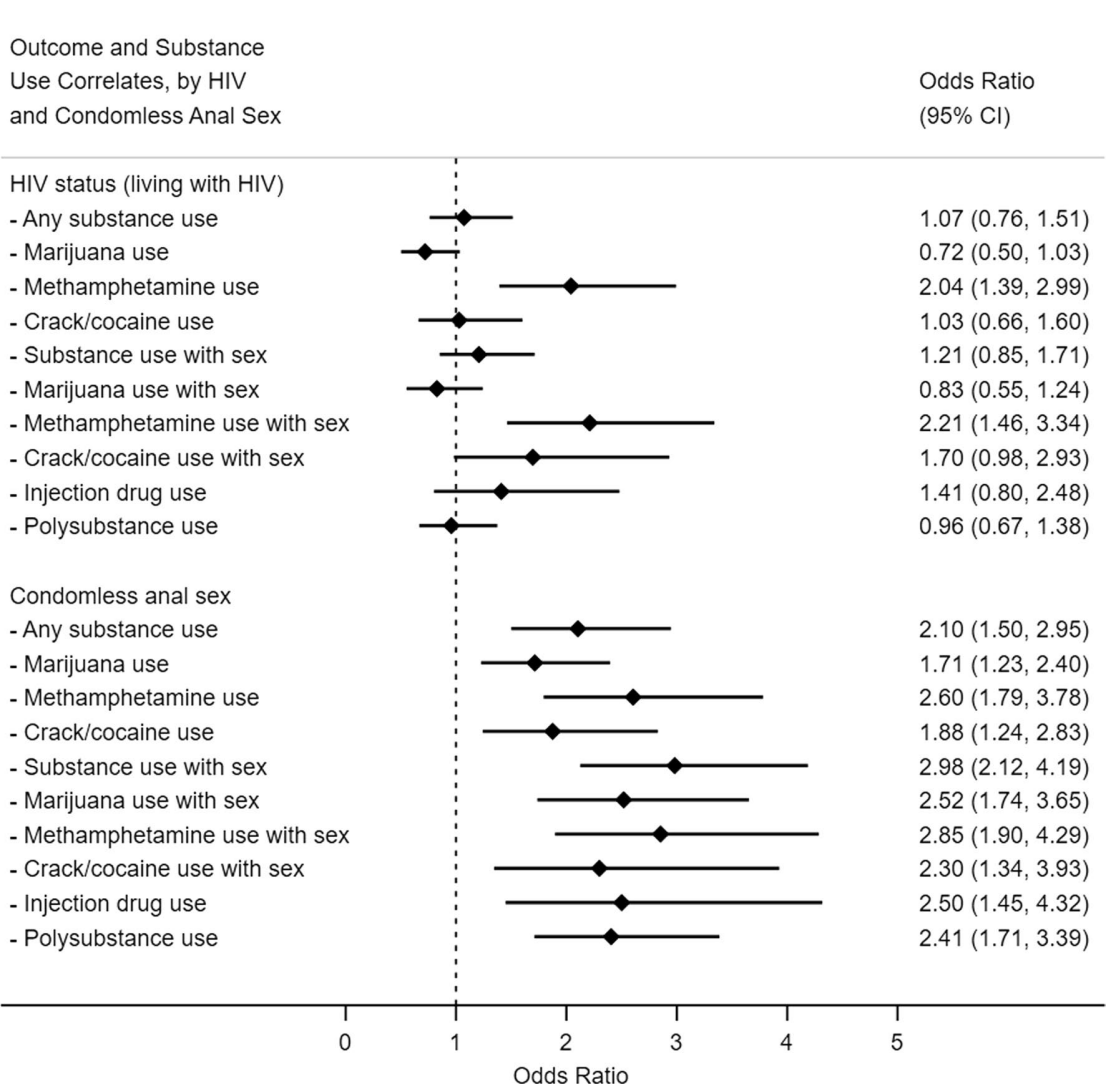
Bivariate logistic regression models for substance use correlates of HIV and condomless anal sex among trans women, San Francisco, 2016–2017 (N = 629)

Figure 2 summarizes bivariate analyses examining associations between substance use and HIV status as well as condomless anal sex. Methamphetamine use (OR 2.04, 95% CI 1.39–2.99, p < 0.001) and methamphetamine use concurrent with sex (OR 2.21, 95% CI 1.46–3.34, p < 0.001) were associated with increased odds of living with HIV. All substance use covariates were significantly associated with higher odds of condomless anal sex.

## Discussion

Our study identified high rates of substance use in a community-based sample of trans women in the San Francisco Bay Area as more than half used substances in the last year, of which nearly two-thirds used at least two substances in the same time period. Nearly one in ten trans women participants used injection drugs in the past year. These numbers for overall substance use and polysubstance use are higher than previous estimates reported for trans women in San Francisco, which may be due to our larger sample size and/or temporal trends in the area [6]. The most commonly used substances, including those used with sex, were marijuana, methamphetamine, and crack/cocaine, which is consistent with recent literature describing substance use among trans women, particularly in the Western US [5, 6, 17]. In considering sociodemographic and health-related factors, we found correlates of various substance use outcomes with adult housing instability, extreme low-income status, race-based violence, trans-related discrimination, HIV status, having unmet gender-affirming health care needs, and lack of health insurance.

Previous data identified transphobic violence, sex work, and sexual minority status as correlates of non-alcohol substance use among trans adults but do not adequately differentiate substance classes or by gender identity [17]. Our multivariate analyses showed that among trans women, trans-related discrimination and sex work were associated specifically with methamphetamine, findings that contribute to emerging data linking experiences of discrimination and violence with stimulant use and bolster the need for viable treatment options specific to methamphetamine [41]. Additionally, our bivariate analyses found increased odds of living with HIV among those who used methamphetamine alone or with sex, further supporting associations between substance use, condomless anal sex, and HIV among trans women [6, 8, 40].

Ever having done sex work was an independent correlate of most of our substance use measures, with particularly high odds ratios for methamphetamine and methamphetamine use with sex. The most proximal use of methamphetamine during sex work is functional, such as by providing energy during long shifts and enhancing sexual performance [42, 43]. While sex work can provide social and economic capital, its associations with criminalization, stigma, and engagement for survival are also linked to substance use [43, 44]. Interventions that address structural factors upstream, such as extreme poverty, incarceration, and barriers to health care, can reduce the need for survival sex work and help mediate the substance use risk environment of trans women who do sex work.

Adult housing instability was independently associated with higher adjusted odds of methamphetamine use, crack/cocaine use with sex, injection drug use, and polysubstance use, building on previous research that associate housing instability with various HIV risk behaviors [21, 41]. A previous study on our cohort of trans women also found an increased risk of housing instability due to multiple layers of discrimination [45]. The demonstrated benefit of housing on substance use and substance use treatment outcomes contributes to the argument that housing interventions for trans women should not require sobriety or pre-existing substance use treatment engagement, especially with the high proportion of housing instability and experiences with discrimination that co-occur in this population [16, 46, 47]. In our results, not having health insurance was also associated with methamphetamine use alone and with sex. An analysis of expansions in insurance coverage under the Affordable Care Act indicated higher substance use treatment utilization for those in the general population who have insurance and want to seek treatment [48]. Another study found that trans women were six times more likely to seek treatment for methamphetamine use than cisgender women, suggesting both higher need and willingness of trans women to engage in substance use treatment [49]. As the cost of out-of-pocket health care can be astronomical, substance use treatments that are covered by insurance may have increased uptake, especially in populations that often experience concurrent health care discrimination, health disparities, as well as lower incomes.

We found independent associations between unmet gender-affirming health care needs and substance use. Prior research has postulated that trans women use substances to cope with health care access disparities and discrimination [16, 50]. However, the impact of accessing gender-affirming care on substance use outcomes has not been well assessed, and in one study was associated with lower odds of non-injection drug use yet higher odds of injection drug use [27]. It is unclear whether the temporality of accessing gender-affirming care affects these outcomes. Additionally, there remains wide variability in the range of services, quality, and utilization patterns of gender-affirming care, as well as pervasive health care stigma and discrimination, even in gender-affirming care settings [51, 52]. This is complicated by the paucity of standardized transgender health curricula in health professions education and the lack of providers who are equipped to care for trans patients [53, 54]. Access to gender-affirming care may not, in itself, reduce substance use or HIV risk, but emerging evidence suggests that substance use interventions that adopt gender-affirming frameworks may be more efficacious when serving trans people [30, 55].

While studies have separately reported high rates of substance use, HIV prevalence, and HIV risk behaviors among trans women of color, few focus on the intersection with racism [8, 15, 25, 56]. Research on sexual minority groups such as men who have sex with men have found associations between racial discrimination and non-alcohol substance use [57]. Our analyses similarly revealed that experiences with race-based violence were associated with higher odds of crack/cocaine use concurrent with sex and injection drug use. The diversity of experiences in the trans community and importance of considering intersectional risk merit further investigation. Future research should measure racism, xenophobia, and experiences with racial discrimination and violence, rather than race and ethnicity alone, in exploring racial disparities.

### Limitations

We used RDS to increase inclusion of a sample that is diverse with regards to certain variables of interest (e.g. race and income); because of this, RDS does not always lead to a population representative sample, and data may not reflect broader communities of trans women. Large and truly representative samples of trans women can be difficult to obtain in this hard-to-reach population, and sampling methodologies such as RDS are subject to the effects of clustering. However, clustering can have beneficial implications for the way interventions or resources are delivered to stigmatized populations, especially as substance use and sexual activity can be highly clustered behaviors reliant on peer networks [58].

Additional limitations include the cross-sectional study design, multiple hypothesis testing, and the possibility of over-selection of variables given the use of backwards elimination in logistic regression. Generalizability is also limited by the fact that substance use patterns can differ by region. Finally, in our bivariate analyses of substance use and HIV-associated outcomes, we could not assess certain key HIV-related risk behaviors (e.g. syringe sharing) due to the scope of data collected in our surveys.

## Conclusions

Our study contributes data on non-alcohol substance use among trans women in light of key health and social priorities of this population. We found that a high proportion of trans women report substance use and substance use concurrent with sex, including methamphetamine and other substances associated with increased HIV risk. Health care and public health interventions should identify and link trans women who use substances with services that account for their sociocultural contexts and socioeconomic needs, including but not limited to housing support, enrollment in health insurance, and access to health care that addresses comprehensive and gender affirming care needs. Interventions must also be informed by the intersectional needs of trans women experiencing transphobia, racism, and other interpersonal and structural forms of violence. As there are few established therapies for some of these highly used substances, the identification of specific correlates of substance use may help inform the design, delivery, and uptake of inclusive interventions for this understudied population.

## Supplementary Information

**Additional file 1. **Questionnaire. Baseline participant questionnaire and codebook.

**Additional file 2: Table 1. **Bivariates. Selected descriptive bivariates for sociodemographic and health-related characteristics.

## Data Availability

The datasets used and/or analysed during the current study are available from the corresponding author on reasonable request.

## Abbreviations

US: United States
RDS: Respondent driven-sampling
GED: General Education Development tests
AOR: Adjusted odds ratio
CI: Confidence interval
OR: Odds ratio
