# Supplementary material for: Prevalence and correlates of substance use and associations with HIV-related outcomes among trans women in the San Francisco Bay Area
Source: BMC Infect Dis. 2022 Nov 26;22:886. doi: 10.1186/s12879-022-07868-4 (PMC9701418; doi:10.1186/s12879-022-07868-4)
Supplement: Supplementary file 1 — Additional file 1. Questionnaire. Baseline participant questionnaire and codebook. [file 12879_2022_7868_MOESM1_ESM.pdf]

Calculated Variable

IDATE Interview Date

IDATE = Today's date

Calculated Variable

START Start Time

START = Current time

Calculated Variable

SIXM Date 6 months ago

SIXM = SHORTDATE (IDATE-183)

Q1. Please enter your interviewer ID

INTID Interviewer ID
4

**0 - 999**	=	range
**9997**	=	Don't Know
**9998**	=	Refuse to Answer
**9999**	=	Not Applicable

Q2. Please Enter Survey ID

SURID Survey ID
5

**0 - 9999**	=	range
**99997**	=	Don't Know
**99998**	=	Refuse to Answer
**99999**	=	Not Applicable

Q3. Please Enter Survey ID Again

SURIDC Survey ID Check
5

**0 - 9999**	=	range
**99997**	=	Don't Know
**99998**	=	Refuse to Answer
**99999**	=	Not Applicable

Q4. Is this person a seed?

I_SEED Seed participant
1

**0**	=	No
**1**	=	Yes
**7**	=	Don't Know
**8**	=	Refuse to Answer
**9**	=	Not Applicable

Q5. Have you already participated in the TEACH 3/Transnational study?

PREVPART Previous Participant
1

0 = No
1 = Yes
7 = Don't Know
8 = Refuse to Answer
9 = Not Applicable

Q6. Did you participate in the TEACH 2 study? (In 2013)

TEACH2PT TEACH2 Participant 1

0 = No
1 = Yes
7 = Don't Know
8 = Refuse to Answer
9 = Not Applicable

Q7. Did you participate in the SHINE study? (The SHINE Study was a study of youth age 16-24 conducted in 2012-2016.)

SHINEPT Shine participant 1

0 = No
1 = Yes
7 = Don't Know
8 = Refuse to Answer
9 = Not Applicable

Q8. What is your date of birth?

DOB Date of birth 8

1/1/1900 - Unlimited = mm/dd/yyyy

Calculated Variable

AGE Age

AGE = AGE(DOB,IDATE)

Q9. So, you are [AGE] years old. Is that correct?

CONFAGE Confirm Age 1

0 = No
1 = Yes
7 = Don't Know
8 = Refuse to Answer
9 = Not Applicable

Q10. What is your gender identity? [Do NOT read choices. Check ONE.]

GENID Gender Identity 2

1 = Male
2 = Female
3 = Transgender female/Transwoman
4 = Androgynous/Ambigender
5 = Genderqueer/Genderfluid

6 = Questioning
7 = Additional sex or gender
97 = Don't Know
98 = Refuse to Answer
99 = Not Applicable

Q11. Please specify other sex or gender:

GENIDSP Other sex or gender
50

0 - 50 = range
97 = Don't Know
98 = Refuse to Answer
99 = Not Applicable

Q12. What sex were you assigned at birth? [Do NOT read choices.]

ASSIGNG Assigned gender
1

1 = Male
2 = Female
3 = Was not assigned a sex
7 = Don't Know
8 = Refuse to Answer
9 = Not Applicable

Q13. Do you consider yourself to be: [Read choices. Check ONE.]

SEXORNT Sexual orientation
2

1 = Straight/Heterosexual
2 = Gay/Lesbian
3 = Bisexual
4 = Pansexual
5 = Queer
6 = Questioning
7 = Other
97 = Don't Know
98 = Refuse to Answer
99 = Not Applicable

Q14. Please specify sexual identity:

SEXORSP Other sexual identity
50

0 - 50 = range
97 = Don't Know
98 = Refuse to Answer
99 = Not Applicable

Q15. Do you consider yourself to be Hispanic or Latina?

HISPLAT Hispanic or Latina
1

0 = No
1 = Yes
7 = Don't Know
8 = Refuse to Answer

9 = Not Applicable

Q16. Which racial group or groups do you consider yourself to be in? You may choose more than one option. [Do NOT read choices. Check ALL that apply]

RACE Race / Ethnicity
2

0 - 7 = range
97 = Don't Know
98 = Refuse to Answer
99 = Not Applicable

RACEA Race / Ethnicity: Asian
1

0 = No
1 = Yes
7 = Don't Know
8 = Refuse to Answer
9 = Not Applicable

RACEB Race / Ethnicity: Black / African American
1

0 = No
1 = Yes
7 = Don't Know
8 = Refuse to Answer
9 = Not Applicable

RACEC Race / Ethnicity: Native American
1

0 = No
1 = Yes
7 = Don't Know
8 = Refuse to Answer
9 = Not Applicable

RACED Race / Ethnicity: Native Hawaiian or Pacific Islander
1

0 = No
1 = Yes
7 = Don't Know
8 = Refuse to Answer
9 = Not Applicable

RACEE Race / Ethnicity: White
1

0 = No
1 = Yes
7 = Don't Know
8 = Refuse to Answer
9 = Not Applicable

RACEF Race / Ethnicity: Latino/a
1

0 = No
1 = Yes

7 = Don't Know
8 = Refuse to Answer
9 = Not Applicable

RACEG Race / Ethnicity: Other
1

0 = No
1 = Yes
7 = Don't Know
8 = Refuse to Answer
9 = Not Applicable

Q17. Please specify race / ethnicity:

RACESP Other race / ethnicity
20

0 - 20 = range
97 = Don't Know
98 = Refuse to Answer
99 = Not Applicable

Q18. What county do you currently live in? [Do NOT read choices. Check ONE.]

COUNTY County you live in
2

1 = San Francisco
2 = Alameda
3 = Contra Costa
4 = Marin
5 = Napa
6 = Santa Clara
7 = Santa Cruz
8 = Sonoma
9 = Solano
10 = San Mateo
11 = Other
97 = Don't Know
98 = Refuse to Answer
99 = Not Applicable

Q19. Please specify other county

COUNTYSP Other county
20

0 - 20 = range
97 = Don't Know
98 = Refuse to Answer
99 = Not Applicable

Q20. Which of the following describes how you know the person who gave you this coupon? [Read choices.]

RECRUIT How do you know the recruiter
1

0 - 5 = range
7 = Don't Know

8 = Refuse to Answer
9 = Not Applicable

RECRUITA How do you know the recruiter: A relative or family member 1

0 = No
1 = Yes
7 = Don't Know
8 = Refuse to Answer
9 = Not Applicable

RECRUITB How do you know the recruiter: A person you have sex with 1

0 = No
1 = Yes
7 = Don't Know
8 = Refuse to Answer
9 = Not Applicable

RECRUITC How do you know the recruiter: A friend 1

0 = No
1 = Yes
7 = Don't Know
8 = Refuse to Answer
9 = Not Applicable

RECRUITD How do you know the recruiter: An acquaintance (that is, a person you know but do not consider a friend) 1

0 = No
1 = Yes
7 = Don't Know
8 = Refuse to Answer
9 = Not Applicable

RECRUITE How do you know the recruiter: A stranger (you dont know the person/just met them) 1

0 = No
1 = Yes
7 = Don't Know
8 = Refuse to Answer
9 = Not Applicable

Q21. I would like to know the main reason why you decided to participate in this study. [Do NOT read choices. Check ONE.]

WHYPART Reason for participation 2

1 = Interested in HIV and sexual health issues
2 = Want to be tested for HIV
3 = Interested in issues relating to transwomen
4 = Wanted to help the community
5 = My friend wanted me to participate
6 = Someone forced me to participate
7 = Incentive/gift for participating

8 = Other
97 = Don't Know
98 = Refuse to Answer
99 = Not Applicable

Q22. Please specify other reason for deciding to participate

WHYPTSP Other reason for participation
50

0 - 50 = range
97 = Don't Know
98 = Refuse to Answer
99 = Not Applicable

Q23. Optional additional screener question (only to be asked if interviewer suspects that participant does not meet eligibility criteria): Could you tell me in a couple sentences about where you are in your transition, or how you feel about transitioning?

SCRNOPT Optional screener describe transition
1

0 = SKIP this question
1 = Non-descriptive
2 = Descriptive
7 = Don't Know
8 = Refuse to Answer
9 = Not Applicable

Q24. [Do NOT Read] Interviewer: Is this person alert and able to complete the health survey?

ALERTABL Alert and able to complete survey
1

0 = No
1 = Yes
7 = Don't Know
8 = Refuse to Answer
9 = Not Applicable

Q25. Interviewer: Specify reason person not able to complete the interview [Check ALL that apply]:

UNABLE Why unable to participate
1

0 - 4 = range
7 = Don't Know
8 = Refuse to Answer
9 = Not Applicable

UNABLEA Why unable to participate: Not alert
1

0 = No
1 = Yes
7 = Don't Know
8 = Refuse to Answer
9 = Not Applicable

UNABLEB Why unable to participate: Not able to complete in English or Spanish
1

0 = No

1 = Yes
7 = Don't Know
8 = Refuse to Answer
9 = Not Applicable

UNABLEC Why unable to participate: Thought to be too young
1

0 = No
1 = Yes
7 = Don't Know
8 = Refuse to Answer
9 = Not Applicable

UNABLED Why unable to participate: Other. Please specify
1

0 = No
1 = Yes
7 = Don't Know
8 = Refuse to Answer
9 = Not Applicable

Q26. [Do NOT Read.] Interviewer: Please specify reason person not able to complete the interview

UNABLSP Specify why unable
100

0 - 100 = range
997 = Don't Know
998 = Refuse to Answer
999 = Not Applicable

Calculated Variable

AGEEL Age Eligibility

AGEEL = IF(AGE>17,1,0)

Calculated Variable

GENIDEL Gender Identity Eligibility

GENIDEL = If((GENID=2 OR GENID=3 OR GENID=4 OR GENID=5 OR GENID=6 OR GENID=7 OR GENID=8),1,0)

Calculated Variable

ASSIGNEL Sex assignment eligibility

ASSIGNEL = if((ASSIGNG=1 OR ASSIGNG=3),1,0)

Calculated Variable

PRVPAREL Previous Participant Eligibility

PRVPAREL = If(PREVPART=0,1,0)

Calculated Variable

DESCELIG Description Eligible

DESCELIG = IF((SCRNOPT=0 OR SCRNOPT=2),1,0)

Calculated Variable

PARTREAS Reason for Participation Eligibility

PARTREAS = If((WHYPART=1 OR WHYPART=2 OR WHYPART=3 OR WHYPART=4 OR WHYPART=5 OR WHYPART=7 OR WHYPART=8),1,0)

Calculated Variable

ELIGIBL Eligibility Criteria

ELIGIBL = If((AGEEL=1 AND ASSIGNEL=1 AND GENIDEL=1 AND ALERTABL=1 AND PRVPAREL=1 AND PARTREAS=1 AND DESCELIG=1),1,0)

Q27. Do you consent to participate in the survey?

CONSSURV Consent for survey
1

0 = No
1 = Yes
7 = Don't Know
8 = Refuse to Answer
9 = Not Applicable

Q28. Do you consent to receiving an HIV test?

CONSTEST Consent for HIV testing
1

0 = No
1 = Yes
7 = Don't Know
8 = Refuse to Answer
9 = Not Applicable

Q29. Do you consent to our collecting a dried blood spot sample for future tests related to HIV?

CONSDBS Consent DBS collection/storage
1

0 = No
1 = Yes
7 = Don't Know
8 = Refuse to Answer
9 = Not Applicable

Q30. Do you consent to receiving a Hepatitis C test?

CONSHCV Consent for HCV testing
1

0 = No
1 = Yes
7 = Don't Know
8 = Refuse to Answer
9 = Not Applicable

Q31. Are you currently in school?

EDUCURR Currently in school
1

0 = No
1 = Yes
7 = Don't Know
8 = Refuse to Answer
9 = Not Applicable

Q32. What is the highest level of education you have completed? [Do NOT read choices.]

EDUCAT Level of education
2

1 = Never attended school
2 = Grades 1 - 8
3 = Grades 9 - 11
4 = Completed high school
5 = Completed GED
6 = AA degree
7 = Technical degree
8 = Some college
9 = Bachelor's degree
10 = Any postgraduate studies
97 = Don't Know
98 = Refuse to Answer
99 = Not Applicable

Q33. Were you born in the US?

BORNUS Born in the US
1

0 = No
1 = Yes
7 = Don't Know
8 = Refuse to Answer
9 = Not Applicable

Q34. How long have you lived in the US? (Number of years)

HOWLNGUS How long lived in US
2

0 - 96 = range
97 = Don't Know
98 = Refuse to Answer
99 = Not Applicable

Q35. Have you ever been an undocumented immigrant?

UNDOCIMM Ever been undocumented immigrant
1

0 = No
1 = Yes
7 = Don't Know
8 = Refuse to Answer
9 = Not Applicable

Q36. What language do you speak at home? [Do NOT read choices.Check ALL that apply]

LA Language spoken at home
1

0 - 5 = range
7 = Don't Know
8 = Refuse to Answer
9 = Not Applicable

LAA Language spoken at home: English
1

0 = No
1 = Yes
7 = Don't Know
8 = Refuse to Answer
9 = Not Applicable

LAB Language spoken at home: Spanish
1

0 = No
1 = Yes
7 = Don't Know
8 = Refuse to Answer
9 = Not Applicable

LAC Language spoken at home: Chinese
1

0 = No
1 = Yes
7 = Don't Know
8 = Refuse to Answer
9 = Not Applicable

LAD Language spoken at home: Tagalog
1

0 = No
1 = Yes
7 = Don't Know
8 = Refuse to Answer
9 = Not Applicable

LAE Language spoken at home: Other
1

0 = No
1 = Yes
7 = Don't Know
8 = Refuse to Answer
9 = Not Applicable

Q37. Please specify your other language.

LAESP Other language
20

0 - 20 = range
97 = Don't Know

98 = Refuse to Answer
99 = Not Applicable

Q38. As a child (before the age of 18), did you ever experience homelessness? By homelessness, I mean sleeping in a shelter, on the street, in an SRO or hotel.

CHILDHOU Childhood housing instability
1

0 = No
1 = Yes
7 = Don't Know
8 = Refuse to Answer
9 = Not Applicable

Q39. As an adult (age 18 or older), have you ever experienced homelessness? By homelessness, I mean sleeping in a shelter, on the street, in an SRO or hotel.

ADULTHOU Adult housing instability
1

0 = No
1 = Yes
7 = Don't Know
8 = Refuse to Answer
9 = Not Applicable

Q40. What best describes your living situation right now? [Do NOT read choices. Check ONE.]

LIVING Living situation
2

1 = Own your own house
2 = Rent a house/apartment/room
3 = Couch surfing with friends or family
4 = Homeless / Shelter
5 = Single Room Occupancy (SRO)
6 = Other
7 = Residential treatment facility
8 = Transitional/supportive housing
97 = Don't Know
98 = Refuse to Answer
99 = Not Applicable

Q41. Please specify your living situation.

LIVINGSP Specify living situation
50

0 - 50 = range
97 = Don't Know
98 = Refuse to Answer
99 = Not Applicable

Q42. Could you stay there for the next 14 days without being asked to leave?

HOUSTAB Housing instability
1

0 = No
1 = Yes
7 = Don't Know
8 = Refuse to Answer

9 = Not Applicable

Q43. How long have you lived in the SF Bay Area? (Number of years)

HOWLNGSF How many years in SFBAY
2

0 - 96 = range
97 = Don't Know
98 = Refuse to Answer
99 = Not Applicable

Q44. Now I have a question about the number of people you know. How many transwomen do you know personally, who live in SF, whom you would be willing to give a recruitment coupon to?

TRNSSF Number of coupon distribution in SF
5

0 - 9999 = range
99997 = Don't Know
99998 = Refuse to Answer
99999 = Not Applicable

Q45. How many other transwomen do you know personally who live in the Greater Bay Area (NOT including those living in SF) whom you be willing to give a recruitment coupon to?

TRNSBAY Number of coupon distribution outside SF
5

0 - 9999 = range
99997 = Don't Know
99998 = Refuse to Answer
99999 = Not Applicable

Calculated Variable

TRNSTOT Number of coupon distribution total

TRNSTOT = TRNSBAY+TRNSSF

Q46. What is your current marital status? [READ Choices.]

MARITAL Marital Status
1

0 = Never married
1 = Separated
2 = Divorced
3 = Widowed
4 = Married
5 = Living together as married
7 = Don't Know
8 = Refuse to Answer
9 = Not Applicable

Q47. In the past month, what were your sources of income/support? [READ all choices. Check ALL that apply]

SUPPORT Financial Support/Income
2

0 - 14 = range

97 = Don't Know
98 = Refuse to Answer
99 = Not Applicable

SUPPORTA Financial Support/Income: A job
1

0 = No
1 = Yes
7 = Don't Know
8 = Refuse to Answer
9 = Not Applicable

SUPPORTB Financial Support/Income: General assistance, food stamps
1

0 = No
1 = Yes
7 = Don't Know
8 = Refuse to Answer
9 = Not Applicable

SUPPORTC Financial Support/Income: Social Security Insurance (SSI)
1

0 = No
1 = Yes
7 = Don't Know
8 = Refuse to Answer
9 = Not Applicable

SUPPORTD Financial Support/Income: Disability
1

0 = No
1 = Yes
7 = Don't Know
8 = Refuse to Answer
9 = Not Applicable

SUPPORTE Financial Support/Income: Unemployment benefits
1

0 = No
1 = Yes
7 = Don't Know
8 = Refuse to Answer
9 = Not Applicable

SUPPORTF Financial Support/Income: Main partner's income
1

0 = No
1 = Yes
7 = Don't Know
8 = Refuse to Answer
9 = Not Applicable

SUPPORTG Financial Support/Income: Friend or family's income
1

0 = No

1 = Yes
7 = Don't Know
8 = Refuse to Answer
9 = Not Applicable

SUPPORTH Financial Support/Income: Drug dealing/selling
1

0 = No
1 = Yes
7 = Don't Know
8 = Refuse to Answer
9 = Not Applicable

SUPPORTI Financial Support/Income: Sex work
1

0 = No
1 = Yes
7 = Don't Know
8 = Refuse to Answer
9 = Not Applicable

SUPPORTJ Financial Support/Income: Alimony/Child support
1

0 = No
1 = Yes
7 = Don't Know
8 = Refuse to Answer
9 = Not Applicable

SUPPORTK Financial Support/Income: Scamming/Stealing
1

0 = No
1 = Yes
7 = Don't Know
8 = Refuse to Answer
9 = Not Applicable

SUPPORTL Financial Support/Income: Student loans
1

0 = No
1 = Yes
7 = Don't Know
8 = Refuse to Answer
9 = Not Applicable

SUPPORTM Financial Support/Income: Other
1

0 = No
1 = Yes
7 = Don't Know
8 = Refuse to Answer
9 = Not Applicable

SUPPORTN Financial Support/Income: Shows/performances

1

0 = No
1 = Yes
7 = Don't Know
8 = Refuse to Answer
9 = Not Applicable

Q48. Please specify your other source of income/support.

SUPPMSP Specify income/support
20

0 - 20 = range
97 = Don't Know
98 = Refuse to Answer
99 = Not Applicable

Q49. What is your average monthly income from ALL SOURCES (before taxes)?

INCOME Income
7

0 - 999999 = range
9999997 = Don't Know
9999998 = Refuse to Answer
9999999 = Not Applicable

Q50. What is your current occupation?

OCCUP Occupation
200

0 - 200 = range
997 = Don't Know
998 = Refuse to Answer
999 = Not Applicable

Q51. In your life, have you ever done sex work? By that I mean exchanging sex for money, goods, or a place to stay.

SEXWORK Sex work history
1

0 = No
1 = Yes
7 = Don't Know
8 = Refuse to Answer
9 = Not Applicable

Q52. How old were you when you first felt you might be something other than your assigned male sex at birth?

GEND1 Age gender awarenes
2

0 - 96 = range
97 = Don't Know
98 = Refuse to Answer
99 = Not Applicable

Q53. Are you currently living and identifying full-time as [Response to Q10]? [Interviewer: if

Other, insert participant's stated gender identity.]

GEND2 Full time trans 1

0 = No
1 = Yes
7 = Don't Know
8 = Refuse to Answer
9 = Not Applicable

Q54. What are the reasons you are not living and identifying full-time as [Response to Q10]? [Interviewer: if Other, insert participant's stated gender identity.] [READ choices.]

WHYNOFT Why not full time trans 2

0 - 7 = range
97 = Don't Know
98 = Refuse to Answer
99 = Not Applicable

WHYNOFTA Why not full time trans: Family disapproves 1

0 = No
1 = Yes
7 = Don't Know
8 = Refuse to Answer
9 = Not Applicable

WHYNOFTB Why not full time trans: Partner disapproves 1

0 = No
1 = Yes
7 = Don't Know
8 = Refuse to Answer
9 = Not Applicable

WHYNOFTC Why not full time trans: Discrimination at work 1

0 = No
1 = Yes
7 = Don't Know
8 = Refuse to Answer
9 = Not Applicable

WHYNOFTD Why not full time trans: Discrimination at home 1

0 = No
1 = Yes
7 = Don't Know
8 = Refuse to Answer
9 = Not Applicable

WHYNOFTE Why not full time trans: Can't afford it 1

0 = No
1 = Yes

7 = Don't Know
8 = Refuse to Answer
9 = Not Applicable

WHYNOFTF Why not full time trans: Not satisfied with presentation
1

0 = No
1 = Yes
7 = Don't Know
8 = Refuse to Answer
9 = Not Applicable

WHYNOFTG Why not full time trans: Other
1

0 = No
1 = Yes
7 = Don't Know
8 = Refuse to Answer
9 = Not Applicable

Q55. Please specify why you are not currently living and identifying full-time as [Response to Q10]. [Interviewer: if Other, insert participant's stated gender identity.]

WNOFTGSP Specify why not living as full time woman
150

0 - 150 = range
997 = Don't Know
998 = Refuse to Answer
999 = Not Applicable

Q56. How old were you when you started living as [Response to Q10]? [Interviewer: if Other, insert participant's stated gender identity.]

GEND3 Age of transition
2

0 - 96 = range
97 = Don't Know
98 = Refuse to Answer
99 = Not Applicable

Q57. Have you ever identified as gay for some period of time before your transition?

GEND4 Gay before trans
1

0 = No
1 = Yes
7 = Don't Know
8 = Refuse to Answer
9 = Not Applicable

Q58. Have you ever taken hormones or drugs to enhance gender presentation?

HORMONES Hormones
1

0 = No
1 = Yes

7 = Don't Know
8 = Refuse to Answer
9 = Not Applicable

Q59. How old were you when you started taking hormones?

AGEHORM Age of hormones
2

0 - 96 = range
97 = Don't Know
98 = Refuse to Answer
99 = Not Applicable

Q60. Are you currently taking hormones?

CURRHORM Currently on hormones
1

0 = No
1 = Yes
7 = Don't Know
8 = Refuse to Answer
9 = Not Applicable

Q61. Do you want to be taking hormones?

DESHORM Want to be on hormones
1

0 = No
1 = Yes
7 = Don't Know
8 = Refuse to Answer
9 = Not Applicable

Q62. Why aren't you currently taking hormones?

HORMBAR Why not taking hormones
2

0 - 10 = range
97 = Don't Know
98 = Refuse to Answer
99 = Not Applicable

HORMBARA Why not taking hormones: Don't have health insurance
1

0 = No
1 = Yes
7 = Don't Know
8 = Refuse to Answer
9 = Not Applicable

HORMBARB Why not taking hormones: Cannot pay for them
1

0 = No
1 = Yes
7 = Don't Know
8 = Refuse to Answer
9 = Not Applicable

HORMBARC Why not taking hormones: Don't know how/where to get them
1

0 = No
1 = Yes
7 = Don't Know
8 = Refuse to Answer
9 = Not Applicable

HORMBARD Why not taking hormones: Health concerns/contraindications
1

0 = No
1 = Yes
7 = Don't Know
8 = Refuse to Answer
9 = Not Applicable

HORMBARE Why not taking hormones: Temporarily stopped while waiting for surgery
1

0 = No
1 = Yes
7 = Don't Know
8 = Refuse to Answer
9 = Not Applicable

HORMBARF Why not taking hormones: Mood swings
1

0 = No
1 = Yes
7 = Don't Know
8 = Refuse to Answer
9 = Not Applicable

HORMBARG Why not taking hormones: Physician doesn't know how to provide them
1

0 = No
1 = Yes
7 = Don't Know
8 = Refuse to Answer
9 = Not Applicable

HORMBARH Why not taking hormones: Physician won't prescribe them
1

0 = No
1 = Yes
7 = Don't Know
8 = Refuse to Answer
9 = Not Applicable

HORMBARI Why not taking hormones: Didn't know about them
1

0 = No
1 = Yes
7 = Don't Know
8 = Refuse to Answer

9 = Not Applicable

HORMBARJ Why not taking hormones: Other
1

0 = No
1 = Yes
7 = Don't Know
8 = Refuse to Answer
9 = Not Applicable

Q63. Please specify what other reason(s) you are not currently taking hormones.

HBARSP Specify other barriers to hormones
100

0 - 100 = range
997 = Don't Know
998 = Refuse to Answer
999 = Not Applicable

Q64. What hormones are you currently taking? [READ all. Check all that apply.]

WHTHORM What hormones
1

0 - 5 = range
7 = Don't Know
8 = Refuse to Answer
9 = Not Applicable

WHTHORMA What hormones: Estrogen
1

0 = No
1 = Yes
7 = Don't Know
8 = Refuse to Answer
9 = Not Applicable

WHTHORMB What hormones: Progesterone
1

0 = No
1 = Yes
7 = Don't Know
8 = Refuse to Answer
9 = Not Applicable

WHTHORMC What hormones: Anti-androgens
1

0 = No
1 = Yes
7 = Don't Know
8 = Refuse to Answer
9 = Not Applicable

WHTHORMD What hormones: Perlutal
1

0 = No

1 = Yes
7 = Don't Know
8 = Refuse to Answer
9 = Not Applicable

WHTHORME What hormones: Other
1

0 = No
1 = Yes
7 = Don't Know
8 = Refuse to Answer
9 = Not Applicable

Q65. Please specify which other hormones you are currently taking.

HORMSP Specify hormones
20

0 - 20 = range
97 = Don't Know
98 = Refuse to Answer
99 = Not Applicable

Q66. Are these hormones prescribed and being taken under the direction of a doctor or nurse?

DIRDOC Hormones taken under medical direction
1

0 = No
1 = Yes
7 = Don't Know
8 = Refuse to Answer
9 = Not Applicable

Q67. Are these hormones covered by your health insurance?

HORMINS Hormones insurance covered
1

0 = No
1 = Yes
7 = Don't Know
8 = Refuse to Answer
9 = Not Applicable

Q68. Have you ever taken non-prescribed hormones?

NONPRESC Ever non-prescribed hormones
1

0 = No
1 = Yes
7 = Don't Know
8 = Refuse to Answer
9 = Not Applicable

Q69. In your life, have you ever taken both prescribed and non-prescribed hormones at the same time?

HORMMIX Hormones prescribed and nonprescribed

1

0 = No
1 = Yes, currently
2 = Yes, in the past
7 = Don't Know
8 = Refuse to Answer
9 = Not Applicable

Q70. Have you ever taken hormones intermittently, and by that I mean started and stopped taking hormones?

HORMINTM Intermittent hormone use
1

0 = No
1 = Yes
7 = Don't Know
8 = Refuse to Answer
9 = Not Applicable

Q71. Have you had any of the following gender-related surgeries or procedures? [READ ALL choices. Check ALL that apply]

TYPSURG Type of surgery
2

0 - 14 = range
97 = Don't Know
98 = Refuse to Answer
99 = Not Applicable

TYPSURGA Type of surgery: Laser hair removal
1

0 = No
1 = Yes
7 = Don't Know
8 = Refuse to Answer
9 = Not Applicable

TYPSURGB Type of surgery: Penectomy (removal of penis)
1

0 = No
1 = Yes
7 = Don't Know
8 = Refuse to Answer
9 = Not Applicable

TYPSURGC Type of surgery: Orchiectomy (removal of the testicles)
1

0 = No
1 = Yes
7 = Don't Know
8 = Refuse to Answer
9 = Not Applicable

TYPSURGD Type of surgery: Vaginoplasty (construction of the vagina)
1

0 = No

1 = Yes
7 = Don't Know
8 = Refuse to Answer
9 = Not Applicable

TYPSURGE Type of surgery: Breast implants
1

0 = No
1 = Yes
7 = Don't Know
8 = Refuse to Answer
9 = Not Applicable

TYPSURGF Type of surgery: Facial Feminization
1

0 = No
1 = Yes
7 = Don't Know
8 = Refuse to Answer
9 = Not Applicable

TYPSURGG Type of surgery: Dermal fillers (Botox, Restylane)
1

0 = No
1 = Yes
7 = Don't Know
8 = Refuse to Answer
9 = Not Applicable

TYPSURGH Type of surgery: Other
1

0 = No
1 = Yes
7 = Don't Know
8 = Refuse to Answer
9 = Not Applicable

TYPSURGI Type of surgery: Electrolysis
1

0 = No
1 = Yes
7 = Don't Know
8 = Refuse to Answer
9 = Not Applicable

TYPSURGJ Type of surgery: Voice surgery
1

0 = No
1 = Yes
7 = Don't Know
8 = Refuse to Answer
9 = Not Applicable

TYPSURGK Type of surgery: Tracheal shave

1

0 = No
1 = Yes
7 = Don't Know
8 = Refuse to Answer
9 = Not Applicable

TYPSURGL Type of surgery: Fat transfer
1

0 = No
1 = Yes
7 = Don't Know
8 = Refuse to Answer
9 = Not Applicable

TYPSURGM Type of surgery: Voice therapy
1

0 = No
1 = Yes
7 = Don't Know
8 = Refuse to Answer
9 = Not Applicable

TYPSURGN Type of surgery: None
1

0 = No
1 = Yes
7 = Don't Know
8 = Refuse to Answer
9 = Not Applicable

Q72. Specify the type of gender confirmation procedure

SURGGSP Specify transgender surgery
20

0 - 20 = range
97 = Don't Know
98 = Refuse to Answer
99 = Not Applicable

Q73. When did you first have laser hair removal conducted?

WNSURGA When laser hair removal
8

Unlimited - Unlimited = mm/dd/yyyy
2097 = Don't Know (Year)
2098 = Refuse to Answer (Year)
2099 = Not Applicable (Year)

Q74. Who conducted your laser hair removal?

WHOSURGA Who conducted laser hair removal
1

1 = Licensed Medical professional
2 = Informal doctor

7 = Don't Know
8 = Refuse to Answer
9 = Not Applicable

Q75. What country was your laser hair removal performed in?

WHRSURGA Where was laser hair removal performed
1

1 = US
2 = Brazil
3 = Mexico
4 = Thailand
5 = Other
7 = Don't Know
8 = Refuse to Answer
9 = Not Applicable

Q76. Was your laser hair removal paid for by your health insurance?

INSSURGA Laser hair removal paid by health insurance
1

0 = No
1 = Yes
7 = Don't Know
8 = Refuse to Answer
9 = Not Applicable

Q77. When was your penectomy conducted?

WNSURGB When penectomy
8

Unlimited - Unlimited = mm/dd/yyyy
2097 = Don't Know (Year)
2098 = Refuse to Answer (Year)
2099 = Not Applicable (Year)

Q78. Who conducted your penectomy?

WHOSURGB Who conducted penectomy
1

1 = Licensed medical professional
2 = Informal doctor
7 = Don't Know
8 = Refuse to Answer
9 = Not Applicable

Q79. What country was your penectomy performed in?

WHRSURGB Where was penectomy performed
1

1 = US
2 = Brazil
3 = Mexico
4 = Thailand
5 = Other
7 = Don't Know
8 = Refuse to Answer
9 = Not Applicable

Q80. Was your penectomy paid for by your health insurance?

INSSURGB Penectomy paid by health insurance 1

0 = No
1 = Yes
7 = Don't Know
8 = Refuse to Answer
9 = Not Applicable

Q81. When was your orchiectomy conducted?

WNSURGC When orchiectomy 8

Unlimited - Unlimited = mm/dd/yyyy
2097 = Don't Know (Year)
2098 = Refuse to Answer (Year)
2099 = Not Applicable (Year)

Q82. Who conducted your orchiectomy?

WHOSURGC Who conducted orchiectomy 1

1 = Licensed medical professional
2 = Informal doctor
7 = Don't Know
8 = Refuse to Answer
9 = Not Applicable

Q83. What country was your orchiectomy performed in?

WHRSURGC Where was orchiectomy performed 1

1 = US
2 = Brazil
3 = Mexico
4 = Thailand
5 = Other
7 = Don't Know
8 = Refuse to Answer
9 = Not Applicable

Q84. Was your orchiectomy paid for by your health insurance?

INSSURGC Orchiectomy paid by health insurance 1

0 = No
1 = Yes
7 = Don't Know
8 = Refuse to Answer
9 = Not Applicable

Q85. When was your SRS conducted?

WNSURGD When SRS 8

Unlimited - Unlimited = mm/dd/yyyy

2097 = Don't Know (Year)
2098 = Refuse to Answer (Year)
2099 = Not Applicable (Year)

Q86. Who conducted your SRS?

WHOSURGD Who conducted SRS 1

1 = Licensed medical professional
2 = Informal doctor
7 = Don't Know
8 = Refuse to Answer
9 = Not Applicable

Q87. What country was your SRS performed in?

WHRSURGD Where was SRS performed 1

1 = US
2 = Brazil
3 = Mexico
4 = Thailand
5 = Other
7 = Don't Know
8 = Refuse to Answer
9 = Not Applicable

Q88. Was your SRS paid for by your health insurance?

INSSURGD SRS paid by health insurance 1

0 = No
1 = Yes
7 = Don't Know
8 = Refuse to Answer
9 = Not Applicable

Q89. When was your breast implant surgery conducted? [First time if more than once]

WNSURGE When breast augmentation 8

Unlimited - Unlimited = mm/dd/yyyy
2097 = Don't Know (Year)
2098 = Refuse to Answer (Year)
2099 = Not Applicable (Year)

Q90. Who conducted your breast implant surgery?

WHOSURGE Who conducted breast augmentation 1

1 = Licensed medical professional
2 = Informal doctor
7 = Don't Know
8 = Refuse to Answer
9 = Not Applicable

Q91. What country was your breast implant surgery performed in?

WHRSURGE Where was breast augmentation performed
1

1 = US
2 = Brazil
3 = Mexico
4 = Thailand
5 = Other
7 = Don't Know
8 = Refuse to Answer
9 = Not Applicable

Q92. Was your breast implant surgery paid for by your health insurance?

INSSURGE breast augmentation paid by health insurance
1

0 = No
1 = Yes
7 = Don't Know
8 = Refuse to Answer
9 = Not Applicable

Q93. When was your FFS conducted?

WNSURGF When FFS
8

Unlimited - Unlimited = mm/dd/yyyy
2097 = Don't Know (Year)
2098 = Refuse to Answer (Year)
2099 = Not Applicable (Year)

Q94. Who conducted your FFS?

WHOSURGF Who conducted FFS
1

1 = Licensed medical professional
2 = Informal doctor
7 = Don't Know
8 = Refuse to Answer
9 = Not Applicable

Q95. What country was your FFS performed in?

WHRSURGF Where was FFS performed
1

1 = US
2 = Brazil
3 = Mexico
4 = Thailand
5 = Other
7 = Don't Know
8 = Refuse to Answer
9 = Not Applicable

Q96. Was your FFS paid for by your health insurance?

INSSURGF FFS paid by health insurance
1

0 = No
1 = Yes
7 = Don't Know
8 = Refuse to Answer
9 = Not Applicable

Q97. When did you get dermal fillers? [First time if more than once]

WNSURGG — When dermal fillers surgery
8

Unlimited - Unlimited = mm/dd/yyyy
2097 = Don't Know (Year)
2098 = Refuse to Answer (Year)
2099 = Not Applicable (Year)

Q98. Who provided your dermal fillers?

WHOSURGG — Who conducted dermal fillers
1

1 = Licensed medical professional
2 = Informal doctor
7 = Don't Know
8 = Refuse to Answer
9 = Not Applicable

Q99. What country did you get dermal fillers in?

WHRSURGG — Where was dermal filler performed
1

1 = US
2 = Brazil
3 = Mexico
4 = Thailand
5 = Other
7 = Don't Know
8 = Refuse to Answer
9 = Not Applicable

Q100. Were your dermal fillers paid for by your health insurance?

INSSURGG — Dermal fillers paid by health insurance
1

0 = No
1 = Yes
7 = Don't Know
8 = Refuse to Answer
9 = Not Applicable

Q101. When was your electrolysis first conducted?

WNSURGI — When electrolysis
8

Unlimited - Unlimited = mm/dd/yyyy
2097 = Don't Know (Year)
2098 = Refuse to Answer (Year)
2099 = Not Applicable (Year)

Q102. Who conducted your electrolysis?

WHOSURGI Who conducted electrolysis
1

1 = Licensed medical professional
2 = Informal doctor
7 = Don't Know
8 = Refuse to Answer
9 = Not Applicable

Q103. What country was your electrolysis performed in?

WHRSURGI Where was electrolysis performed
1

1 = US
2 = Brazil
3 = Mexico
4 = Thailand
5 = Other
7 = Don't Know
8 = Refuse to Answer
9 = Not Applicable

Q104. Was your electrolysis paid for by your health insurance?

INSSURGI Electrolysis paid by health insurance
1

0 = No
1 = Yes
7 = Don't Know
8 = Refuse to Answer
9 = Not Applicable

Q105. When was your voice surgery conducted?

WNSURGJ When voice surgery
8

Unlimited - Unlimited = mm/dd/yyyy
2097 = Don't Know (Year)
2098 = Refuse to Answer (Year)
2099 = Not Applicable (Year)

Q106. Who conducted your voice surgery?

WHOSURGJ Who conducted voice surgery
1

1 = Licensed medical professional
2 = Informal doctor
7 = Don't Know
8 = Refuse to Answer
9 = Not Applicable

Q107. What country was your voice surgery performed in?

WHRSURGJ Where was voice surgery performed
1

1 = US
2 = Brazil

3 = Mexico
4 = Thailand
5 = Other
7 = Don't Know
8 = Refuse to Answer
9 = Not Applicable

Q108. Was your voice surgery paid for by your health insurance?

INSSURGJ Voice surgery paid by health insurance
1

0 = No
1 = Yes
7 = Don't Know
8 = Refuse to Answer
9 = Not Applicable

Q109. When was your tracheal shave conducted?

WNSURGK When tracheal shave
8

Unlimited - Unlimited = mm/dd/yyyy
2097 = Don't Know (Year)
2098 = Refuse to Answer (Year)
2099 = Not Applicable (Year)

Q110. Who conducted your tracheal shave?

WHOSURGK Who conducted tracheal shave
1

1 = Licensed medical professional
2 = Informal doctor
7 = Don't Know
8 = Refuse to Answer
9 = Not Applicable

Q111. What country was your tracheal shave performed in?

WHRSURGK Where was tracheal shave performed
1

1 = US
2 = Brazil
3 = Mexico
4 = Thailand
5 = Other
7 = Don't Know
8 = Refuse to Answer
9 = Not Applicable

Q112. Was your tracheal shave paid for by your health insurance?

INSSURGK Tracheal shave paid by health insurance
1

0 = No
1 = Yes
7 = Don't Know
8 = Refuse to Answer
9 = Not Applicable

Q113. When was your fat transfer conducted?

WNSURGL When fat transfer
8

**Unlimited - Unlimited** =	mm/dd/yyyy
**2097** =	Don't Know (Year)
**2098** =	Refuse to Answer (Year)
**2099** =	Not Applicable (Year)

Q114. Who conducted your fat transfer?

WHOSURGL Who conducted fat transfer
1

**1** =	Licensed medical professional
**2** =	Informal doctor
**7** =	Don't Know
**8** =	Refuse to Answer
**9** =	Not Applicable

Q115. What country was your fat transfer performed in?

WHRSURGL Where was fat transfer performed
1

**1** =	US
**2** =	Brazil
**3** =	Mexico
**4** =	Thailand
**5** =	Other
**7** =	Don't Know
**8** =	Refuse to Answer
**9** =	Not Applicable

Q116. Was your fat transfer paid for by your health insurance?

INSSURGL Fat transfer paid by health insurance
1

**0** =	No
**1** =	Yes
**7** =	Don't Know
**8** =	Refuse to Answer
**9** =	Not Applicable

Q117. When was your voice therapy conducted?

WNSURGM When voice therapy
8

**Unlimited - Unlimited** =	mm/dd/yyyy
**2097** =	Don't Know (Year)
**2098** =	Refuse to Answer (Year)
**2099** =	Not Applicable (Year)

Q118. Who conducted your voice therapy?

WHOSURGM Who conducted voice therapy
1

**1** =	Licensed medical professional
**2** =	Informal doctor

7 = Don't Know
8 = Refuse to Answer
9 = Not Applicable

Q119. What country was your voice therapy performed in?

WHRSURGM Where was voice therapy performed 1

1 = US
2 = Brazil
3 = Mexico
4 = Thailand
5 = Other
7 = Don't Know
8 = Refuse to Answer
9 = Not Applicable

Q120. Was your voice therapy paid for by your health insurance?

INSSURGM Voice therapy paid by health insurance 1

0 = No
1 = Yes
7 = Don't Know
8 = Refuse to Answer
9 = Not Applicable

Q121. When was your [Response to Q72] conducted?

WNSURGH When specified surgery 8

Unlimited - Unlimited = mm/dd/yyyy
2097 = Don't Know (Year)
2098 = Refuse to Answer (Year)
2099 = Not Applicable (Year)

Q122. Who conducted your [Response to Q72] ?

WHOSURGH Who conducted specified surgery 1

1 = Licensed medical professional
2 = Informal doctor
7 = Don't Know
8 = Refuse to Answer
9 = Not Applicable

Q123. What country was your [Response to Q72] performed in?

WHRSURGH Where was specified surgery performed 1

1 = US
2 = Brazil
3 = Mexico
4 = Thailand
5 = Other
7 = Don't Know
8 = Refuse to Answer
9 = Not Applicable

Q124. Was your [Response to Q72] paid for by your health insurance?

INSSURGH Specified surgery paid by health insurance
1

0 = No
1 = Yes
7 = Don't Know
8 = Refuse to Answer
9 = Not Applicable

Q125. Do you plan on having any gender-related surgeries or procedures in the future?

DESURG Desire for surgical procedures
1

0 = No
1 = Yes
7 = Don't Know
8 = Refuse to Answer
9 = Not Applicable

Q126. What are some of the reasons why you don't plan to have gender-related procedures?

NOSURG Reasons not planning on surgeries
2

0 - 11 = range
97 = Don't Know
98 = Refuse to Answer
99 = Not Applicable

NOSURGA Reasons not planning on surgeries: Not interested in them
1

0 = No
1 = Yes
7 = Don't Know
8 = Refuse to Answer
9 = Not Applicable

NOSURGB Reasons not planning on surgeries: Don't have health insurance
1

0 = No
1 = Yes
7 = Don't Know
8 = Refuse to Answer
9 = Not Applicable

NOSURGC Reasons not planning on surgeries: Can't pay for them
1

0 = No
1 = Yes
7 = Don't Know
8 = Refuse to Answer
9 = Not Applicable

NOSURGD Reasons not planning on surgeries: Already had the procedure(s) I wanted
1

0 = No

1 = Yes
7 = Don't Know
8 = Refuse to Answer
9 = Not Applicable

NOSURGE Reasons not planning on surgeries: Partner doesn't want me to
1

0 = No
1 = Yes
7 = Don't Know
8 = Refuse to Answer
9 = Not Applicable

NOSURGF Reasons not planning on surgeries: Reasons related to sex work
1

0 = No
1 = Yes
7 = Don't Know
8 = Refuse to Answer
9 = Not Applicable

NOSURGG Reasons not planning on surgeries: Scared of surgery
1

0 = No
1 = Yes
7 = Don't Know
8 = Refuse to Answer
9 = Not Applicable

NOSURGH Reasons not planning on surgeries: Physician will not allow me to
1

0 = No
1 = Yes
7 = Don't Know
8 = Refuse to Answer
9 = Not Applicable

NOSURGI Reasons not planning on surgeries: Providers deemed me ineligible (housing, substances, etc.)
1

0 = No
1 = Yes
7 = Don't Know
8 = Refuse to Answer
9 = Not Applicable

NOSURGJ Reasons not planning on surgeries: I don't know much about hem
1

0 = No
1 = Yes
7 = Don't Know
8 = Refuse to Answer
9 = Not Applicable

NOSURGK Reasons not planning on surgeries: Other

1

0 = No
1 = Yes
7 = Don't Know
8 = Refuse to Answer
9 = Not Applicable

Q127. Specify what other reason(s) you don't plan to have gender-related procedures.

NOSURGSP Specify other reasons for no procedures 20

0 - 20 = range
97 = Don't Know
98 = Refuse to Answer
99 = Not Applicable

Q128. What gender-related surgeries or procedures do you plan on having in the future? [Do NOT read. Check ALL that apply]

SURGPL Gender related procedures planned 2

0 - 14 = range
97 = Don't Know
98 = Refuse to Answer
99 = Not Applicable

SURGPLA Gender related procedures planned: Laser hair removal 1

0 = No
1 = Yes
7 = Don't Know
8 = Refuse to Answer
9 = Not Applicable

SURGPLB Gender related procedures planned: Penectomy (removal of penis) 1

0 = No
1 = Yes
7 = Don't Know
8 = Refuse to Answer
9 = Not Applicable

SURGPLC Gender related procedures planned: Orchiectomy (removal of the testicles) 1

0 = No
1 = Yes
7 = Don't Know
8 = Refuse to Answer
9 = Not Applicable

SURGPLD Gender related procedures planned: Vaginoplasty (construction of the vagina) 1

0 = No
1 = Yes
7 = Don't Know

8 = Refuse to Answer
9 = Not Applicable

SURGPLE Gender related procedures planned: Breast augmentation
1

0 = No
1 = Yes
7 = Don't Know
8 = Refuse to Answer
9 = Not Applicable

SURGPLF Gender related procedures planned: Facial Feminization
1

0 = No
1 = Yes
7 = Don't Know
8 = Refuse to Answer
9 = Not Applicable

SURGPLG Gender related procedures planned: Dermal fillers (Botox, Restylane)
1

0 = No
1 = Yes
7 = Don't Know
8 = Refuse to Answer
9 = Not Applicable

SURGPLH Gender related procedures planned: Other
1

0 = No
1 = Yes
7 = Don't Know
8 = Refuse to Answer
9 = Not Applicable

SURGPLI Gender related procedures planned: Voice surgery
1

0 = No
1 = Yes
7 = Don't Know
8 = Refuse to Answer
9 = Not Applicable

SURGPLJ Gender related procedures planned: Tracheal shave
1

0 = No
1 = Yes
7 = Don't Know
8 = Refuse to Answer
9 = Not Applicable

SURGPLK Gender related procedures planned: Electrolysis
1

0 = No

1 = Yes
7 = Don't Know
8 = Refuse to Answer
9 = Not Applicable

SURGPLL Gender related procedures planned: Fat transfer
1

0 = No
1 = Yes
7 = Don't Know
8 = Refuse to Answer
9 = Not Applicable

SURGPLM Gender related procedures planned: Voice therapy
1

0 = No
1 = Yes
7 = Don't Know
8 = Refuse to Answer
9 = Not Applicable

SURGPLN Gender related procedures planned: None
1

0 = No
1 = Yes
7 = Don't Know
8 = Refuse to Answer
9 = Not Applicable

Q129. Specify the type of gender confirmation procedure

SURGDSP Specify transgender surgery
20

0 - 20 = range
97 = Don't Know
98 = Refuse to Answer
99 = Not Applicable

Q130. Why haven't you gotten these procedures or sugeries?

SURGBAR Barriers to surgical procedures
2

0 - 10 = range
97 = Don't Know
98 = Refuse to Answer
99 = Not Applicable

SURGBARA Barriers to surgical procedures: Didn't have health insurance
1

0 = No
1 = Yes
7 = Don't Know
8 = Refuse to Answer
9 = Not Applicable

SURGBARB Barriers to surgical procedures: Couldn't pay for them
1

- **0** = No
- **1** = Yes
- **7** = Don't Know
- **8** = Refuse to Answer
- **9** = Not Applicable

SURGBARC Barriers to surgical procedures: Din't know how/where to get them
1

- **0** = No
- **1** = Yes
- **7** = Don't Know
- **8** = Refuse to Answer
- **9** = Not Applicable

SURGBARD Barriers to surgical procedures: Health concerns/contraindications
1

- **0** = No
- **1** = Yes
- **7** = Don't Know
- **8** = Refuse to Answer
- **9** = Not Applicable

SURGBARE Barriers to surgical procedures: On the waiting list
1

- **0** = No
- **1** = Yes
- **7** = Don't Know
- **8** = Refuse to Answer
- **9** = Not Applicable

SURGBARF Barriers to surgical procedures: Mental health concerns/side effects
1

- **0** = No
- **1** = Yes
- **7** = Don't Know
- **8** = Refuse to Answer
- **9** = Not Applicable

SURGBARG Barriers to surgical procedures: Physician didn't know how to provide them
1

- **0** = No
- **1** = Yes
- **7** = Don't Know
- **8** = Refuse to Answer
- **9** = Not Applicable

SURGBARH Barriers to surgical procedures: Physician wouldn't prescribe them
1

- **0** = No
- **1** = Yes
- **7** = Don't Know
- **8** = Refuse to Answer

9 = Not Applicable

SURGBARI Barriers to surgical procedures: Didn't know about them
1

0 = No
1 = Yes
7 = Don't Know
8 = Refuse to Answer
9 = Not Applicable

SURGBARJ Barriers to surgical procedures: Other
1

0 = No
1 = Yes
7 = Don't Know
8 = Refuse to Answer
9 = Not Applicable

Q131. Please specify what other reason(s) you have not had these surgeries or procedures.

SBARSP Specify other barriers to procedures
100

0 - 100 = range
997 = Don't Know
998 = Refuse to Answer
999 = Not Applicable

Q132. Have you ever injected substances other than hormones (e.g. silicone, oil) to enhance your gender presentation?

FILLER Ever used filler
1

0 = No
1 = Yes
7 = Don't Know
8 = Refuse to Answer
9 = Not Applicable

Q133. What substances have you injected? [Do NOT read choices. Check ALL that apply]

FILLTYP Type Filler
2

0 - 8 = range
97 = Don't Know
98 = Refuse to Answer
99 = Not Applicable

FILLTYPA Type Filler: Collagen
1

0 = No
1 = Yes
7 = Don't Know
8 = Refuse to Answer
9 = Not Applicable

FILLTYPB Type Filler: Saline water

1

0 = No
1 = Yes
7 = Don't Know
8 = Refuse to Answer
9 = Not Applicable

FILLTYPC Type Filler: Mesotherapy
1

0 = No
1 = Yes
7 = Don't Know
8 = Refuse to Answer
9 = Not Applicable

FILLTYPD Type Filler: Biopolymer
1

0 = No
1 = Yes
7 = Don't Know
8 = Refuse to Answer
9 = Not Applicable

FILLTYPE Type Filler: Silicone
1

0 = No
1 = Yes
7 = Don't Know
8 = Refuse to Answer
9 = Not Applicable

FILLTYPF Type Filler: Mineral oil
1

0 = No
1 = Yes
7 = Don't Know
8 = Refuse to Answer
9 = Not Applicable

FILLTYPG Type Filler: Other
1

0 = No
1 = Yes
7 = Don't Know
8 = Refuse to Answer
9 = Not Applicable

FILLTYPH Type Filler: PMMA (acrylic)
1

0 = No
1 = Yes
7 = Don't Know
8 = Refuse to Answer
9 = Not Applicable

Q134. Please specify which other filler.

FILLERSP Specify filler 20

0 - 20 = range
97 = Don't Know
98 = Refuse to Answer
99 = Not Applicable

Q135. In what part(s) of your body have you injected these substances? [Read choices. Check all that apply.]

INJBODY Where filler injected 1

0 - 6 = range
7 = Don't Know
8 = Refuse to Answer
9 = Not Applicable

INJBODYA Where filler injected: Breast 1

0 = No
1 = Yes
7 = Don't Know
8 = Refuse to Answer
9 = Not Applicable

INJBODYB Where filler injected: Face 1

0 = No
1 = Yes
7 = Don't Know
8 = Refuse to Answer
9 = Not Applicable

INJBODYC Where filler injected: Buttocks 1

0 = No
1 = Yes
7 = Don't Know
8 = Refuse to Answer
9 = Not Applicable

INJBODYD Where filler injected: Hips 1

0 = No
1 = Yes
7 = Don't Know
8 = Refuse to Answer
9 = Not Applicable

INJBODYE Where filler injected: Thighs 1

0 = No

1 = Yes
7 = Don't Know
8 = Refuse to Answer
9 = Not Applicable

INJBODYF Where filler injected: Other
1

0 = No
1 = Yes
7 = Don't Know
8 = Refuse to Answer
9 = Not Applicable

Q136. Please specify which other body part.

BODYSP Specify injected body part
20

0 - 20 = range
97 = Don't Know
98 = Refuse to Answer
99 = Not Applicable

Q137. How many times have you ever injected these substances in your lifetime?

FILLFREQ Filler frequency
3

0 - 996 = range
997 = Don't Know
998 = Refuse to Answer
999 = Not Applicable

Q138. When did you receive your first injection?

FILL1ST Date of first filler injection
6

Unlimited - Unlimited = mm/yyyy
2097 = Don't Know (Year)
2098 = Refuse to Answer (Year)
2099 = Not Applicable (Year)

Q139. When did you receive your most recent injection?

FILLREC Date of most recent filler injection
6

Unlimited - Unlimited = mm/yyyy
2097 = Don't Know (Year)
2098 = Refuse to Answer (Year)
2099 = Not Applicable (Year)

Q140. Have you received your injections from the same provider every time?

FILLPROV Same provider for fillers
1

0 = No
1 = Yes
7 = Don't Know

8 = Refuse to Answer
9 = Not Applicable

Q141. When did you receive your injection?

FILLWHN Date of filler injection for one time use
6

Unlimited - Unlimited = mm/yyyy
2097 = Don't Know (Year)
2098 = Refuse to Answer (Year)
2099 = Not Applicable (Year)

Q142. Do you see the person who provided your most recent injection as a medical professional?

FILLPROF Pumper viewed as medical professional
1

0 = No
1 = Yes
7 = Don't Know
8 = Refuse to Answer
9 = Not Applicable

Q143. In what city/cities were your injections performed?

FILLWHR Where injections performed
300

0 - 300 = range
997 = Don't Know
998 = Refuse to Answer
999 = Not Applicable

Q144. Have you ever experienced any of the following complications as a result of these injections? [READ choices. Check all that apply.]

INJCOMP Filler complications
2

0 - 20 = range
97 = Don't Know
98 = Refuse to Answer
99 = Not Applicable

INJCOMPA Filler complications: Bleeding
1

0 = No
1 = Yes
7 = Don't Know
8 = Refuse to Answer
9 = Not Applicable

INJCOMPB Filler complications: Bruising
1

0 = No
1 = Yes
7 = Don't Know
8 = Refuse to Answer
9 = Not Applicable

INJCOMPC Filler complications: Pulmonary embolism

1

0 = No
1 = Yes
7 = Don't Know
8 = Refuse to Answer
9 = Not Applicable

INJCOMPD Filler complications: Rash
1

0 = No
1 = Yes
7 = Don't Know
8 = Refuse to Answer
9 = Not Applicable

INJCOMPE Filler complications: Pain in the injected area
1

0 = No
1 = Yes
7 = Don't Know
8 = Refuse to Answer
9 = Not Applicable

INJCOMPF Filler complications: Redness
1

0 = No
1 = Yes
7 = Don't Know
8 = Refuse to Answer
9 = Not Applicable

INJCOMPG Filler complications: Swelling
1

0 = No
1 = Yes
7 = Don't Know
8 = Refuse to Answer
9 = Not Applicable

INJCOMPH Filler complications: Hypersensitivity
1

0 = No
1 = Yes
7 = Don't Know
8 = Refuse to Answer
9 = Not Applicable

INJCOMPI Filler complications: Numbness or loss of feeling
1

0 = No
1 = Yes
7 = Don't Know
8 = Refuse to Answer
9 = Not Applicable

INJCOMPJ Filler complications: Granulomas
1

0 = No
1 = Yes
7 = Don't Know
8 = Refuse to Answer
9 = Not Applicable

INJCOMPK Filler complications: Itchy skin
1

0 = No
1 = Yes
7 = Don't Know
8 = Refuse to Answer
9 = Not Applicable

INJCOMPL Filler complications: Hardened skin (induration)
1

0 = No
1 = Yes
7 = Don't Know
8 = Refuse to Answer
9 = Not Applicable

INJCOMPM Filler complications: Cellulitis
1

0 = No
1 = Yes
7 = Don't Know
8 = Refuse to Answer
9 = Not Applicable

INJCOMPN Filler complications: Ulceration
1

0 = No
1 = Yes
7 = Don't Know
8 = Refuse to Answer
9 = Not Applicable

INJCOMPO Filler complications: Migration or change in shape of area
1

0 = No
1 = Yes
7 = Don't Know
8 = Refuse to Answer
9 = Not Applicable

INJCOMPP Filler complications: Rejection of foreign body
1

0 = No
1 = Yes
7 = Don't Know

8 = Refuse to Answer
9 = Not Applicable

INJCOMPQ Filler complications: Fever
1

0 = No
1 = Yes
7 = Don't Know
8 = Refuse to Answer
9 = Not Applicable

INJCOMPR Filler complications: Weight loss
1

0 = No
1 = Yes
7 = Don't Know
8 = Refuse to Answer
9 = Not Applicable

INJCOMPS Filler complications: Nausea or vomiting
1

0 = No
1 = Yes
7 = Don't Know
8 = Refuse to Answer
9 = Not Applicable

INJCOMPT Filler complications: Other
1

0 = No
1 = Yes
7 = Don't Know
8 = Refuse to Answer
9 = Not Applicable

Q145. Specify other complications.

INJCOMSP Specify other filler complications
50

0 - 50 = range
97 = Don't Know
98 = Refuse to Answer
99 = Not Applicable

Q146. Do you have children?

CHILDREN Have children
1

0 = No
1 = Yes
7 = Don't Know
8 = Refuse to Answer
9 = Not Applicable

Q147. Would you like to have (more) children in the future?

WANTKID Desire children in the future
1

0 = No
1 = Yes
2 = Maybe
7 = Don't Know
8 = Refuse to Answer
9 = Not Applicable

Q148. [SHOW Flash Card A.] How much have considerations around fertility influenced your decisions around hormone use?

HORMFERT Fertility and hormone consideration
1

0 = Not at all
1 = A little bit
2 = Somewhat
3 = A lot
7 = Don't Know
8 = Refuse to Answer
9 = Not Applicable

Q149. [SHOW Flash Card A.] How much have considerations around fertility influenced your decisions about transgender surgery?

SURGFERT Fertility and surgery consideration
1

0 = Not at all
1 = A little bit
2 = Somewhat
3 = A lot
7 = Don't Know
8 = Refuse to Answer
9 = Not Applicable

Q150. [SHOW Flash Card B.] I am able to adapt when changes occur. [READ choices.]

RES1 Resilience 1
1

1 = Rarely true
2 = Sometimes true
3 = Often true
4 = True nearly all the time
7 = Don't Know
8 = Refuse to Answer
9 = Not Applicable

Q151. [SHOW Flash Card B.] I can deal with whatever comes my way. [READ choices.]

RES2 Resilience 2
1

1 = Rarely true
2 = Sometimes true
3 = Often true
4 = True nearly all the time
7 = Don't Know

8 = Refuse to Answer
9 = Not Applicable

Q152. [SHOW Flash Card B.] I try to see the humorous side of things when I am faced with problems. [READ choices.]

RES3 Resilience 3
1

1 = Rarely true
2 = Sometimes true
3 = Often true
4 = True nearly all the time
7 = Don't Know
8 = Refuse to Answer
9 = Not Applicable

Q153. [SHOW Flash Card B.] Having to cope with stress can make me stronger. [READ choices.]

RES4 Resilience 4
1

1 = Rarely true
2 = Sometimes true
3 = Often true
4 = True nearly all the time
7 = Don't Know
8 = Refuse to Answer
9 = Not Applicable

Q154. [SHOW Flash Card B.] I tend to bounce back after illness, injury, or other hardships. [READ choices.]

RES5 Resilience 5
1

1 = Rarely true
2 = Sometimes true
3 = Often true
4 = True nearly all the time
7 = Don't Know
8 = Refuse to Answer
9 = Not Applicable

Q155. [SHOW Flash Card B.] I believe I can achieve my goals, even if there are obstacles. [READ choices.]

RES6 Resilience 6
1

1 = Rarely true
2 = Sometimes true
3 = Often true
4 = True nearly all the time
7 = Don't Know
8 = Refuse to Answer
9 = Not Applicable

Q156. [SHOW Flash Card B.] Under pressure, I stay focused and think clearly. [READ choices.]

RES7 Resilience 7
1

1 = Rarely true
2 = Sometimes true
3 = Often true
4 = True nearly all the time
7 = Don't Know
8 = Refuse to Answer
9 = Not Applicable

Q157. [SHOW Flash Card B.] I am not easily discouraged by failure. [READ choices.]

RES8 Resilience 8
1

1 = Rarely true
2 = Sometimes true
3 = Often true
4 = True nearly all the time
7 = Don't Know
8 = Refuse to Answer
9 = Not Applicable

Q158. [SHOW Flash Card B.] I think of myself as a strong person when dealing with life's challenges and difficulties. [READ choices.]

RES9 Resilience 9
1

1 = Rarely true
2 = Sometimes true
3 = Often true
4 = True nearly all the time
7 = Don't Know
8 = Refuse to Answer
9 = Not Applicable

Q159. [SHOW Flash Card B.] I am able to handle unpleasant or painful feelings like sadness, fear, and anger. [READ choices.]

RES10 Resilience 10
1

1 = Rarely true
2 = Sometimes true
3 = Often true
4 = True nearly all the time
7 = Don't Know
8 = Refuse to Answer
9 = Not Applicable

Q160. From whom do you think you get the most social support? [READ choices. Check ONE.]

SS1 Social support 1
2

1 = Parent(s) (biologic, adoptive, or foster)
2 = Sibling(s) (biologic, adoptive, or foster)
3 = Aunt/uncle/grandparent(s) (biologic, adoptive, or foster)
4 = Chosen family
5 = Support group
6 = Friend(s)
7 = Mentor

8 = Partner
9 = Other
10 = No one/myself
97 = Don't Know
98 = Refuse to Answer
99 = Not Applicable

Q161. Specify other supportive person:

SSSP Specify support person
50

0 - 50 = range
97 = Don't Know
98 = Refuse to Answer
99 = Not Applicable

Q162. [SHOW Flash Card C.] My [Response to Q160] cares about my feelings. [READ choices.]

SS2 Social support 2
1

1 = Strongly disagree
2 = Somewhat disagree
3 = Somewhat agree
4 = Strongly agree
7 = Don't Know
8 = Refuse to Answer
9 = Not Applicable

Q163. [SHOW Flash Card C.] I can share joys and sorrows with my [Response to Q160]. [READ choices.]

SS3 Social support 3
1

1 = Strongly disagree
2 = Somewhat disagree
3 = Somewhat agree
4 = Strongly agree
7 = Don't Know
8 = Refuse to Answer
9 = Not Applicable

Q164. [SHOW Flash Card C.] My [Response to Q160] is a real source of comfort to me. [READ choices.]

SS4 Social support 4
1

1 = Strongly disagree
2 = Somewhat disagree
3 = Somewhat agree
4 = Strongly agree
7 = Don't Know
8 = Refuse to Answer
9 = Not Applicable

Q165. [SHOW Flash Card C.] My [Response to Q160] is around when I am in need. [READ choices.]

SS5 Social support 5
1

1 = Strongly disagree
2 = Somewhat disagree
3 = Somewhat agree
4 = Strongly agree
7 = Don't Know
8 = Refuse to Answer
9 = Not Applicable

Q166. [SHOW Flash Card C.] My family really tries to help me. [READ choices.]

SS6 Social support 6
1

1 = Strongly disagree
2 = Somewhat disagree
3 = Somewhat agree
4 = Strongly agree
7 = Don't Know
8 = Refuse to Answer
9 = Not Applicable

Q167. [SHOW Flash Card C.] I can talk about my problems with my family. [READ choices.]

SS7 Social support 7
1

1 = Strongly disagree
2 = Somewhat disagree
3 = Somewhat agree
4 = Strongly agree
7 = Don't Know
8 = Refuse to Answer
9 = Not Applicable

Q168. [SHOW Flash Card C.] My family is willing to help me make decisions. [READ choices.]

SS8 Social support 8
1

1 = Strongly disagree
2 = Somewhat disagree
3 = Somewhat agree
4 = Strongly agree
7 = Don't Know
8 = Refuse to Answer
9 = Not Applicable

Q169. [SHOW Flash Card C.] I get the emotional help and support I need from my family. [READ choices.]

SS9 Social support 9
1

1 = Strongly disagree
2 = Somewhat disagree
3 = Somewhat agree
4 = Strongly agree
7 = Don't Know
8 = Refuse to Answer
9 = Not Applicable

Q170. [SHOW Flash Card C.] My friends really try to help me. [READ choices.]

SS10 Social support 10
1

1 = Strongly disagree
2 = Somewhat disagree
3 = Somewhat agree
4 = Strongly agree
7 = Don't Know
8 = Refuse to Answer
9 = Not Applicable

Q171. [SHOW Flash Card C.] I can talk about my problems with my friends. [READ choices.]

SS11 Social support 11
1

1 = Strongly disagree
2 = Somewhat disagree
3 = Somewhat agree
4 = Strongly agree
7 = Don't Know
8 = Refuse to Answer
9 = Not Applicable

Q172. [SHOW Flash Card C.] I have friends with whom I can share my joys and sorrows. [READ choices.]

SS12 Social support 12
1

1 = Strongly disagree
2 = Somewhat disagree
3 = Somewhat agree
4 = Strongly agree
7 = Don't Know
8 = Refuse to Answer
9 = Not Applicable

Q173. [SHOW Flash Card C.] I can count on my friends when things go wrong. [READ choices.]

SS13 Social support 13
1

1 = Strongly disagree
2 = Somewhat disagree
3 = Somewhat agree
4 = Strongly agree
7 = Don't Know
8 = Refuse to Answer
9 = Not Applicable

Q174. Have you legally changed your name?

NAMECH Changed name
1

0 = No
1 = Yes
7 = Don't Know
8 = Refuse to Answer
9 = Not Applicable

Q175. Do you plan to change your name?

PLANAME Plan to change name 1

0 = No
1 = Yes
7 = Don't Know
8 = Refuse to Answer
9 = Not Applicable

Q176. How old were you when you legally changed your name?

AGENMCH Age name change 2

0 - 96 = range
97 = Don't Know
98 = Refuse to Answer
99 = Not Applicable

Q177. Have you legally changed your gender marker?

GENMRKCH Changed gender marker 1

0 = No
1 = Yes
7 = Don't Know
8 = Refuse to Answer
9 = Not Applicable

Q178. Do you plan to change your gender marker?

PLANGMK Plan to change gender marker 1

0 = No
1 = Yes
7 = Don't Know
8 = Refuse to Answer
9 = Not Applicable

Q179. How old were you when you first changed your gender marker?

AGEGMRK Age gender marker change 2

0 - 96 = range
97 = Don't Know
98 = Refuse to Answer
99 = Not Applicable

Q180. On what documents have you changed your gender marker? [READ choices. Check ALL that apply]

GENMRK Documents with new gender marker 2

0 - 7 = range
97 = Don't Know
98 = Refuse to Answer
99 = Not Applicable

GENMRKA Documents with new gender marker: State driver's license or ID
1

0 = No
1 = Yes
7 = Don't Know
8 = Refuse to Answer
9 = Not Applicable

GENMRKB Documents with new gender marker: Birth certificate
1

0 = No
1 = Yes
7 = Don't Know
8 = Refuse to Answer
9 = Not Applicable

GENMRKC Documents with new gender marker: Passport
1

0 = No
1 = Yes
7 = Don't Know
8 = Refuse to Answer
9 = Not Applicable

GENMRKD Documents with new gender marker: Social security Administration records
1

0 = No
1 = Yes
7 = Don't Know
8 = Refuse to Answer
9 = Not Applicable

GENMRKE Documents with new gender marker: Financial records
1

0 = No
1 = Yes
7 = Don't Know
8 = Refuse to Answer
9 = Not Applicable

GENMRKF Documents with new gender marker: School records
1

0 = No
1 = Yes
7 = Don't Know
8 = Refuse to Answer
9 = Not Applicable

GENMRKG Documents with new gender marker: Other
1

0 = No
1 = Yes
7 = Don't Know
8 = Refuse to Answer

9 = Not Applicable

Q181. Please specify other documents

GENMRKSP Specify other documents 50

- **0 - 50** = range
- **97** = Don't Know
- **98** = Refuse to Answer
- **99** = Not Applicable

Q182. [SHOW Flash Card A.] How well do you feel you "pass" in society as cis-gender female?

PASSFEMA Pass as female self assessment 1

- **0** = Not at all
- **1** = A little bit
- **2** = Somewhat
- **3** = A lot
- **7** = Don't Know
- **8** = Refuse to Answer
- **9** = Not Applicable

Q183. [SHOW Flash Card A.] How important is passing to you?

PASSSLF Importance of passing female 1

- **0** = Not at all
- **1** = A little bit
- **2** = Somewhat
- **3** = A lot
- **7** = Don't Know
- **8** = Refuse to Answer
- **9** = Not Applicable

Q184. [SHOW Flash Card A.] How important is passing to your self-esteem?

PASSESTE Importance of passing female to self esteem 1

- **0** = Not at all
- **1** = A little bit
- **2** = Somewhat
- **3** = A lot
- **7** = Don't Know
- **8** = Refuse to Answer
- **9** = Not Applicable

Q185. [SHOW Flash Card A.] How important is passing to your safety?

PASSSAFE Importance to pass female to safety 1

- **0** = Not at all
- **1** = A little bit
- **2** = Somewhat
- **3** = A lot
- **7** = Don't Know
- **8** = Refuse to Answer

9 = Not Applicable

Q186. [SHOW Flash Card A.] How often do you feel you are being clocked (or having your gender identity questioned)?

CLOCKED Feel clocked self assessment 1

0 = Not at all
1 = A little bit
2 = Somewhat
3 = A lot
7 = Don't Know
8 = Refuse to Answer
9 = Not Applicable

Q187. [SHOW Flash Card A.] How important is being perceived as attractive by others to your self-esteem?

PERCATTR Importance of perceived attractiveness 1

0 = Not at all
1 = A little bit
2 = Somewhat
3 = A lot
7 = Don't Know
8 = Refuse to Answer
9 = Not Applicable

Q188. [SHOW Flash Card A.] Does receiving sexual advances or attention validate/confirm your gender?

ATTNCONF Validation from sexual advances 1

0 = Not at all
1 = A little bit
2 = Somewhat
3 = A lot
7 = Don't Know
8 = Refuse to Answer
9 = Not Applicable

Q189. Have you had a serious relationship since you began identifying as trans, meaning a relationship with someone lasting more than 3 months?

RELTRANS Serious relationship since trans 1

0 = No
1 = Yes
7 = Don't Know
8 = Refuse to Answer
9 = Not Applicable

Q190. [SHOW Flash Card A.] Does being in a relationship validate/confirm your gender identity?

RELVALID Validation from relationship 1

0 = Not at all
1 = A little bit

2 = Somewhat
3 = A lot
7 = Don't Know
8 = Refuse to Answer
9 = Not Applicable

Q191. If you feel that you've been treated unfairly, do you usually: [READ choices. Check ONE.]

EOD1 EOD1
1

1 = Accept it as a fact of life
2 = Try to do something about it
7 = Don't Know
8 = Refuse to Answer
9 = Not Applicable

Q192. If you have been treated unfairly, do you usually: [READ choices. Check ONE.]

EOD2 EOD2
1

1 = Talk to other people about it
2 = Keep it to yourself
7 = Don't Know
8 = Refuse to Answer
9 = Not Applicable

Q193. Have you ever experienced discrimination, been prevented from doing something, or been hassled or made to feel inferior because of your gender identity or presentation, or race, ethnicity or color:

At school?

EOD3 EOD3
1

0 = No
1 = Yes
7 = Don't Know
8 = Refuse to Answer
9 = Not Applicable

Q194. Was this related to:

EOD3WHY EOD3 why
1

1 = Gender identity or presentation
2 = Race, ethnicity, or color
3 = BOTH gender identity and race/ethnicity
7 = Don't Know
8 = Refuse to Answer
9 = Not Applicable

Q195. Did experiencing discrimination related to your gender identity or presentation ever lead you to stop attending school?

EOD3DROP EOD3 lead to school dropout
1

0 = No

1 = Yes
7 = Don't Know
8 = Refuse to Answer
9 = Not Applicable

Q196. Have you ever experienced discrimination, been prevented from doing something, or been hassled or made to feel inferior because of your gender identity or presentation, or race, ethnicity or color:

<u>Getting hired or getting a job</u>?

EOD4 EOD4
1

0 = No
1 = Yes
7 = Don't Know
8 = Refuse to Answer
9 = Not Applicable

Q197. Was this related to:

EOD4WHY EOD4 why
1

1 = Gender identity or presentation
2 = Race, ethnicity, or color
3 = BOTH gender identity and race/ethnicity
7 = Don't Know
8 = Refuse to Answer
9 = Not Applicable

Q198. Have you ever experienced discrimination, been prevented from doing something, or been hassled or made to feel inferior because of your gender identity or presentation, or race, ethnicity or color:

<u>At work</u>?

EOD5 EOD5
1

0 = No
1 = Yes
7 = Don't Know
8 = Refuse to Answer
9 = Not Applicable

Q199. Was this related to:

EOD5WHY EOD5 why
1

1 = Gender identity or presentation
2 = Race, ethnicity, or color
3 = BOTH gender identity and race/ethnicity
7 = Don't Know
8 = Refuse to Answer
9 = Not Applicable

Q200. Have you ever experienced discrimination, been prevented from doing something, or been

hassled or made to feel inferior because of your gender identity or presentation, or race, ethnicity or color:

Getting housing?

EOD6 EOD6
1

0 = No
1 = Yes
7 = Don't Know
8 = Refuse to Answer
9 = Not Applicable

Q201. Was this related to:

EOD6WHY EOD6 why
1

1 = Gender identity or presentation
2 = Race, ethnicity, or color
3 = BOTH gender identity and race/ethnicity
7 = Don't Know
8 = Refuse to Answer
9 = Not Applicable

Q202. Have you ever experienced discrimination, been prevented from doing something, or been hassled or made to feel inferior because of your gender identity or presentation, or race, ethnicity or color:

While staying in a shelter, SRO, or residential treatment?

EOD7 EOD7
1

0 = No
1 = Yes
7 = Don't Know
8 = Refuse to Answer
9 = Not Applicable

Q203. Was this related to:

EOD7WHY EOD7 why
1

1 = Gender identity or presentation
2 = Race, ethnicity, or color
3 = BOTH gender identity and race/ethnicity
7 = Don't Know
8 = Refuse to Answer
9 = Not Applicable

Q204. Have you ever experienced discrimination, been prevented from doing something, or been hassled or made to feel inferior because of your gender identity or presentation, or race, ethnicity or color:

Getting medical care?

EOD8 EOD8

1

0 = No
1 = Yes
7 = Don't Know
8 = Refuse to Answer
9 = Not Applicable

Q205. Was this related to:

EOD8WHY EOD8 why
1

1 = Gender identity or presentation
2 = Race, ethnicity, or color
3 = BOTH gender identity and race/ethnicity
7 = Don't Know
8 = Refuse to Answer
9 = Not Applicable

Q206. Have you ever experienced discrimination, been prevented from doing something, or been hassled or made to feel inferior because of your gender identity or presentation, or race, ethnicity or color:

Getting service in a store or in a restaurant?

EOD9 EOD9
1

0 = No
1 = Yes
7 = Don't Know
8 = Refuse to Answer
9 = Not Applicable

Q207. Was this related to:

EOD9WHY EOD9 why
1

1 = Gender identity or presentation
2 = Race, ethnicity, or color
3 = BOTH gender identity and race/ethnicity
7 = Don't Know
8 = Refuse to Answer
9 = Not Applicable

Q208. Have you ever experienced discrimination, been prevented from doing something, or been hassled or made to feel inferior because of your gender identity or presentation, or race, ethnicity or color:

Getting credit, bank loans, or a mortgage?

EOD10 EOD10
1

0 = No
1 = Yes
7 = Don't Know
8 = Refuse to Answer
9 = Not Applicable

Q209. Was this related to:

EOD10WHY EOD10 why 1

1 = Gender identity or presentation
2 = Race, ethnicity, or color
3 = BOTH gender identity and race/ethnicity
7 = Don't Know
8 = Refuse to Answer
9 = Not Applicable

Q210. Have you ever experienced discrimination, been prevented from doing something, or been hassled or made to feel inferior because of your gender identity or presentation, or race, ethnicity or color:

<u>On the street or in a public setting</u>?

EOD11 EOD11 1

0 = No
1 = Yes
7 = Don't Know
8 = Refuse to Answer
9 = Not Applicable

Q211. Was this related to:

EOD11WHY EOD11 why 1

1 = Gender identity or presentation
2 = Race, ethnicity, or color
3 = BOTH gender identity and race/ethnicity
7 = Don't Know
8 = Refuse to Answer
9 = Not Applicable

Q212. Have you ever experienced discrimination, been prevented from doing something, or been hassled or made to feel inferior because of your gender identity or presentation, or race, ethnicity or color:

<u>From the police or in courts</u>?

EOD12 EOD12 1

0 = No
1 = Yes
7 = Don't Know
8 = Refuse to Answer
9 = Not Applicable

Q213. Was this related to:

EOD12WHY EOD12 why 1

1 = Gender identity or presentation

2 = Race, ethnicity, or color
3 = BOTH gender identity and race/ethnicity
7 = Don't Know
8 = Refuse to Answer
9 = Not Applicable

Q214. Have you ever been verbally abused or harassed because of your gender identity or presentation, or because of your race, ethnicity, or color?

VERBABU Ever verbally abused or harassed
1

0 = No, neither
1 = Yes, because of my gender identity or presentation
2 = Yes, because of my race, ethnicity, or color
3 = Yes, because of BOTH my gender identity/presentation and race/ethnicity/color
7 = Don't Know
8 = Refuse to Answer
9 = Not Applicable

Q215. Have you ever been physically abused or harassed because of your gender identity or presentation, or because of your race, ethnicity, or color?

PHYSABU Ever physically abused or harassed
1

0 = No, neither
1 = Yes, because of my gender identity or presentation
2 = Yes, because of my race, ethnicity, or color
3 = Yes, because of BOTH my gender identity/presentation and race/ethnicity/color
7 = Don't Know
8 = Refuse to Answer
9 = Not Applicable

Q216. In your life, how many times have you been incarcerated? By this I mean spending time in jail, prison, or police lockup.

INCARC Number of times incarcerated
3

0 - 996 = range
997 = Don't Know
998 = Refuse to Answer
999 = Not Applicable

Q217. When were you most recently incarcerated?

INCDATE Date most recently incarcerated
6

Unlimited - Unlimited = mm/yyyy
2097 = Don't Know (Year)
2098 = Refuse to Answer (Year)
2099 = Not Applicable (Year)

Q218. The last time you were incarcerated, how much time did you spend incarcerated?

INCLONG How long incarcerated
2

0 - 90 = (Years)

97 = Don't Know
98 = Refuse to Answer
99 = Not Applicable

Q219. The last time you were incarcerated, where were you housed for the majority of the time? [READ choices. Check ONE.]

INCHOUS Where housed while incarcerated
1

1 = General population
2 = Protective custody
3 = Administrative segregation/solitary confinement
4 = Other
7 = Don't Know
8 = Refuse to Answer
9 = Not Applicable

Q220. Please specify where you were housed during incarceration.

INCHOUSP Specify housed while incarcerated
50

0 - 50 = range
97 = Don't Know
98 = Refuse to Answer
99 = Not Applicable

Q221. The last time you were incarcerated, did you have access to hormones? [READ choices. Check ONE.]

INCHORM Hormone access while incarcerated
1

0 = No, but I didn't need hormones
1 = No, I needed hormones but didn't receive them
2 = Yes, I needed hormones and received them
7 = Don't Know
8 = Refuse to Answer
9 = Not Applicable

Q222. While incarcerated, have you ever been hurt sexually or made to do something sexual that you did not want to do?

INCSEX Sexual violence while incarcerated
1

0 = No
1 = Yes
7 = Don't Know
8 = Refuse to Answer
9 = Not Applicable

Q223. Have you ever been a victim of a transphobic hate crime?

HATE Ever victim of a hate crime
1

0 = No
1 = Yes
7 = Don't Know
8 = Refuse to Answer
9 = Not Applicable

Q224. What type of hate crime? [READ choices. Check ALL that apply.]

HATETYP What type of hate crime
1

0 - 4 = range
7 = Don't Know
8 = Refuse to Answer
9 = Not Applicable

HATETYPA What type of hate crime: Robbery
1

0 = No
1 = Yes
7 = Don't Know
8 = Refuse to Answer
9 = Not Applicable

HATETYPB What type of hate crime: Physical assault
1

0 = No
1 = Yes
7 = Don't Know
8 = Refuse to Answer
9 = Not Applicable

HATETYPC What type of hate crime: Sexual assault
1

0 = No
1 = Yes
7 = Don't Know
8 = Refuse to Answer
9 = Not Applicable

HATETYPD What type of hate crime: Battery with a weapon
1

0 = No
1 = Yes
7 = Don't Know
8 = Refuse to Answer
9 = Not Applicable

Q225. Did you file a police report?

POLICER Filed report for hate crime
1

0 = No, for none
1 = Yes, for some
2 = Yes, for all
7 = Don't Know
8 = Refuse to Answer
9 = Not Applicable

Q226. Have you ever been scared for your physical safety because of the anger or threats of a partner?

IPVPHYS Scared for physical safety with partner
1

0 = No
1 = Yes
7 = Don't Know
8 = Refuse to Answer
9 = Not Applicable

Q227. Has a partner ever hurt you sexually or made you do something sexual that you did not want to do?

IPVSEXL Sexual violence with partner
1

0 = No
1 = Yes
7 = Don't Know
8 = Refuse to Answer
9 = Not Applicable

Q228. Has a partner made you do something that did not agree with your gender identification (e.g. make you hide your make-up)?

IPVGEND Partner treats to gender identity
1

0 = No
1 = Yes
7 = Don't Know
8 = Refuse to Answer
9 = Not Applicable

Q229. In your life, have you ever had any experience that was so frightening, horrible, or upsetting, that in the past month, you:

<u>Have had nightmares about it or thought about it when you did not want to?</u>

PTSD1 PTSD 1
1

0 = No
1 = Yes
7 = Don't Know
8 = Refuse to Answer
9 = Not Applicable

Q230. In your life, have you ever had any experience that was so frightening, horrible, or upsetting, that in the past month, you:

<u>Tried hard not to think about it or went out of your way to avoid situations that reminded you of it?</u>

PTSD2 PTSD 2
1

0 = No
1 = Yes
7 = Don't Know
8 = Refuse to Answer
9 = Not Applicable

Q231. In your life, have you ever had any experience that was so frightening, horrible, or upsetting, that in the past month, you:

<u>Were constantly on guard, watchful, or easily startled?</u>

PTSD3 PTSD 3
1

0 = No
1 = Yes
7 = Don't Know
8 = Refuse to Answer
9 = Not Applicable

Q232. In your life, have you ever had any experience that was so frightening, horrible, or upsetting, that in the past month, you:

<u>Felt numb or detached from others, activities, or your surroundings?</u>

PTSD4 PTSD 4
1

0 = No
1 = Yes
7 = Don't Know
8 = Refuse to Answer
9 = Not Applicable

Q233. [SHOW Flash Card D.] During the last 30 days, how often did you feel tired out for no reason?

K10A K10 A
1

0 = None of the time
1 = A little of the time
2 = Some of the time
3 = Most of the time
4 = All of the time
7 = Don't Know
8 = Refuse to Answer
9 = Not Applicable

Q234. [SHOW Flash Card D.] During the last 30 days, how often did you feel nervous?

K10B K10 B
1

0 = None of the time
1 = A little of the time
2 = Some of the time
3 = Most of the time
4 = All of the time
7 = Don't Know
8 = Refuse to Answer
9 = Not Applicable

Q235. [SHOW Flash Card D.] During the last 30 days, how often did you feel so nervous that nothing could calm you down?

K10C K10 C 1

0 = None of the time
1 = A little of the time
2 = Some of the time
3 = Most of the time
4 = All of the time
7 = Don't Know
8 = Refuse to Answer
9 = Not Applicable

Q236. [SHOW Flash Card D.] During the last 30 days, how often did you feel helpless?

K10D K10 D 1

0 = None of the time
1 = A little of the time
2 = Some of the time
3 = Most of the time
4 = All of the time
7 = Don't Know
8 = Refuse to Answer
9 = Not Applicable

Q237. [SHOW Flash Card D.] During the last 30 days, how often did you feel restless or fidgety?

K10E K10 E 1

0 = None of the time
1 = A little of the time
2 = Some of the time
3 = Most of the time
4 = All of the time
7 = Don't Know
8 = Refuse to Answer
9 = Not Applicable

Q238. [SHOW Flash Card D.] During the last 30 days, how often did you feel so restless you could not sit still?

K10F K10 F 1

0 = None of the time
1 = A little of the time
2 = Some of the time
3 = Most of the time
4 = All of the time
7 = Don't Know
8 = Refuse to Answer
9 = Not Applicable

Q239. [SHOW Flash Card D.] During the last 30 days, how often did you feel depressed?

K10G K10 G 1

0 = None of the time
1 = A little of the time

2 = Some of the time
3 = Most of the time
4 = All of the time
7 = Don't Know
8 = Refuse to Answer
9 = Not Applicable

Q240. [SHOW Flash Card D.] During the last 30 days, how often did you feel that everything was an effort?

K10H K10 H
1

0 = None of the time
1 = A little of the time
2 = Some of the time
3 = Most of the time
4 = All of the time
7 = Don't Know
8 = Refuse to Answer
9 = Not Applicable

Q241. [SHOW Flash Card D.] During the last 30 days, how often did you feel so sad that nothing could cheer you up?

K10I K10 I
1

0 = None of the time
1 = A little of the time
2 = Some of the time
3 = Most of the time
4 = All of the time
7 = Don't Know
8 = Refuse to Answer
9 = Not Applicable

Q242. [SHOW Flash Card D.] During the last 30 days, how often did you feel worthless?

K10J K10 J
1

0 = None of the time
1 = A little of the time
2 = Some of the time
3 = Most of the time
4 = All of the time
7 = Don't Know
8 = Refuse to Answer
9 = Not Applicable

Q243. Have you ever had thoughts or ideations of committing suicide?

SUICIDTH Thoughts of suicide
1

0 = No
1 = Yes
7 = Don't Know
8 = Refuse to Answer
9 = Not Applicable

Q244. Have you ever attempted suicide?

SUICIDAT Attempted suicide
1

0 = No
1 = Yes
7 = Don't Know
8 = Refuse to Answer
9 = Not Applicable

Q245. The first statement is…

[SHOW Flash Card C.] Most people in the Bay Area would discriminate against someone with HIV. Do you…

HIVSTIG1 HIV Stigma 1
1

1 = Strongly disagree
2 = Somewhat disagree
3 = Somewhat agree
4 = Strongly agree
7 = Don't Know
8 = Refuse to Answer
9 = Not Applicable

Q246. [SHOW Flash Card C.] Most people in the Bay Area would support the rights of a person with HIV to live and work wherever they wanted to. Do you…

HIVSTIG2 HIV Stigma 2
1

1 = Strongly disagree
2 = Somewhat disagree
3 = Somewhat agree
4 = Strongly agree
7 = Don't Know
8 = Refuse to Answer
9 = Not Applicable

Q247. [SHOW Flash Card C.] Most people in the Bay Area would not be friends with someone with HIV. Do you…

HIVSTIG3 HIV Stigma 3
1

1 = Strongly disagree
2 = Somewhat disagree
3 = Somewhat agree
4 = Strongly agree
7 = Don't Know
8 = Refuse to Answer
9 = Not Applicable

Q248. [SHOW Flash Card C.] Most people in the Bay Area think that people who got HIV through sex or drug use have gotten what they deserve. Do you…

HIVSTIG4 HIV Stigma 4
1

1 = Strongly disagree

2 = Somewhat disagree
3 = Somewhat agree
4 = Strongly agree
7 = Don't Know
8 = Refuse to Answer
9 = Not Applicable

Q249. Do you smoke cigarettes or use e-cigarettes? [Do NOT read choices. Check ONE.]

SMOKE Do you smoke
1

0 = No, I do not smoke
1 = Yes, I smoke cigarettes only
2 = Yes, I use e-cigarettes only
3 = Yes, I smoke both cigarettes and e-cigarettes
7 = Don't Know
8 = Refuse to Answer
9 = Not Applicable

Q250. [SHOW Flash Card E.] In the past 12 months, how often did you used electronic cigarettes (a.k.a. e-cigarettes, vaporizers)? [Check ONE.]

SMOKE12M Smoked past 12 months
1

0 = Didn't use
1 = Once a month or less
2 = About once a week
3 = Several times a week
4 = About once a day
5 = Several times a day
7 = Don't Know
8 = Refuse to Answer
9 = Not Applicable

Q251. Do you think electronic cigarettes are less harmful than smoking tobacco/cigarettes?

CIGVECIG Electronic cigarette vs smoking harm
1

0 = No
1 = Yes
7 = Don't Know
8 = Refuse to Answer
9 = Not Applicable

Q252. If electronic cigarettes were proven less harmful than cigarettes, how likely is it that you would use electronic cigarettes instead of smoking tobacco/cigarettes? [READ choices. Check ONE.]

ECIGSW Likelihood of using e cig instead of smoking
1

0 = Extremely unlikely
1 = Unlikely
2 = Neither unlikely or likely
3 = Likely
4 = Extremely likely
7 = Don't Know
8 = Refuse to Answer

9 = Not Applicable

Q253. [SHOW Flash Card F.] A drink of alcohol is 1 beer, 1 glass of wine, 1 cocktail, or 1 shot of liquor. In the past 12 months, how often have you had at least 1 drink of alcohol?

ALCUSE How often in past 12 months have you had 1 drink of alcohol 1

0 = Never
1 = Less than once a week
2 = 1-2 days a week
3 = 3-6 days a week
4 = Every day
7 = Don't Know
8 = Refuse to Answer
9 = Not Applicable

Q254. On a typical day when you drank alcohol in the past 12 months, about how many drinks did you have?

NUMDRK Number of drinks on a typical day in past 12 months 2

0 - 96 = range
97 = Don't Know
98 = Refuse to Answer
99 = Not Applicable

Q255. How many times in the past 12 months have you had 5 or more drinks in a day?

MORE5DR How many times in the past 12 months had 5 or more drinks 3

0 - 365 = range
997 = Don't Know
998 = Refuse to Answer
999 = Not Applicable

Q256. In the past 12 months, have you used alcohol just before or during sex?

ALCSEX alcohol before or during sex 1

0 = No
1 = Yes
7 = Don't Know
8 = Refuse to Answer
9 = Not Applicable

Q257. Did you use any substances in the past 12 months?

NIUSE12 Substance use last 12 months 1

0 = No
1 = Yes
7 = Don't Know
8 = Refuse to Answer
9 = Not Applicable

Q258. [SHOW Flash Card G.] In the last 12 months, did you use Marijuana?

MARIJ Marijuana use

1

0 = Didn't use
1 = Less than once a month
2 = 1-3 times a month
3 = One day a week
4 = 2-3 days a week
5 = 4-6 days a week
6 = Every day
7 = Don't Know
8 = Refuse to Answer
9 = Not Applicable

Q259. [SHOW Flash Card G.] In the last 12 months, did you use Methamphetamine (speed, Tina)?

METH Meth use
1

0 = Didn't use
1 = Less than once a month
2 = 1-3 times a month
3 = One day a week
4 = 2-3 days a week
5 = 4-6 days a week
6 = Every day
7 = Don't Know
8 = Refuse to Answer
9 = Not Applicable

Q260. [SHOW Flash Card G.] In the last 12 months, did you use Crack?

CRACK Crack use
1

0 = Didn't use
1 = Less than once a month
2 = 1-3 times a month
3 = One day a week
4 = 2-3 days a week
5 = 4-6 days a week
6 = Every day
7 = Don't Know
8 = Refuse to Answer
9 = Not Applicable

Q261. [SHOW Flash Card G.] In the last 12 months, did you use Cocaine?

COKE Cocaine use
1

0 = Didn't use
1 = Less than once a month
2 = 1-3 times a month
3 = One day a week
4 = 2-3 days a week
5 = 4-6 days a week
6 = Every day
7 = Don't Know
8 = Refuse to Answer
9 = Not Applicable

Q262. [SHOW Flash Card G.] In the last 12 months, did you use downers (like Valium, Ativan, Xanax)?

DOWN Downers use
1

0 = Didn't use
1 = Less than once a month
2 = 1-3 times a month
3 = One day a week
4 = 2-3 days a week
5 = 4-6 days a week
6 = Every day
7 = Don't Know
8 = Refuse to Answer
9 = Not Applicable

Q263. [SHOW Flash Card G.] In the last 12 months, did you use prescription Painkillers?

PAINK Painkiller use
1

0 = Didn't use
1 = Less than once a month
2 = 1-3 times a month
3 = One day a week
4 = 2-3 days a week
5 = 4-6 days a week
6 = Every day
7 = Don't Know
8 = Refuse to Answer
9 = Not Applicable

Q264. [SHOW Flash Card G.] In the last 12 months, did you use Heroin?

HERO Heroin use
1

0 = Didn't use
1 = Less than once a month
2 = 1-3 times a month
3 = One day a week
4 = 2-3 days a week
5 = 4-6 days a week
6 = Every day
7 = Don't Know
8 = Refuse to Answer
9 = Not Applicable

Q265. [SHOW Flash Card G.] In the last 12 months, did you use Ecstasy (Molly, MDMA, MDA)?

ECSTASY Ecstasy use
1

0 = Didn't use
1 = Less than once a month
2 = 1-3 times a month
3 = One day a week
4 = 2-3 days a week
5 = 4-6 days a week
6 = Every day

7 = Don't Know
8 = Refuse to Answer
9 = Not Applicable

Q266. [SHOW Flash Card G.] In the last 12 months, did you use club drugs, like GHB, ketamine, or rohypnol?

CLUBDR Club drugs use
1

0 = Didn't use
1 = Less than once a month
2 = 1-3 times a month
3 = One day a week
4 = 2-3 days a week
5 = 4-6 days a week
6 = Every day
7 = Don't Know
8 = Refuse to Answer
9 = Not Applicable

Q267. [SHOW Flash Card G.] In the last 12 months, did you use Hallucinogens (PCP, LSD, mushrooms, Peyote, Mescaline)?

HALLUC Hallucinogens use
1

0 = Didn't use
1 = Less than once a month
2 = 1-3 times a month
3 = One day a week
4 = 2-3 days a week
5 = 4-6 days a week
6 = Every day
7 = Don't Know
8 = Refuse to Answer
9 = Not Applicable

Q268. [SHOW Flash Card G.] In the last 12 months, did you use Synthetic cannabinoids (K2, Spice)?

SYNTHC Synthetic cannabinoids use
1

0 = Didn't use
1 = Less than once a month
2 = 1-3 times a month
3 = One day a week
4 = 2-3 days a week
5 = 4-6 days a week
6 = Every day
7 = Don't Know
8 = Refuse to Answer
9 = Not Applicable

Q269. [SHOW Flash Card G.] In the last 12 months, did you use poppers?

POPP Poppers use
1

0 = Didn't use

1 = Less than once a month
2 = 1-3 times a month
3 = One day a week
4 = 2-3 days a week
5 = 4-6 days a week
6 = Every day
7 = Don't Know
8 = Refuse to Answer
9 = Not Applicable

Q270. Have you used any other drugs in the past 12 months?

ODRUGYN Other drugs used
1

0 = No
1 = Yes
7 = Don't Know
8 = Refuse to Answer
9 = Not Applicable

Q271. Specify what other drugs you have used in the past 12 months.

ODRUGSP Specify other drugs used in the past 12 months
50

0 - 50 = range
97 = Don't Know
98 = Refuse to Answer
99 = Not Applicable

Q272. [SHOW Flash Card G.] How often have you used [Response to Q271] in the past 12 months?

ODRUG Other drug use
1

0 = Didn't use
1 = Less than once a month
2 = 1-3 times a month
3 = One day a week
4 = 2-3 days a week
5 = 4-6 days a week
6 = Every day
7 = Don't Know
8 = Refuse to Answer
9 = Not Applicable

Q273. Did you use marijuana before or during sex in the last 12 months?

MARIJS Marijuana and sex
1

0 = No
1 = Yes
7 = Don't Know
8 = Refuse to Answer
9 = Not Applicable

Q274. Have you used meth through the following ways in the past 12 months? [Check ALL that apply.]

METHRTE Non-injection meth routes in the past 12 months

1

0 - 4 = range
7 = Don't Know
8 = Refuse to Answer
9 = Not Applicable

METHRTEA Non-injection meth routes in the past 12 months: Ingestion/swallowing
1

0 = No
1 = Yes
7 = Don't Know
8 = Refuse to Answer
9 = Not Applicable

METHRTEB Non-injection meth routes in the past 12 months: Anal/bootie bump
1

0 = No
1 = Yes
7 = Don't Know
8 = Refuse to Answer
9 = Not Applicable

METHRTEC Non-injection meth routes in the past 12 months: Snorting
1

0 = No
1 = Yes
7 = Don't Know
8 = Refuse to Answer
9 = Not Applicable

METHRTED Non-injection meth routes in the past 12 months: Smoking
1

0 = No
1 = Yes
7 = Don't Know
8 = Refuse to Answer
9 = Not Applicable

Q275. Have you injected meth in the past 12 months?

INJMETH Injected meth in the past 12 months
1

0 = No
1 = Yes
7 = Don't Know
8 = Refuse to Answer
9 = Not Applicable

Q276. Did you use methamphetamines before or during sex in the last 12 months?

METHS Meth and sex
1

0 = No
1 = Yes
7 = Don't Know

8 = Refuse to Answer
9 = Not Applicable

Q277. Have you injected crack in the past 12 months?

INJCRACK Injected crack in the past 12 months
1

0 = No
1 = Yes
7 = Don't Know
8 = Refuse to Answer
9 = Not Applicable

Q278. Did you use crack before or during sex in the last 12 months?

CRACKS Crack and sex
1

0 = No
1 = Yes
7 = Don't Know
8 = Refuse to Answer
9 = Not Applicable

Q279. Have you injected cocaine in the past 12 months?

INJCOKE Injected coke in the past 12 months
1

0 = No
1 = Yes
7 = Don't Know
8 = Refuse to Answer
9 = Not Applicable

Q280. Did you use cocaine before or during sex in the last 12 months?

COKES Coke and sex
1

0 = No
1 = Yes
7 = Don't Know
8 = Refuse to Answer
9 = Not Applicable

Q281. Have you injected downers in the past 12 months?

INJDOWN Injected downers in the past 12 months
1

0 = No
1 = Yes
7 = Don't Know
8 = Refuse to Answer
9 = Not Applicable

Q282. Did you use downers before or during sex in the last 12 months?

DOWNS Downers and sex
1

0 = No

1 = Yes
7 = Don't Know
8 = Refuse to Answer
9 = Not Applicable

Q283. Which painkillers have you used in the past 12 months? [Check ALL that apply.]

PAINTYP Which painkillers used in past 12 months
1

0 - 6 = range
7 = Don't Know
8 = Refuse to Answer
9 = Not Applicable

PAINTYPA Which painkillers used in past 12 months: Oxycontin
1

0 = No
1 = Yes
7 = Don't Know
8 = Refuse to Answer
9 = Not Applicable

PAINTYPB Which painkillers used in past 12 months: Percoset
1

0 = No
1 = Yes
7 = Don't Know
8 = Refuse to Answer
9 = Not Applicable

PAINTYPC Which painkillers used in past 12 months: Codeine
1

0 = No
1 = Yes
7 = Don't Know
8 = Refuse to Answer
9 = Not Applicable

PAINTYPD Which painkillers used in past 12 months: Vicodin
1

0 = No
1 = Yes
7 = Don't Know
8 = Refuse to Answer
9 = Not Applicable

PAINTYPE Which painkillers used in past 12 months: Fentanyl
1

0 = No
1 = Yes
7 = Don't Know
8 = Refuse to Answer
9 = Not Applicable

PAINTYPF Which painkillers used in past 12 months: Other

1

0 = No
1 = Yes
7 = Don't Know
8 = Refuse to Answer
9 = Not Applicable

Q284. Please specify which other painkillers you used in the past 12 months

PAINFSP Other painkillers specify
50

0 - 50 = range
97 = Don't Know
98 = Refuse to Answer
99 = Not Applicable

Q285. Have you injected any painkillers in the past 12 months?

INJPAIN Injected painkillers in the past 12 months
1

0 = No
1 = Yes
7 = Don't Know
8 = Refuse to Answer
9 = Not Applicable

Q286. Did you use painkillers before or during sex in the last 12 months?

PAINKS Painkillers and sex
1

0 = No
1 = Yes
7 = Don't Know
8 = Refuse to Answer
9 = Not Applicable

Q287. Have you injected heroin in the past 12 months?

INJHER Injected heroin in the past 12 months
1

0 = No
1 = Yes
7 = Don't Know
8 = Refuse to Answer
9 = Not Applicable

Q288. Did you use heroin before or during sex in the last 12 months?

HEROS Heroin and sex
1

0 = No
1 = Yes
7 = Don't Know
8 = Refuse to Answer
9 = Not Applicable

Q289. Have you injected ecstasy in the past 12 months?

INJECT Injected ecstasy in the past 12 months
1

0 = No
1 = Yes
7 = Don't Know
8 = Refuse to Answer
9 = Not Applicable

Q290. Did you use ecstasy before or during sex in the last 12 months?

ECSTS Ecstasy and sex
1

0 = No
1 = Yes
7 = Don't Know
8 = Refuse to Answer
9 = Not Applicable

Q291. Have you injected club drugs like GHB or ketamine in the past 12 months?

INJCLUB Injected club drugs in the past 12 months
1

0 = No
1 = Yes
7 = Don't Know
8 = Refuse to Answer
9 = Not Applicable

Q292. Did you use club drugs (ketamine, GHB, or rohypnol) before or during sex in the last 12 months?

CLUBDRS Club drugs and sex
1

0 = No
1 = Yes
7 = Don't Know
8 = Refuse to Answer
9 = Not Applicable

Q293. Have you injected hallucinogens in the past 12 months?

INJHALU Injected hallucinogens in the past 12 months
1

0 = No
1 = Yes
7 = Don't Know
8 = Refuse to Answer
9 = Not Applicable

Q294. Did you use hallucinogens before or during sex in the last 12 months?

HALLUCS Hallucinogens and sex
1

0 = No
1 = Yes
7 = Don't Know

8 = Refuse to Answer
9 = Not Applicable

Q295. Did you use synthetic cannabinoids before or during sex in the last 12 months?

SYNTHCS Synthetic cannabinoids and sex
1

0 = No
1 = Yes
7 = Don't Know
8 = Refuse to Answer
9 = Not Applicable

Q296. Did you use poppers before or during sex in the last 12 months?

POPPS Poppers and sex
1

0 = No
1 = Yes
7 = Don't Know
8 = Refuse to Answer
9 = Not Applicable

Q297. Have you injected [Response to Q271] in the past 12 months?

INJOTH Injected other drugs in the past 12 months
1

0 = No
1 = Yes
7 = Don't Know
8 = Refuse to Answer
9 = Not Applicable

Q298. Did you use [Response to Q271] before or during sex in the last 12 months?

ODRUGS Other drugs and sex
1

0 = No
1 = Yes
7 = Don't Know
8 = Refuse to Answer
9 = Not Applicable

Q299. <u>In your life</u>, have you ever used intranasal drugs?

NASALDR Ever used instranasal drugs
1

0 = No
1 = Yes
7 = Don't Know
8 = Refuse to Answer
9 = Not Applicable

Q300. <u>In your life</u>, have you ever used injection drugs?

INJEVER Ever used injection drugs
1

0 = No

1 = Yes
7 = Don't Know
8 = Refuse to Answer
9 = Not Applicable

Q301. In your life, have you ever smoked crack or methampetamine?

EVERSMOK Ever smoked crack or methamphetamines
1

0 = No
1 = Yes
7 = Don't Know
8 = Refuse to Answer
9 = Not Applicable

Calculated Variable

INJ12MO

INJ12MO = IF(INJMETH=1, 1,IF(INJCOKE=1, 1,IF(INJCRACK=1, 1,IF(INJDOWN=1, 1,IF(INJHER=1, 1,IF(INJHALU=1, 1,IF(INJECT=1, 1,IF(INJCLUB=1, 1,IF(INJPAIN=1, 1,IF(INJOTH=1,1,0))))))))))

Q302. During the past 12 months, how often did you use needles that someone else had already injected with? [READ choices. Check ONE.]

ELSEND In past 12 months, how often used needles used by someone else
1

0 = Never
1 = Rarely
2 = About half the time
3 = Most of the time
4 = Always
7 = Don't Know
8 = Refuse to Answer
9 = Not Applicable

Q303. In the past 12 months, with how many people did you use a needle after they injected with it?

SHAREP Past 12 months, how many people shared with
3

0 - 996 = range
997 = Don't Know
998 = Refuse to Answer
999 = Not Applicable

Q304. In the past 12 months, how many of the people you shared needles with did you also have sex with?

SHARESX Past 12 months, how many people shared with did you also have sex with
3

0 - 996 = range
997 = Don't Know
998 = Refuse to Answer
999 = Not Applicable

Q305. Have you ever participated in an alcohol or drug treatment program?

DRUGTR Ever participated in alcohol/drug treatment program
1

0 = No
1 = Yes
7 = Don't Know
8 = Refuse to Answer
9 = Not Applicable

Q306. In the past 12 months have you participated in an alcohol or drug treatment program?

DRUGTR12 Drug treatment in past 12 months
1

0 = No
1 = Yes
7 = Don't Know
8 = Refuse to Answer
9 = Not Applicable

Q307. Have you ever wanted to participate in an alcohol or drug treatment program but been unable to?

WANTTRT Ever wanted to participate in alcohol/drug treatment
1

0 = No
1 = Yes
7 = Don't Know
8 = Refuse to Answer
9 = Not Applicable

Q308. Have your parents or immediate caregivers ever had a drinking or drug problem that got in the way of their work and/or relationships?

PARENTDR Parents or caregivers had drinking or drug problem
1

0 = No
1 = Yes
7 = Don't Know
8 = Refuse to Answer
9 = Not Applicable

Calculated Variable

NOPENIS No penis past 6 months (Had SRS)

NOPENIS = IF((TYPSURGD=1 AND IDATE-WNSURGD>=183),1,IF((TYPSURGD=1 AND IDATE-WNSURGD<183),2,0))

Q309. With how many people did you have sex with in the past 6 months (since [SIXM])?

SEXPEEP How many sex partners last 6 months
3

0 - 996 = range
997 = Don't Know
998 = Refuse to Answer
999 = Not Applicable

Q310. You indicated that you have had zero sexual partners, is that correct?

SEXPCONF Sex partner confirmation
1

0 = No
1 = Yes
7 = Don't Know
8 = Refuse to Answer
9 = Not Applicable

Q311. With how many of these [Response to Q309] people did you have only oral or non-penetrative sex?

SEXORAL Oral sex only
3

0 - 996 = range
997 = Don't Know
998 = Refuse to Answer
999 = Not Applicable

Calculated Variable

VAGANSEX All sex minus oral sex

VAGANSEX = SEXPEEP-SEXORAL

Q312. How many gender non-binary or gender non-conforming partners have you had vaginal or anal sex with in the past 6 months?

GNCSEX Gender non binary sex
3

0 - 996 = range
997 = Don't Know
998 = Refuse to Answer
999 = Not Applicable

Q313. How many transgender women have you had vaginal or anal sex with in the past 6 months? (MTF-transgender/transfemales)

MTFSEX MTF-transgender sex
3

0 - 996 = range
997 = Don't Know
998 = Refuse to Answer
999 = Not Applicable

Q314. How many transgender men have you had vaginal or anal sex with in the past 6 months? (FTM-transgender/transmales)

FTMSEX FTM-transgender sex
3

0 - 996 = range
997 = Don't Know
998 = Refuse to Answer
999 = Not Applicable

Q315. How many cisgender men have you had vaginal or anal sex with in the past 6 months? (Men)

CISMSEX Cisgender man sex
3

0 - 996 = range
997 = Don't Know
998 = Refuse to Answer
999 = Not Applicable

Q316. How many cisgender women have you had vaginal or anal sex with in the past 6 months? (Woman)

CISWSEX Cisgender woman sex
3

0 - 996 = range
997 = Don't Know
998 = Refuse to Answer
999 = Not Applicable

Calculated Variable

TOTAVPAR Total anal vaginal sex partner

TOTAVPAR = (MTFSEX)+(FTMSEX)+(CISMSEX)+(CISWSEX)+(GNCSEX)

Q317. To help guide you through the questions, please provide initials for this first person: we will refer to this person later using the initials you provide.

P1IN P1 Initials
20

0 - 20 = range
97 = Don't Know
98 = Refuse to Answer
99 = Not Applicable

Q318. What is [Response to Q317]s gender? [Do NOT read choices. Check ONE.]

P1GEN P1 gender
2

1 = Cisgender male
2 = Cisgender female
3 = Transgender female / transwoman (Male to female transgender)
4 = Transgender male/transman (Female to male transgender)
5 = Androgynous/ambigender
6 = Questioning
7 = Genderqueer/genderfluid
8 = Other
97 = Don't Know
98 = Refuse to Answer
99 = Not Applicable

Q319. Did [Response to Q317] have a penis?

P1PENIS P1 has penis
1

0 = No
1 = Yes
7 = Don't Know
8 = Refuse to Answer
9 = Not Applicable

Q320. How old was [Response to Q317]?

P1AGE P1 Age 2

- **0 - 96** = range
- **97** = Don't Know
- **98** = Refuse to Answer
- **99** = Not Applicable

Q321. What type of partner was [Response to Q317]?

Main Partner: Someone who is your primary sexual partner and you feel committed to (boyfriend, lover, husband, girlfriend, wife).

Casual Partner: Someone you have sex with, but don't feel committed to or don't know very well.

Exchange partner/Trick: Someone you had sex with in exchange for things like money, goods, or a place to stay.

P1TYPE P1 type 1

- **1** = Main partner
- **2** = Casual partner
- **3** = Exchange partner / Trick
- **4** = Sexual assault
- **7** = Don't Know
- **8** = Refuse to Answer
- **9** = Not Applicable

Q322. Did this partner pay you or did you pay this partner?

P1PAYOR P1 paid/you paid partner 1

- **1** = I was paid
- **2** = I paid this partner
- **7** = Don't Know
- **8** = Refuse to Answer
- **9** = Not Applicable

Q323. Would you consider this partner to be trade?

P1TRADE P1 considered trade 1

- **0** = No
- **1** = Yes
- **7** = Don't Know
- **8** = Refuse to Answer
- **9** = Not Applicable

Q324. What is [Response to Q317]'s race or ethnicity? Would you describe [Response to Q317] as:

[Do NOT read choices. Check ALL that apply]

P1RACE P1 race 2

- **0 - 7** = range
- **97** = Don't Know
- **98** = Refuse to Answer
- **99** = Not Applicable

P1RACEA P1 race: Asian

1

0 = No
1 = Yes
7 = Don't Know
8 = Refuse to Answer
9 = Not Applicable

P1RACEB P1 race: Black / African American

1

0 = No
1 = Yes
7 = Don't Know
8 = Refuse to Answer
9 = Not Applicable

P1RACEC P1 race: Native American

1

0 = No
1 = Yes
7 = Don't Know
8 = Refuse to Answer
9 = Not Applicable

P1RACED P1 race: Native Hawaiian or Pacific Islander

1

0 = No
1 = Yes
7 = Don't Know
8 = Refuse to Answer
9 = Not Applicable

P1RACEE P1 race: White

1

0 = No
1 = Yes
7 = Don't Know
8 = Refuse to Answer
9 = Not Applicable

P1RACEF P1 race: Latino/Latina

1

0 = No
1 = Yes
7 = Don't Know
8 = Refuse to Answer
9 = Not Applicable

P1RACEG P1 race: Other

1

0 = No
1 = Yes
7 = Don't Know
8 = Refuse to Answer

9 = Not Applicable

Q325. Please specify race / ethnicity:

P1RACESP P1 other race
20

0 - 20 = range
97 = Don't Know
98 = Refuse to Answer
99 = Not Applicable

Q326. To your knowledge, in the past 6 months has [Response to Q317] had sex with other people who are:

[READ choices. Check ALL that apply.]

P1SEXP P1's sex partners
1

0 - 6 = range
7 = Don't Know
8 = Refuse to Answer
9 = Not Applicable

P1SEXPA P1's sex partners: Cisgender men
1

0 = No
1 = Yes
7 = Don't Know
8 = Refuse to Answer
9 = Not Applicable

P1SEXPB P1's sex partners: Cisgender women
1

0 = No
1 = Yes
7 = Don't Know
8 = Refuse to Answer
9 = Not Applicable

P1SEXPC P1's sex partners: Transgender men
1

0 = No
1 = Yes
7 = Don't Know
8 = Refuse to Answer
9 = Not Applicable

P1SEXPD P1's sex partners: Transgender women
1

0 = No
1 = Yes
7 = Don't Know
8 = Refuse to Answer
9 = Not Applicable

P1SEXPE P1's sex partners: Gender non-conforming or gender non-binary

1

0 = No
1 = Yes
7 = Don't Know
8 = Refuse to Answer
9 = Not Applicable

P1SEXPF P1's sex partners: None
1

0 = No
1 = Yes
7 = Don't Know
8 = Refuse to Answer
9 = Not Applicable

Q327. Where did you first meet [Response to Q317]? [Do NOT read choices. Check ONE.]

P1MET P1 where met
2

1 = Bar
2 = Cafe or restaurant
3 = Dance club
4 = Adult book/video store, retail, business
5 = Sex club
6 = Street, park, library, public, transportation
7 = Church, political function, social club
8 = Work or school
9 = Gym or athletic activity
10 = Online - Dating app (OKCupid, Tinder, etc.)
11 = Online - Craigslist
12 = Online - Other, please specify
13 = Dating services or newspaper
14 = Private party or social club
15 = Introduce by friends
16 = Some other way.
97 = Don't Know
98 = Refuse to Answer
99 = Not Applicable

Q328. Please specify on where online you have met [Response to Q317]:

P1METOSP P1 met online specify
50

0 - 50 = range
97 = Don't Know
98 = Refuse to Answer
99 = Not Applicable

Q329. Please specify on where you have met [Response to Q317]:

P1METSP P1 met specify
50

0 - 50 = range
97 = Don't Know
98 = Refuse to Answer

99 = Not Applicable

Q330. To the best of your knowledge what is [Response to Q317]'s HIV status?

P1SERO P1 sero status
1

1 = Negative
2 = Positive
3 = Unknown
7 = Don't Know
8 = Refuse to Answer
9 = Not Applicable

Q331. To your knowledge, was this partner taking PrEP? (anti-HIV medications)

P1PREP P1 taking prep
1

0 = No
1 = Yes
7 = Don't Know
8 = Refuse to Answer
9 = Not Applicable

Q332. To your knowledge, was this partner on antiretrovirals?

P1ART P1 on ART
1

0 = No
1 = Yes
7 = Don't Know
8 = Refuse to Answer
9 = Not Applicable

Q333. To your knowledge, was this partner virally suppressed or their viral load undetectable?

P1VIRSUP P1 virally suppressed
1

0 = No
1 = Yes
7 = Don't Know
8 = Refuse to Answer
9 = Not Applicable

Q334. Did you disclose your HIV status to this partner before having sex the first time?

P1DISCL Disclosed HIV status to P1
1

0 = No
1 = Yes
7 = Don't Know
8 = Refuse to Answer
9 = Not Applicable

Q335. Did you have vaginal sex with [Response to Q317] during the last 6 months?

P1VAG P1 vaginal sex
1

0 = No

1 = Yes
7 = Don't Know
8 = Refuse to Answer
9 = Not Applicable

Q336. How many times have you had insertive vaginal intercourse with [Response to Q317] in the past six months?

P1VAGI P1 # vaginal insertive sex
3

0 - 996 = range
997 = Don't Know
998 = Refuse to Answer
999 = Not Applicable

Q337. Of the [Response to Q336] times you had insertive vaginal sex with this partner,

how many times did you NOT use a condom?

P1VAGIC P1 # vag ins no condom
3

0 - 996 = range
997 = Don't Know
998 = Refuse to Answer
999 = Not Applicable

Q338. Of the [Response to Q337] times you had insertive vaginal sex without a condom,

how many times were you high or drunk?

P1VAGIH P1 # vag ins unprot sex high or drunk
3

0 - 996 = range
997 = Don't Know
998 = Refuse to Answer
999 = Not Applicable

Q339. Now think of the last time you had insertive vaginal sex with [Response to Q317], did you use a condom?

P1VAGICL P1 last ins vag no condom
1

0 = No
1 = Yes
7 = Don't Know
8 = Refuse to Answer
9 = Not Applicable

Q340. Were you drinking or using drugs in the 2 hours before or during the last time you had insertive vaginal sex?

P1VAGIHL P1 last vag ins sex high or drunk
1

0 = No
1 = Yes
7 = Don't Know
8 = Refuse to Answer
9 = Not Applicable

Q341. What substances were you using?

P1VISUB P1 last vag ins what substances 2

0 - 18 = range
97 = Don't Know
98 = Refuse to Answer
99 = Not Applicable

P1VISUBA P1 last vag ins what substances: Alcohol 1

0 = No
1 = Yes
7 = Don't Know
8 = Refuse to Answer
9 = Not Applicable

P1VISUBB P1 last vag ins what substances: Meth 1

0 = No
1 = Yes
7 = Don't Know
8 = Refuse to Answer
9 = Not Applicable

P1VISUBC P1 last vag ins what substances: Crack 1

0 = No
1 = Yes
7 = Don't Know
8 = Refuse to Answer
9 = Not Applicable

P1VISUBD P1 last vag ins what substances: Cocaine 1

0 = No
1 = Yes
7 = Don't Know
8 = Refuse to Answer
9 = Not Applicable

P1VISUBE P1 last vag ins what substances: Poppers 1

0 = No
1 = Yes
7 = Don't Know
8 = Refuse to Answer
9 = Not Applicable

P1VISUBF P1 last vag ins what substances: GHB 1

0 = No
1 = Yes
7 = Don't Know
8 = Refuse to Answer

9 = Not Applicable

P1VISUBG P1 last vag ins what substances: Heroin 1

0 = No
1 = Yes
7 = Don't Know
8 = Refuse to Answer
9 = Not Applicable

P1VISUBH P1 last vag ins what substances: Marijuana 1

0 = No
1 = Yes
7 = Don't Know
8 = Refuse to Answer
9 = Not Applicable

P1VISUBI P1 last vag ins what substances: Synthetic cannabinoids 1

0 = No
1 = Yes
7 = Don't Know
8 = Refuse to Answer
9 = Not Applicable

P1VISUBJ P1 last vag ins what substances: Hallucinogens 1

0 = No
1 = Yes
7 = Don't Know
8 = Refuse to Answer
9 = Not Applicable

P1VISUBK P1 last vag ins what substances: Ecstasy 1

0 = No
1 = Yes
7 = Don't Know
8 = Refuse to Answer
9 = Not Applicable

P1VISUBL P1 last vag ins what substances: Rohypnol (roofies) 1

0 = No
1 = Yes
7 = Don't Know
8 = Refuse to Answer
9 = Not Applicable

P1VISUBM P1 last vag ins what substances: Ketamine 1

0 = No
1 = Yes

7 = Don't Know
8 = Refuse to Answer
9 = Not Applicable

P1VISUBN P1 last vag ins what substances: Benzos or barbituates
1

0 = No
1 = Yes
7 = Don't Know
8 = Refuse to Answer
9 = Not Applicable

P1VISUBO P1 last vag ins what substances: Painkillers
1

0 = No
1 = Yes
7 = Don't Know
8 = Refuse to Answer
9 = Not Applicable

P1VISUBP P1 last vag ins what substances: Viagra
1

0 = No
1 = Yes
7 = Don't Know
8 = Refuse to Answer
9 = Not Applicable

P1VISUBQ P1 last vag ins what substances: Prescription drugs
1

0 = No
1 = Yes
7 = Don't Know
8 = Refuse to Answer
9 = Not Applicable

P1VISUBR P1 last vag ins what substances: Other
1

0 = No
1 = Yes
7 = Don't Know
8 = Refuse to Answer
9 = Not Applicable

Q342. How many times have you had receptive vaginal intercourse with [Response to Q317] in the past six months?

P1VAGR P1 # vaginal receptive sex
3

0 - 996 = range
997 = Don't Know
998 = Refuse to Answer
999 = Not Applicable

Q343. Of the [Response to Q342] times you had receptive vaginal sex, how many times did you NOT use a condom?

P1VAGRC P1 # vag rec no condom 3

0 - 996 = range
997 = Don't Know
998 = Refuse to Answer
999 = Not Applicable

Q344. Of the [Response to Q343] times you had receptive vaginal sex without a condom, how many times were you high or drunk?

P1VAGRH P1 # vag rec unprot sex high or drunk 3

0 - 996 = range
997 = Don't Know
998 = Refuse to Answer
999 = Not Applicable

Q345. Now think of the last time you had receptive vaginal sex with [Response to Q317], did you use a condom?

P1VAGRCL P1 last rec vag no condom 1

0 = No
1 = Yes
7 = Don't Know
8 = Refuse to Answer
9 = Not Applicable

Q346. Were you drinking or using drugs in the 2 hours before or during the last time you had receptive vaginal sex?

P1VAGRHL P1 last vag rec sex high or drunk 1

0 = No
1 = Yes
7 = Don't Know
8 = Refuse to Answer
9 = Not Applicable

Q347. What substances were you using?

P1VRSUB P1 last vag rec what substances 2

0 - 18 = range
97 = Don't Know
98 = Refuse to Answer
99 = Not Applicable

P1VRSUBA P1 last vag rec what substances: Alcohol 1

0 = No
1 = Yes
7 = Don't Know
8 = Refuse to Answer
9 = Not Applicable

P1VRSUBB P1 last vag rec what substances: Meth
1

0 = No
1 = Yes
7 = Don't Know
8 = Refuse to Answer
9 = Not Applicable

P1VRSUBC P1 last vag rec what substances: Crack
1

0 = No
1 = Yes
7 = Don't Know
8 = Refuse to Answer
9 = Not Applicable

P1VRSUBD P1 last vag rec what substances: Cocaine
1

0 = No
1 = Yes
7 = Don't Know
8 = Refuse to Answer
9 = Not Applicable

P1VRSUBE P1 last vag rec what substances: Poppers
1

0 = No
1 = Yes
7 = Don't Know
8 = Refuse to Answer
9 = Not Applicable

P1VRSUBF P1 last vag rec what substances: GHB
1

0 = No
1 = Yes
7 = Don't Know
8 = Refuse to Answer
9 = Not Applicable

P1VRSUBG P1 last vag rec what substances: Heroin
1

0 = No
1 = Yes
7 = Don't Know
8 = Refuse to Answer
9 = Not Applicable

P1VRSUBH P1 last vag rec what substances: Marijuana
1

0 = No
1 = Yes
7 = Don't Know

8 = Refuse to Answer
9 = Not Applicable

P1VRSUBI P1 last vag rec what substances: Synthetic cannabinoids
1

0 = No
1 = Yes
7 = Don't Know
8 = Refuse to Answer
9 = Not Applicable

P1VRSUBJ P1 last vag rec what substances: Hallucinogens
1

0 = No
1 = Yes
7 = Don't Know
8 = Refuse to Answer
9 = Not Applicable

P1VRSUBK P1 last vag rec what substances: Ecstasy
1

0 = No
1 = Yes
7 = Don't Know
8 = Refuse to Answer
9 = Not Applicable

P1VRSUBL P1 last vag rec what substances: Rohypnol (roofies)
1

0 = No
1 = Yes
7 = Don't Know
8 = Refuse to Answer
9 = Not Applicable

P1VRSUBM P1 last vag rec what substances: Ketamine
1

0 = No
1 = Yes
7 = Don't Know
8 = Refuse to Answer
9 = Not Applicable

P1VRSUBN P1 last vag rec what substances: Benzos or barbituates
1

0 = No
1 = Yes
7 = Don't Know
8 = Refuse to Answer
9 = Not Applicable

P1VRSUBO P1 last vag rec what substances: Painkillers
1

0 = No

1 = Yes
7 = Don't Know
8 = Refuse to Answer
9 = Not Applicable

P1VRSUBP P1 last vag rec what substances: Viagra
1

0 = No
1 = Yes
7 = Don't Know
8 = Refuse to Answer
9 = Not Applicable

P1VRSUBQ P1 last vag rec what substances: Prescription drugs
1

0 = No
1 = Yes
7 = Don't Know
8 = Refuse to Answer
9 = Not Applicable

P1VRSUBR P1 last vag rec what substances: Other
1

0 = No
1 = Yes
7 = Don't Know
8 = Refuse to Answer
9 = Not Applicable

Q348. Did you have anal sex with [Response to Q317] during the last 6 months?

P1ANAL P1 anal sex
1

0 = No
1 = Yes
7 = Don't Know
8 = Refuse to Answer
9 = Not Applicable

Q349. How many times have you had insertive anal intercourse with [Response to Q317] in the past 6 months?

P1ANAI P1 # insertive anal sex
3

0 - 996 = range
997 = Don't Know
998 = Refuse to Answer
999 = Not Applicable

Q350. Of the [Response to Q349] times you had insertive anal sex,

how many times did you NOT use a condom?

P1ANAIC P1 # anal ins no condom
3

0 - 996 = range

997 = Don't Know
998 = Refuse to Answer
999 = Not Applicable

Q351. Of the [Response to Q350] times you had insertive anal sex without a condom, how many times were you high or drunk?

P1ANAIH P1 # anal ins unprot sex high or drunk
3

0 - 996 = range
997 = Don't Know
998 = Refuse to Answer
999 = Not Applicable

Q352. Now think of the last time you had insertive anal sex with [Response to Q317], did you use a condom?

P1ANAICL P1 last ins anal no condom
1

0 = No
1 = Yes
7 = Don't Know
8 = Refuse to Answer
9 = Not Applicable

Q353. Were you drinking or using drugs in the 2 hours before or during the last time you had insertive anal sex?

P1ANAIHL P1 last anal ins sex high or drunk
1

0 = No
1 = Yes
7 = Don't Know
8 = Refuse to Answer
9 = Not Applicable

Q354. What substances were you using?

P1AISUB P1 last anal ins what substances
2

0 - 18 = range
97 = Don't Know
98 = Refuse to Answer
99 = Not Applicable

P1AISUBA P1 last anal ins what substances: Alcohol
1

0 = No
1 = Yes
7 = Don't Know
8 = Refuse to Answer
9 = Not Applicable

P1AISUBB P1 last anal ins what substances: Meth
1

0 = No
1 = Yes

7 = Don't Know
8 = Refuse to Answer
9 = Not Applicable

P1AISUBC P1 last anal ins what substances: Crack
1

0 = No
1 = Yes
7 = Don't Know
8 = Refuse to Answer
9 = Not Applicable

P1AISUBD P1 last anal ins what substances: Cocaine
1

0 = No
1 = Yes
7 = Don't Know
8 = Refuse to Answer
9 = Not Applicable

P1AISUBE P1 last anal ins what substances: Poppers
1

0 = No
1 = Yes
7 = Don't Know
8 = Refuse to Answer
9 = Not Applicable

P1AISUBF P1 last anal ins what substances: GHB
1

0 = No
1 = Yes
7 = Don't Know
8 = Refuse to Answer
9 = Not Applicable

P1AISUBG P1 last anal ins what substances: Heroin
1

0 = No
1 = Yes
7 = Don't Know
8 = Refuse to Answer
9 = Not Applicable

P1AISUBH P1 last anal ins what substances: Marijuana
1

0 = No
1 = Yes
7 = Don't Know
8 = Refuse to Answer
9 = Not Applicable

P1AISUBI P1 last anal ins what substances: Synthetic cannabinoids
1

0 = No
1 = Yes
7 = Don't Know
8 = Refuse to Answer
9 = Not Applicable

P1AISUBJ P1 last anal ins what substances: Hallucinogens
1

0 = No
1 = Yes
7 = Don't Know
8 = Refuse to Answer
9 = Not Applicable

P1AISUBK P1 last anal ins what substances: Ecstasy
1

0 = No
1 = Yes
7 = Don't Know
8 = Refuse to Answer
9 = Not Applicable

P1AISUBL P1 last anal ins what substances: Rohypnol (roofies)
1

0 = No
1 = Yes
7 = Don't Know
8 = Refuse to Answer
9 = Not Applicable

P1AISUBM P1 last anal ins what substances: Ketamine
1

0 = No
1 = Yes
7 = Don't Know
8 = Refuse to Answer
9 = Not Applicable

P1AISUBN P1 last anal ins what substances: Benzos or barbituates
1

0 = No
1 = Yes
7 = Don't Know
8 = Refuse to Answer
9 = Not Applicable

P1AISUBO P1 last anal ins what substances: Painkillers
1

0 = No
1 = Yes
7 = Don't Know
8 = Refuse to Answer
9 = Not Applicable

P1AISUBP P1 last anal ins what substances: Viagra 1

0 = No
1 = Yes
7 = Don't Know
8 = Refuse to Answer
9 = Not Applicable

P1AISUBQ P1 last anal ins what substances: Prescription drugs 1

0 = No
1 = Yes
7 = Don't Know
8 = Refuse to Answer
9 = Not Applicable

P1AISUBR P1 last anal ins what substances: Other 1

0 = No
1 = Yes
7 = Don't Know
8 = Refuse to Answer
9 = Not Applicable

Q355. How many times have you had receptive anal intercourse with [Response to Q317] in the past six months.

P1ANAR P1 # receptive anal sex 3

0 - 996 = range
997 = Don't Know
998 = Refuse to Answer
999 = Not Applicable

Q356. Of the [Response to Q355] times you had receptive anal sex,
how many times did you NOT use a condom?

P1ANARC P1 # anal rec no condom 3

0 - 996 = range
997 = Don't Know
998 = Refuse to Answer
999 = Not Applicable

Q357. Of the [Response to Q356] times you had receptive anal sex without a condom,
how many times were you high or drunk?

P1ANARH P1 # anal rec unprot sex high or drunk 3

0 - 996 = range
997 = Don't Know
998 = Refuse to Answer
999 = Not Applicable

Q358. Now think of the last time you had receptive anal sex with [Response to Q317], did you use a condom?

P1ANARCL P1 last rec anal no condom 1

0 = No
1 = Yes
7 = Don't Know
8 = Refuse to Answer
9 = Not Applicable

Q359. Were you drinking or using drugs in the 2 hours before or during the last time you had receptive anal sex?

P1ANARHL P1 last anal rec sex high or drunk 1

0 = No
1 = Yes
7 = Don't Know
8 = Refuse to Answer
9 = Not Applicable

Q360. What substances were you using?

P1ARSUB P1 last anal rec what substances 2

0 - 18 = range
97 = Don't Know
98 = Refuse to Answer
99 = Not Applicable

P1ARSUBA P1 last anal rec what substances: Alcohol 1

0 = No
1 = Yes
7 = Don't Know
8 = Refuse to Answer
9 = Not Applicable

P1ARSUBB P1 last anal rec what substances: Meth 1

0 = No
1 = Yes
7 = Don't Know
8 = Refuse to Answer
9 = Not Applicable

P1ARSUBC P1 last anal rec what substances: Crack 1

0 = No
1 = Yes
7 = Don't Know
8 = Refuse to Answer
9 = Not Applicable

P1ARSUBD P1 last anal rec what substances: Cocaine 1

0 = No
1 = Yes
7 = Don't Know
8 = Refuse to Answer
9 = Not Applicable

P1ARSUBE P1 last anal rec what substances: Poppers
1

0 = No
1 = Yes
7 = Don't Know
8 = Refuse to Answer
9 = Not Applicable

P1ARSUBF P1 last anal rec what substances: GHB
1

0 = No
1 = Yes
7 = Don't Know
8 = Refuse to Answer
9 = Not Applicable

P1ARSUBG P1 last anal rec what substances: Heroin
1

0 = No
1 = Yes
7 = Don't Know
8 = Refuse to Answer
9 = Not Applicable

P1ARSUBH P1 last anal rec what substances: Marijuana
1

0 = No
1 = Yes
7 = Don't Know
8 = Refuse to Answer
9 = Not Applicable

P1ARSUBI P1 last anal rec what substances: Synthetic cannabinoids
1

0 = No
1 = Yes
7 = Don't Know
8 = Refuse to Answer
9 = Not Applicable

P1ARSUBJ P1 last anal rec what substances: Hallucinogens
1

0 = No
1 = Yes
7 = Don't Know
8 = Refuse to Answer
9 = Not Applicable

P1ARSUBK P1 last anal rec what substances: Ecstasy
1

0 = No
1 = Yes
7 = Don't Know
8 = Refuse to Answer
9 = Not Applicable

P1ARSUBL P1 last anal rec what substances: Rohypnol (roofies)
1

0 = No
1 = Yes
7 = Don't Know
8 = Refuse to Answer
9 = Not Applicable

P1ARSUBM P1 last anal rec what substances: Ketamine
1

0 = No
1 = Yes
7 = Don't Know
8 = Refuse to Answer
9 = Not Applicable

P1ARSUBN P1 last anal rec what substances: Benzos or barbituates
1

0 = No
1 = Yes
7 = Don't Know
8 = Refuse to Answer
9 = Not Applicable

P1ARSUBO P1 last anal rec what substances: Painkillers
1

0 = No
1 = Yes
7 = Don't Know
8 = Refuse to Answer
9 = Not Applicable

P1ARSUBP P1 last anal rec what substances: Viagra
1

0 = No
1 = Yes
7 = Don't Know
8 = Refuse to Answer
9 = Not Applicable

P1ARSUBQ P1 last anal rec what substances: Prescription drugs
1

0 = No
1 = Yes
7 = Don't Know
8 = Refuse to Answer

9 = Not Applicable

P1ARSUBR P1 last anal rec what substances: Other 1

0 = No
1 = Yes
7 = Don't Know
8 = Refuse to Answer
9 = Not Applicable

Q361. Does this partner inject drugs?

P1INJ P1 inject drugs 1

0 = No
1 = Yes
7 = Don't Know
8 = Refuse to Answer
9 = Not Applicable

Q362. To help guide you through the questions, please provide initials for this next person: we will refer to this person later using the initials you provide.

P2IN P2 Initials 20

0 - 20 = range
97 = Don't Know
98 = Refuse to Answer
99 = Not Applicable

Q363. What is [Response to Q362]s gender? [Do NOT read choices. Check ONE.]

P2GEN P2 gender 2

1 = Cisgender male
2 = Cisgender female
3 = Transgender female / transwoman (Male to female transgender)
4 = Transgender male/transman (Female to male transgender)
5 = Androgynous/ambigender
6 = Questioning
7 = Genderqueer/genderfluid
8 = Other
97 = Don't Know
98 = Refuse to Answer
99 = Not Applicable

Q364. Did [Response to Q362] have a penis?

P2PENIS P2 has penis 1

0 = No
1 = Yes
7 = Don't Know
8 = Refuse to Answer

9 = Not Applicable

Q365. How old was [Response to Q362]?

P2AGE P2 Age 2

0 - 96 = range
97 = Don't Know
98 = Refuse to Answer
99 = Not Applicable

Q366. What type of partner was [Response to Q362]?

Main Partner: Someone who is your primary sexual partner and you feel committed to (boyfriend, lover, husband, girlfriend, wife).

Casual Partner: Someone you have sex with, but don't feel committed to or don't know very well.

Exchange partner/Trick: Someone you had sex with in exchange for things like money, goods, or a place to stay.

P2TYPE P2 type 1

1 = Main partner
2 = Casual partner
3 = Exchange partner / Trick
4 = Sexual assault
7 = Don't Know
8 = Refuse to Answer
9 = Not Applicable

Q367. Did this partner pay you or did you pay this partner?

P2PAYOR P2 paid/you paid partner 1

1 = I was paid
2 = I paid this partner
7 = Don't Know
8 = Refuse to Answer
9 = Not Applicable

Q368. Would you consider this partner to be trade?

P2TRADE P2 considered trade 1

0 = No
1 = Yes
7 = Don't Know
8 = Refuse to Answer
9 = Not Applicable

Q369. What is [Response to Q362]'s race or ethnicity? Would you describe [Response to Q362] as:

[Do NOT read choices. Check ALL that apply]

P2RACE P2 race 2

0 - 7 = range
97 = Don't Know
98 = Refuse to Answer

99 = Not Applicable

P2RACEA P2 race: Asian
1

0 = No
1 = Yes
7 = Don't Know
8 = Refuse to Answer
9 = Not Applicable

P2RACEB P2 race: Black / African American
1

0 = No
1 = Yes
7 = Don't Know
8 = Refuse to Answer
9 = Not Applicable

P2RACEC P2 race: Native American
1

0 = No
1 = Yes
7 = Don't Know
8 = Refuse to Answer
9 = Not Applicable

P2RACED P2 race: Native Hawaiian or Pacific Islander
1

0 = No
1 = Yes
7 = Don't Know
8 = Refuse to Answer
9 = Not Applicable

P2RACEE P2 race: White
1

0 = No
1 = Yes
7 = Don't Know
8 = Refuse to Answer
9 = Not Applicable

P2RACEF P2 race: Latino/Latina
1

0 = No
1 = Yes
7 = Don't Know
8 = Refuse to Answer
9 = Not Applicable

P2RACEG P2 race: Other
1

0 = No
1 = Yes
7 = Don't Know

8 = Refuse to Answer
9 = Not Applicable

Q370. Please specify race / ethnicity:

P2RACESP P2 other race
20

0 - 20 = range
97 = Don't Know
98 = Refuse to Answer
99 = Not Applicable

Q371. To your knowledge, in the past 6 months has [Response to Q362] had sex with other people who are:

[READ choices. Check ALL that apply.]

P2SEXP P2's sex partners
1

0 - 6 = range
7 = Don't Know
8 = Refuse to Answer
9 = Not Applicable

P2SEXPA P2's sex partners: Cisgender men
1

0 = No
1 = Yes
7 = Don't Know
8 = Refuse to Answer
9 = Not Applicable

P2SEXPB P2's sex partners: Cisgender women
1

0 = No
1 = Yes
7 = Don't Know
8 = Refuse to Answer
9 = Not Applicable

P2SEXPC P2's sex partners: Transgender men
1

0 = No
1 = Yes
7 = Don't Know
8 = Refuse to Answer
9 = Not Applicable

P2SEXPD P2's sex partners: Transgender women
1

0 = No
1 = Yes
7 = Don't Know
8 = Refuse to Answer
9 = Not Applicable

P2SEXPE P2's sex partners: Gender non-conforming or gender non-binary
1

0 = No
1 = Yes
7 = Don't Know
8 = Refuse to Answer
9 = Not Applicable

P2SEXPF P2's sex partners: None
1

0 = No
1 = Yes
7 = Don't Know
8 = Refuse to Answer
9 = Not Applicable

Q372. Where did you first meet [Response to Q362]? [Do NOT read choices. Check ONE.]

P2MET P2 where met
2

1 = Bar
2 = Cafe or restaurant
3 = Dance club
4 = Adult book/video store, retail, business
5 = Sex club
6 = Street, park, library, public, transportation
7 = Church, political function, social club
8 = Work or school
9 = Gym or athletic activity
10 = Online - Dating app (OKCupid, Tinder, etc.)
11 = Online - Craigslist
12 = Online - Other, please specify
13 = Dating services or newspaper
14 = Private party or social club
15 = Introduce by friends
16 = Some other way.
97 = Don't Know
98 = Refuse to Answer
99 = Not Applicable

Q373. Please specify on where online you have met [Response to Q362]:

P2METOSP P2 met online specify
50

0 - 50 = range
97 = Don't Know
98 = Refuse to Answer
99 = Not Applicable

Q374. Please specify on where you have met [Response to Q362]:

P2METSP P2 met specify
50

0 - 50 = range
97 = Don't Know

98 = Refuse to Answer
99 = Not Applicable

Q375. To the best of your knowledge what is [Response to Q362]'s HIV status?

P2SERO P2 sero status
1

1 = Negative
2 = Positive
3 = Unknown
7 = Don't Know
8 = Refuse to Answer
9 = Not Applicable

Q376. To your knowledge, was this partner taking PrEP? (anti-HIV medications)

P2PREP P2 taking prep
1

0 = No
1 = Yes
7 = Don't Know
8 = Refuse to Answer
9 = Not Applicable

Q377. To your knowledge, was this partner on antiretrovirals?

P2ART P2 on ART
1

0 = No
1 = Yes
7 = Don't Know
8 = Refuse to Answer
9 = Not Applicable

Q378. To your knowledge, was this partner virally suppressed or their viral load undetectable?

P2VIRSUP P2 virally suppressed
1

0 = No
1 = Yes
7 = Don't Know
8 = Refuse to Answer
9 = Not Applicable

Q379. Did you disclose your HIV status to this partner before having sex the first time?

P2DISCL Disclosed HIV status to P2
1

0 = No
1 = Yes
7 = Don't Know
8 = Refuse to Answer
9 = Not Applicable

Q380. Did you have vaginal sex with [Response to Q362] during the last 6 months?

P2VAG P2 vaginal sex
1

0 = No
1 = Yes
7 = Don't Know
8 = Refuse to Answer
9 = Not Applicable

Q381. How many times have you had insertive vaginal intercourse with [Response to Q362] in the past six months?

P2VAGI P2 # vaginal insertive sex
3

0 - 996 = range
997 = Don't Know
998 = Refuse to Answer
999 = Not Applicable

Q382. Of the [Response to Q381] times you had insertive vaginal sex with this partner,

how many times did you NOT use a condom?

P2VAGIC P2 # vag ins no condom
3

0 - 996 = range
997 = Don't Know
998 = Refuse to Answer
999 = Not Applicable

Q383. Of the [Response to Q382] times you had insertive vaginal sex without a condom,

how many times were you high or drunk?

P2VAGIH P2 # vag ins unprot sex high or drunk
3

0 - 996 = range
997 = Don't Know
998 = Refuse to Answer
999 = Not Applicable

Q384. Now think of the last time you had insertive vaginal sex with [Response to Q362], did you use a condom?

P2VAGICL P2 last ins vag no condom
1

0 = No
1 = Yes
7 = Don't Know
8 = Refuse to Answer
9 = Not Applicable

Q385. Were you drinking or using drugs in the 2 hours before or during the last time you had insertive vaginal sex?

P2VAGIHL P2 last vag ins sex high or drunk
1

0 = No
1 = Yes
7 = Don't Know
8 = Refuse to Answer
9 = Not Applicable

Q386. What substances were you using?

P2VISUB P2 last vag ins what substances 2

0 - 18 = range
97 = Don't Know
98 = Refuse to Answer
99 = Not Applicable

P2VISUBA P2 last vag ins what substances: Alcohol 1

0 = No
1 = Yes
7 = Don't Know
8 = Refuse to Answer
9 = Not Applicable

P2VISUBB P2 last vag ins what substances: Meth 1

0 = No
1 = Yes
7 = Don't Know
8 = Refuse to Answer
9 = Not Applicable

P2VISUBC P2 last vag ins what substances: Crack 1

0 = No
1 = Yes
7 = Don't Know
8 = Refuse to Answer
9 = Not Applicable

P2VISUBD P2 last vag ins what substances: Cocaine 1

0 = No
1 = Yes
7 = Don't Know
8 = Refuse to Answer
9 = Not Applicable

P2VISUBE P2 last vag ins what substances: Poppers 1

0 = No
1 = Yes
7 = Don't Know
8 = Refuse to Answer
9 = Not Applicable

P2VISUBF P2 last vag ins what substances: GHB 1

0 = No
1 = Yes
7 = Don't Know

8 = Refuse to Answer
9 = Not Applicable

P2VISUBG P2 last vag ins what substances: Heroin
1

0 = No
1 = Yes
7 = Don't Know
8 = Refuse to Answer
9 = Not Applicable

P2VISUBH P2 last vag ins what substances: Marijuana
1

0 = No
1 = Yes
7 = Don't Know
8 = Refuse to Answer
9 = Not Applicable

P2VISUBI P2 last vag ins what substances: Synthetic cannabinoids
1

0 = No
1 = Yes
7 = Don't Know
8 = Refuse to Answer
9 = Not Applicable

P2VISUBJ P2 last vag ins what substances: Hallucinogens
1

0 = No
1 = Yes
7 = Don't Know
8 = Refuse to Answer
9 = Not Applicable

P2VISUBK P2 last vag ins what substances: Ecstasy
1

0 = No
1 = Yes
7 = Don't Know
8 = Refuse to Answer
9 = Not Applicable

P2VISUBL P2 last vag ins what substances: Rohypnol (roofies)
1

0 = No
1 = Yes
7 = Don't Know
8 = Refuse to Answer
9 = Not Applicable

P2VISUBM P2 last vag ins what substances: Ketamine
1

0 = No

1 = Yes
7 = Don't Know
8 = Refuse to Answer
9 = Not Applicable

P2VISUBN P2 last vag ins what substances: Benzos or barbituates
1

0 = No
1 = Yes
7 = Don't Know
8 = Refuse to Answer
9 = Not Applicable

P2VISUBO P2 last vag ins what substances: Painkillers
1

0 = No
1 = Yes
7 = Don't Know
8 = Refuse to Answer
9 = Not Applicable

P2VISUBP P2 last vag ins what substances: Viagra
1

0 = No
1 = Yes
7 = Don't Know
8 = Refuse to Answer
9 = Not Applicable

P2VISUBQ P2 last vag ins what substances: Prescription drugs
1

0 = No
1 = Yes
7 = Don't Know
8 = Refuse to Answer
9 = Not Applicable

P2VISUBR P2 last vag ins what substances: Other
1

0 = No
1 = Yes
7 = Don't Know
8 = Refuse to Answer
9 = Not Applicable

Q387. How many times have you had receptive vaginal intercourse with [Response to Q362] in the past six months?

P2VAGR P2 # vaginal receptive sex
3

0 - 996 = range
997 = Don't Know
998 = Refuse to Answer
999 = Not Applicable

Q388. Of the [Response to Q387] times you had receptive vaginal sex, how many times did you NOT use a condom?

P2VAGRC P2 # vag rec no condom

3

0 - 996 = range
997 = Don't Know
998 = Refuse to Answer
999 = Not Applicable

Q389. Of the [Response to Q388] times you had receptive vaginal sex without a condom, how many times were you high or drunk?

P2VAGRH P2 # vag rec unprot sex high or drunk

3

0 - 996 = range
997 = Don't Know
998 = Refuse to Answer
999 = Not Applicable

Q390. Now think of the last time you had receptive vaginal sex with [Response to Q362], did you use a condom?

P2VAGRCL P2 last rec vag no condom

1

0 = No
1 = Yes
7 = Don't Know
8 = Refuse to Answer
9 = Not Applicable

Q391. Were you drinking or using drugs in the 2 hours before or during the last time you had receptive vaginal sex?

P2VAGRHL P2 last vag rec sex high or drunk

1

0 = No
1 = Yes
7 = Don't Know
8 = Refuse to Answer
9 = Not Applicable

Q392. What substances were you using?

P2VRSUB P2 last vag rec what substances

2

0 - 18 = range
97 = Don't Know
98 = Refuse to Answer
99 = Not Applicable

P2VRSUBA P2 last vag rec what substances: Alcohol

1

0 = No
1 = Yes
7 = Don't Know
8 = Refuse to Answer

9 = Not Applicable

P2VRSUBB P2 last vag rec what substances: Meth
1

0 = No
1 = Yes
7 = Don't Know
8 = Refuse to Answer
9 = Not Applicable

P2VRSUBC P2 last vag rec what substances: Crack
1

0 = No
1 = Yes
7 = Don't Know
8 = Refuse to Answer
9 = Not Applicable

P2VRSUBD P2 last vag rec what substances: Cocaine
1

0 = No
1 = Yes
7 = Don't Know
8 = Refuse to Answer
9 = Not Applicable

P2VRSUBE P2 last vag rec what substances: Poppers
1

0 = No
1 = Yes
7 = Don't Know
8 = Refuse to Answer
9 = Not Applicable

P2VRSUBF P2 last vag rec what substances: GHB
1

0 = No
1 = Yes
7 = Don't Know
8 = Refuse to Answer
9 = Not Applicable

P2VRSUBG P2 last vag rec what substances: Heroin
1

0 = No
1 = Yes
7 = Don't Know
8 = Refuse to Answer
9 = Not Applicable

P2VRSUBH P2 last vag rec what substances: Marijuana
1

0 = No
1 = Yes

7 = Don't Know
8 = Refuse to Answer
9 = Not Applicable

P2VRSUBI P2 last vag rec what substances: Synthetic cannabinoids
1

0 = No
1 = Yes
7 = Don't Know
8 = Refuse to Answer
9 = Not Applicable

P2VRSUBJ P2 last vag rec what substances: Hallucinogens
1

0 = No
1 = Yes
7 = Don't Know
8 = Refuse to Answer
9 = Not Applicable

P2VRSUBK P2 last vag rec what substances: Ecstasy
1

0 = No
1 = Yes
7 = Don't Know
8 = Refuse to Answer
9 = Not Applicable

P2VRSUBL P2 last vag rec what substances: Rohypnol (roofies)
1

0 = No
1 = Yes
7 = Don't Know
8 = Refuse to Answer
9 = Not Applicable

P2VRSUBM P2 last vag rec what substances: Ketamine
1

0 = No
1 = Yes
7 = Don't Know
8 = Refuse to Answer
9 = Not Applicable

P2VRSUBN P2 last vag rec what substances: Benzos or barbituates
1

0 = No
1 = Yes
7 = Don't Know
8 = Refuse to Answer
9 = Not Applicable

P2VRSUBO P2 last vag rec what substances: Painkillers
1

0 = No
1 = Yes
7 = Don't Know
8 = Refuse to Answer
9 = Not Applicable

P2VRSUBP P2 last vag rec what substances: Viagra
1

0 = No
1 = Yes
7 = Don't Know
8 = Refuse to Answer
9 = Not Applicable

P2VRSUBQ P2 last vag rec what substances: Prescription drugs
1

0 = No
1 = Yes
7 = Don't Know
8 = Refuse to Answer
9 = Not Applicable

P2VRSUBR P2 last vag rec what substances: Other
1

0 = No
1 = Yes
7 = Don't Know
8 = Refuse to Answer
9 = Not Applicable

Q393. Did you have anal sex with [Response to Q362] during the last 6 months?

P2ANAL P2 anal sex
1

0 = No
1 = Yes
7 = Don't Know
8 = Refuse to Answer
9 = Not Applicable

Q394. How many times have you had insertive anal intercourse with [Response to Q362] in the past 6 months?

P2ANAI P2 # insertive anal sex
3

0 - 996 = range
997 = Don't Know
998 = Refuse to Answer
999 = Not Applicable

Q395. Of the [Response to Q394] times you had insertive anal sex,

how many times did you NOT use a condom?

P2ANAIC P2 # anal ins no condom
3

0 - 996 = range
997 = Don't Know
998 = Refuse to Answer
999 = Not Applicable

Q396. Of the [Response to Q395] times you had insertive anal sex without a condom, how many times were you high or drunk?

P2ANAIH P2 # anal ins unprot sex high or drunk
3

0 - 996 = range
997 = Don't Know
998 = Refuse to Answer
999 = Not Applicable

Q397. Now think of the last time you had insertive anal sex with [Response to Q362], did you use a condom?

P2ANAICL P2 last ins anal no condom
1

0 = No
1 = Yes
7 = Don't Know
8 = Refuse to Answer
9 = Not Applicable

Q398. Were you drinking or using drugs in the 2 hours before or during the last time you had insertive anal sex?

P2ANAIHL P2 last anal ins sex high or drunk
1

0 = No
1 = Yes
7 = Don't Know
8 = Refuse to Answer
9 = Not Applicable

Q399. What substances were you using?

P2AISUB P2 last anal ins what substances
2

0 - 18 = range
97 = Don't Know
98 = Refuse to Answer
99 = Not Applicable

P2AISUBA P2 last anal ins what substances: Alcohol
1

0 = No
1 = Yes
7 = Don't Know
8 = Refuse to Answer
9 = Not Applicable

P2AISUBB P2 last anal ins what substances: Meth
1

0 = No

1 = Yes
7 = Don't Know
8 = Refuse to Answer
9 = Not Applicable

P2AISUBC P2 last anal ins what substances: Crack
1

0 = No
1 = Yes
7 = Don't Know
8 = Refuse to Answer
9 = Not Applicable

P2AISUBD P2 last anal ins what substances: Cocaine
1

0 = No
1 = Yes
7 = Don't Know
8 = Refuse to Answer
9 = Not Applicable

P2AISUBE P2 last anal ins what substances: Poppers
1

0 = No
1 = Yes
7 = Don't Know
8 = Refuse to Answer
9 = Not Applicable

P2AISUBF P2 last anal ins what substances: GHB
1

0 = No
1 = Yes
7 = Don't Know
8 = Refuse to Answer
9 = Not Applicable

P2AISUBG P2 last anal ins what substances: Heroin
1

0 = No
1 = Yes
7 = Don't Know
8 = Refuse to Answer
9 = Not Applicable

P2AISUBH P2 last anal ins what substances: Marijuana
1

0 = No
1 = Yes
7 = Don't Know
8 = Refuse to Answer
9 = Not Applicable

P2AISUBI P2 last anal ins what substances: Synthetic cannabinoids

1

0 = No
1 = Yes
7 = Don't Know
8 = Refuse to Answer
9 = Not Applicable

P2AISUBJ P2 last anal ins what substances: Hallucinogens

1

0 = No
1 = Yes
7 = Don't Know
8 = Refuse to Answer
9 = Not Applicable

P2AISUBK P2 last anal ins what substances: Ecstasy

1

0 = No
1 = Yes
7 = Don't Know
8 = Refuse to Answer
9 = Not Applicable

P2AISUBL P2 last anal ins what substances: Rohypnol (roofies)

1

0 = No
1 = Yes
7 = Don't Know
8 = Refuse to Answer
9 = Not Applicable

P2AISUBM P2 last anal ins what substances: Ketamine

1

0 = No
1 = Yes
7 = Don't Know
8 = Refuse to Answer
9 = Not Applicable

P2AISUBN P2 last anal ins what substances: Benzos or barbituates

1

0 = No
1 = Yes
7 = Don't Know
8 = Refuse to Answer
9 = Not Applicable

P2AISUBO P2 last anal ins what substances: Painkillers

1

0 = No
1 = Yes
7 = Don't Know
8 = Refuse to Answer
9 = Not Applicable

P2AISUBP P2 last anal ins what substances: Viagra
1

0 = No
1 = Yes
7 = Don't Know
8 = Refuse to Answer
9 = Not Applicable

P2AISUBQ P2 last anal ins what substances: Prescription drugs
1

0 = No
1 = Yes
7 = Don't Know
8 = Refuse to Answer
9 = Not Applicable

P2AISUBR P2 last anal ins what substances: Other
1

0 = No
1 = Yes
7 = Don't Know
8 = Refuse to Answer
9 = Not Applicable

Q400. How many times have you had receptive anal intercourse with [Response to Q362] in the past six months.

P2ANAR P2 # receptive anal sex
3

0 - 996 = range
997 = Don't Know
998 = Refuse to Answer
999 = Not Applicable

Q401. Of the [Response to Q400] times you had receptive anal sex,

how many times did you NOT use a condom?

P2ANARC P2 # anal rec no condom
3

0 - 996 = range
997 = Don't Know
998 = Refuse to Answer
999 = Not Applicable

Q402. Of the [Response to Q401] times you had receptive anal sex without a condom,

how many times were you high or drunk?

P2ANARH P2 # anal rec unprot sex high or drunk
3

0 - 996 = range
997 = Don't Know
998 = Refuse to Answer
999 = Not Applicable

Q403. Now think of the last time you had receptive anal sex with [Response to Q362], did you use a condom?

P2ANARCL P2 last rec anal no condom
1

0 = No
1 = Yes
7 = Don't Know
8 = Refuse to Answer
9 = Not Applicable

Q404. Were you drinking or using drugs in the 2 hours before or during the last time you had receptive anal sex?

P2ANARHL P2 last anal rec sex high or drunk
1

0 = No
1 = Yes
7 = Don't Know
8 = Refuse to Answer
9 = Not Applicable

Q405. What substances were you using?

P2ARSUB P2 last anal rec what substances
2

0 - 18 = range
97 = Don't Know
98 = Refuse to Answer
99 = Not Applicable

P2ARSUBA P2 last anal rec what substances: Alcohol
1

0 = No
1 = Yes
7 = Don't Know
8 = Refuse to Answer
9 = Not Applicable

P2ARSUBB P2 last anal rec what substances: Meth
1

0 = No
1 = Yes
7 = Don't Know
8 = Refuse to Answer
9 = Not Applicable

P2ARSUBC P2 last anal rec what substances: Crack
1

0 = No
1 = Yes
7 = Don't Know
8 = Refuse to Answer
9 = Not Applicable

P2ARSUBD P2 last anal rec what substances: Cocaine

1

0 = No
1 = Yes
7 = Don't Know
8 = Refuse to Answer
9 = Not Applicable

P2ARSUBE P2 last anal rec what substances: Poppers
1

0 = No
1 = Yes
7 = Don't Know
8 = Refuse to Answer
9 = Not Applicable

P2ARSUBF P2 last anal rec what substances: GHB
1

0 = No
1 = Yes
7 = Don't Know
8 = Refuse to Answer
9 = Not Applicable

P2ARSUBG P2 last anal rec what substances: Heroin
1

0 = No
1 = Yes
7 = Don't Know
8 = Refuse to Answer
9 = Not Applicable

P2ARSUBH P2 last anal rec what substances: Marijuana
1

0 = No
1 = Yes
7 = Don't Know
8 = Refuse to Answer
9 = Not Applicable

P2ARSUBI P2 last anal rec what substances: Synthetic cannabinoids
1

0 = No
1 = Yes
7 = Don't Know
8 = Refuse to Answer
9 = Not Applicable

P2ARSUBJ P2 last anal rec what substances: Hallucinogens
1

0 = No
1 = Yes
7 = Don't Know
8 = Refuse to Answer
9 = Not Applicable

P2ARSUBK P2 last anal rec what substances: Ecstasy
1

0 = No
1 = Yes
7 = Don't Know
8 = Refuse to Answer
9 = Not Applicable

P2ARSUBL P2 last anal rec what substances: Rohypnol (roofies)
1

0 = No
1 = Yes
7 = Don't Know
8 = Refuse to Answer
9 = Not Applicable

P2ARSUBM P2 last anal rec what substances: Ketamine
1

0 = No
1 = Yes
7 = Don't Know
8 = Refuse to Answer
9 = Not Applicable

P2ARSUBN P2 last anal rec what substances: Benzos or barbituates
1

0 = No
1 = Yes
7 = Don't Know
8 = Refuse to Answer
9 = Not Applicable

P2ARSUBO P2 last anal rec what substances: Painkillers
1

0 = No
1 = Yes
7 = Don't Know
8 = Refuse to Answer
9 = Not Applicable

P2ARSUBP P2 last anal rec what substances: Viagra
1

0 = No
1 = Yes
7 = Don't Know
8 = Refuse to Answer
9 = Not Applicable

P2ARSUBQ P2 last anal rec what substances: Prescription drugs
1

0 = No
1 = Yes
7 = Don't Know

8 = Refuse to Answer
9 = Not Applicable

P2ARSUBR P2 last anal rec what substances: Other
1

0 = No
1 = Yes
7 = Don't Know
8 = Refuse to Answer
9 = Not Applicable

Q406. Does this partner inject drugs?

P2INJ P2 inject drugs
1

0 = No
1 = Yes
7 = Don't Know
8 = Refuse to Answer
9 = Not Applicable

Q407. To help guide you through the questions, please provide initials for this next person: we will refer to this person later using the initials you provide.

P3IN P3 Initials
20

0 - 20 = range
97 = Don't Know
98 = Refuse to Answer
99 = Not Applicable

Q408. What is [Response to Q407]s gender? [Do NOT read choices. Check ONE.]

P3GEN P3 gender
2

1 = Cisgender male
2 = Cisgender female
3 = Transgender female / transwoman (Male to female transgender)
4 = Transgender male/transman (Female to male transgender)
5 = Androgynous/ambigender
6 = Questioning
7 = Genderqueer/genderfluid
8 = Other
97 = Don't Know
98 = Refuse to Answer
99 = Not Applicable

Q409. Did [Response to Q407] have a penis?

P3PENIS P3 has penis
1

0 = No
1 = Yes
7 = Don't Know

8 = Refuse to Answer
9 = Not Applicable

Q410. How old was [Response to Q407]?

P3AGE P3 Age
2

0 - 96 = range
97 = Don't Know
98 = Refuse to Answer
99 = Not Applicable

Q411. What type of partner was [Response to Q407]?

Main Partner: Someone who is your primary sexual partner and you feel committed to (boyfriend, lover, husband, girlfriend, wife).

Casual Partner: Someone you have sex with, but don't feel committed to or don't know very well.

Exchange partner/Trick: Someone you had sex with in exchange for things like money, goods, or a place to stay.

P3TYPE P3 type
1

1 = Main partner
2 = Casual partner
3 = Exchange partner / Trick
4 = Sexual assault
7 = Don't Know
8 = Refuse to Answer
9 = Not Applicable

Q412. Did this partner pay you or did you pay this partner?

P3PAYOR P3 paid/you paid partner
1

1 = I was paid
2 = I paid this partner
7 = Don't Know
8 = Refuse to Answer
9 = Not Applicable

Q413. Would you consider this partner to be trade?

P3TRADE P3 considered trade
1

0 = No
1 = Yes
7 = Don't Know
8 = Refuse to Answer
9 = Not Applicable

Q414. What is [Response to Q407]'s race or ethnicity? Would you describe [Response to Q407] as:

[Do NOT read choices. Check ALL that apply]

P3RACE P3 race
2

0 - 7 = range
97 = Don't Know

98 = Refuse to Answer
99 = Not Applicable

P3RACEA P3 race: Asian
1

0 = No
1 = Yes
7 = Don't Know
8 = Refuse to Answer
9 = Not Applicable

P3RACEB P3 race: Black / African American
1

0 = No
1 = Yes
7 = Don't Know
8 = Refuse to Answer
9 = Not Applicable

P3RACEC P3 race: Native American
1

0 = No
1 = Yes
7 = Don't Know
8 = Refuse to Answer
9 = Not Applicable

P3RACED P3 race: Native Hawaiian or Pacific Islander
1

0 = No
1 = Yes
7 = Don't Know
8 = Refuse to Answer
9 = Not Applicable

P3RACEE P3 race: White
1

0 = No
1 = Yes
7 = Don't Know
8 = Refuse to Answer
9 = Not Applicable

P3RACEF P3 race: Latino/Latina
1

0 = No
1 = Yes
7 = Don't Know
8 = Refuse to Answer
9 = Not Applicable

P3RACEG P3 race: Other
1

0 = No
1 = Yes

7 = Don't Know
8 = Refuse to Answer
9 = Not Applicable

Q415. Please specify race / ethnicity:

P3RACESP P3 other race 20

0 - 20 = range
97 = Don't Know
98 = Refuse to Answer
99 = Not Applicable

Q416. To your knowledge, in the past 6 months has [Response to Q407] had sex with other people who are:

[READ choices. Check ALL that apply.]

P3SEXP P3's sex partners 1

0 - 6 = range
7 = Don't Know
8 = Refuse to Answer
9 = Not Applicable

P3SEXPA P3's sex partners: Cisgender men 1

0 = No
1 = Yes
7 = Don't Know
8 = Refuse to Answer
9 = Not Applicable

P3SEXPB P3's sex partners: Cisgender women 1

0 = No
1 = Yes
7 = Don't Know
8 = Refuse to Answer
9 = Not Applicable

P3SEXPC P3's sex partners: Transgender men 1

0 = No
1 = Yes
7 = Don't Know
8 = Refuse to Answer
9 = Not Applicable

P3SEXPD P3's sex partners: Transgender women 1

0 = No
1 = Yes
7 = Don't Know
8 = Refuse to Answer
9 = Not Applicable

P3SEXPE P3's sex partners: Gender non-conforming or gender non-binary
1

0 = No
1 = Yes
7 = Don't Know
8 = Refuse to Answer
9 = Not Applicable

P3SEXPF P3's sex partners: None
1

0 = No
1 = Yes
7 = Don't Know
8 = Refuse to Answer
9 = Not Applicable

Q417. Where did you first meet [Response to Q407]? [Do NOT read choices. Check ONE.]

P3MET P3 where met
2

1 = Bar
2 = Cafe or restaurant
3 = Dance club
4 = Adult book/video store, retail, business
5 = Sex club
6 = Street, park, library, public, transportation
7 = Church, political function, social club
8 = Work or school
9 = Gym or athletic activity
10 = Online - Dating app (OKCupid, Tinder, etc.)
11 = Online - Craigslist
12 = Online - Other, please specify
13 = Dating services or newspaper
14 = Private party or social club
15 = Introduce by friends
16 = Some other way.
97 = Don't Know
98 = Refuse to Answer
99 = Not Applicable

Q418. Please specify on where online you have met [Response to Q407]:

P3METOSP P3 met online specify
50

0 - 50 = range
97 = Don't Know
98 = Refuse to Answer
99 = Not Applicable

Q419. Please specify on where you have met [Response to Q407]:

P3METSP P3 met specify
50

0 - 50 = range

97 = Don't Know
98 = Refuse to Answer
99 = Not Applicable

Q420. To the best of your knowledge what is [Response to Q407]'s HIV status?

P3SERO P3 sero status
1

1 = Negative
2 = Positive
3 = Unknown
7 = Don't Know
8 = Refuse to Answer
9 = Not Applicable

Q421. To your knowledge, was this partner taking PrEP? (anti-HIV medications)

P3PREP P3 taking prep
1

0 = No
1 = Yes
7 = Don't Know
8 = Refuse to Answer
9 = Not Applicable

Q422. To your knowledge, was this partner on antiretrovirals?

P3ART P3 on ART
1

0 = No
1 = Yes
7 = Don't Know
8 = Refuse to Answer
9 = Not Applicable

Q423. To your knowledge, was this partner virally suppressed or their viral load undetectable?

P3VIRSUP P3 virally suppressed
1

0 = No
1 = Yes
7 = Don't Know
8 = Refuse to Answer
9 = Not Applicable

Q424. Did you disclose your HIV status to this partner before having sex the first time?

P3DISCL Disclosed HIV status to P3
1

0 = No
1 = Yes
7 = Don't Know
8 = Refuse to Answer
9 = Not Applicable

Q425. Did you have vaginal sex with [Response to Q407] during the last 6 months?

P3VAG P3 vaginal sex

1

0 = No
1 = Yes
7 = Don't Know
8 = Refuse to Answer
9 = Not Applicable

Q426. How many times have you had insertive vaginal intercourse with [Response to Q407] in the past six months?

P3VAGI P3 # vaginal insertive sex
3

0 - 996 = range
997 = Don't Know
998 = Refuse to Answer
999 = Not Applicable

Q427. Of the [Response to Q426] times you had insertive vaginal sex with this partner,

how many times did you NOT use a condom?

P3VAGIC P3 # vag ins no condom
3

0 - 996 = range
997 = Don't Know
998 = Refuse to Answer
999 = Not Applicable

Q428. Of the [Response to Q427] times you had insertive vaginal sex without a condom,

how many times were you high or drunk?

P3VAGIH P3 # vag ins unprot sex high or drunk
3

0 - 996 = range
997 = Don't Know
998 = Refuse to Answer
999 = Not Applicable

Q429. Now think of the last time you had insertive vaginal sex with [Response to Q407], did you use a condom?

P3VAGICL P3 last ins vag no condom
1

0 = No
1 = Yes
7 = Don't Know
8 = Refuse to Answer
9 = Not Applicable

Q430. Were you drinking or using drugs in the 2 hours before or during the last time you had insertive vaginal sex?

P3VAGIHL P3 last vag ins sex high or drunk
1

0 = No
1 = Yes
7 = Don't Know
8 = Refuse to Answer

9 = Not Applicable

Q431. What substances were you using?

P3VISUB P3 last vag ins what substances
2

0 - 18 = range
97 = Don't Know
98 = Refuse to Answer
99 = Not Applicable

P3VISUBA P3 last vag ins what substances: Alcohol
1

0 = No
1 = Yes
7 = Don't Know
8 = Refuse to Answer
9 = Not Applicable

P3VISUBB P3 last vag ins what substances: Meth
1

0 = No
1 = Yes
7 = Don't Know
8 = Refuse to Answer
9 = Not Applicable

P3VISUBC P3 last vag ins what substances: Crack
1

0 = No
1 = Yes
7 = Don't Know
8 = Refuse to Answer
9 = Not Applicable

P3VISUBD P3 last vag ins what substances: Cocaine
1

0 = No
1 = Yes
7 = Don't Know
8 = Refuse to Answer
9 = Not Applicable

P3VISUBE P3 last vag ins what substances: Poppers
1

0 = No
1 = Yes
7 = Don't Know
8 = Refuse to Answer
9 = Not Applicable

P3VISUBF P3 last vag ins what substances: GHB
1

0 = No
1 = Yes

7 = Don't Know
8 = Refuse to Answer
9 = Not Applicable

P3VISUBG P3 last vag ins what substances: Heroin
1

0 = No
1 = Yes
7 = Don't Know
8 = Refuse to Answer
9 = Not Applicable

P3VISUBH P3 last vag ins what substances: Marijuana
1

0 = No
1 = Yes
7 = Don't Know
8 = Refuse to Answer
9 = Not Applicable

P3VISUBI P3 last vag ins what substances: Synthetic cannabinoids
1

0 = No
1 = Yes
7 = Don't Know
8 = Refuse to Answer
9 = Not Applicable

P3VISUBJ P3 last vag ins what substances: Hallucinogens
1

0 = No
1 = Yes
7 = Don't Know
8 = Refuse to Answer
9 = Not Applicable

P3VISUBK P3 last vag ins what substances: Ecstasy
1

0 = No
1 = Yes
7 = Don't Know
8 = Refuse to Answer
9 = Not Applicable

P3VISUBL P3 last vag ins what substances: Rohypnol (roofies)
1

0 = No
1 = Yes
7 = Don't Know
8 = Refuse to Answer
9 = Not Applicable

P3VISUBM P3 last vag ins what substances: Ketamine
1

0 = No
1 = Yes
7 = Don't Know
8 = Refuse to Answer
9 = Not Applicable

P3VISUBN P3 last vag ins what substances: Benzos or barbituates
1

0 = No
1 = Yes
7 = Don't Know
8 = Refuse to Answer
9 = Not Applicable

P3VISUBO P3 last vag ins what substances: Painkillers
1

0 = No
1 = Yes
7 = Don't Know
8 = Refuse to Answer
9 = Not Applicable

P3VISUBP P3 last vag ins what substances: Viagra
1

0 = No
1 = Yes
7 = Don't Know
8 = Refuse to Answer
9 = Not Applicable

P3VISUBQ P3 last vag ins what substances: Prescription drugs
1

0 = No
1 = Yes
7 = Don't Know
8 = Refuse to Answer
9 = Not Applicable

P3VISUBR P3 last vag ins what substances: Other
1

0 = No
1 = Yes
7 = Don't Know
8 = Refuse to Answer
9 = Not Applicable

Q432. How many times have you had receptive vaginal intercourse with [Response to Q407] in the past six months?

P3VAGR P3 # vaginal receptive sex
3

0 - 996 = range
997 = Don't Know
998 = Refuse to Answer

999 = Not Applicable

Q433. Of the [Response to Q432] times you had receptive vaginal sex,
how many times did you NOT use a condom?

P3VAGRC P3 # vag rec no condom
3

0 - 996 = range
997 = Don't Know
998 = Refuse to Answer
999 = Not Applicable

Q434. Of the [Response to Q433] times you had receptive vaginal sex without a condom,
how many times were you high or drunk?

P3VAGRH P3 # vag rec unprot sex high or drunk
3

0 - 996 = range
997 = Don't Know
998 = Refuse to Answer
999 = Not Applicable

Q435. Now think of the last time you had receptive vaginal sex with [Response to Q407], did you use a condom?

P3VAGRCL P3 last rec vag no condom
1

0 = No
1 = Yes
7 = Don't Know
8 = Refuse to Answer
9 = Not Applicable

Q436. Were you drinking or using drugs in the 2 hours before or during the last time you had receptive vaginal sex?

P3VAGRHL P3 last vag rec sex high or drunk
1

0 = No
1 = Yes
7 = Don't Know
8 = Refuse to Answer
9 = Not Applicable

Q437. What substances were you using?

P3VRSUB P3 last vag rec what substances
2

0 - 18 = range
97 = Don't Know
98 = Refuse to Answer
99 = Not Applicable

P3VRSUBA P3 last vag rec what substances: Alcohol
1

0 = No
1 = Yes
7 = Don't Know

8 = Refuse to Answer
9 = Not Applicable

P3VRSUBB P3 last vag rec what substances: Meth 1

0 = No
1 = Yes
7 = Don't Know
8 = Refuse to Answer
9 = Not Applicable

P3VRSUBC P3 last vag rec what substances: Crack 1

0 = No
1 = Yes
7 = Don't Know
8 = Refuse to Answer
9 = Not Applicable

P3VRSUBD P3 last vag rec what substances: Cocaine 1

0 = No
1 = Yes
7 = Don't Know
8 = Refuse to Answer
9 = Not Applicable

P3VRSUBE P3 last vag rec what substances: Poppers 1

0 = No
1 = Yes
7 = Don't Know
8 = Refuse to Answer
9 = Not Applicable

P3VRSUBF P3 last vag rec what substances: GHB 1

0 = No
1 = Yes
7 = Don't Know
8 = Refuse to Answer
9 = Not Applicable

P3VRSUBG P3 last vag rec what substances: Heroin 1

0 = No
1 = Yes
7 = Don't Know
8 = Refuse to Answer
9 = Not Applicable

P3VRSUBH P3 last vag rec what substances: Marijuana 1

0 = No

1 = Yes
7 = Don't Know
8 = Refuse to Answer
9 = Not Applicable

P3VRSUBI P3 last vag rec what substances: Synthetic cannabinoids
1

0 = No
1 = Yes
7 = Don't Know
8 = Refuse to Answer
9 = Not Applicable

P3VRSUBJ P3 last vag rec what substances: Hallucinogens
1

0 = No
1 = Yes
7 = Don't Know
8 = Refuse to Answer
9 = Not Applicable

P3VRSUBK P3 last vag rec what substances: Ecstasy
1

0 = No
1 = Yes
7 = Don't Know
8 = Refuse to Answer
9 = Not Applicable

P3VRSUBL P3 last vag rec what substances: Rohypnol (roofies)
1

0 = No
1 = Yes
7 = Don't Know
8 = Refuse to Answer
9 = Not Applicable

P3VRSUBM P3 last vag rec what substances: Ketamine
1

0 = No
1 = Yes
7 = Don't Know
8 = Refuse to Answer
9 = Not Applicable

P3VRSUBN P3 last vag rec what substances: Benzos or barbituates
1

0 = No
1 = Yes
7 = Don't Know
8 = Refuse to Answer
9 = Not Applicable

P3VRSUBO P3 last vag rec what substances: Painkillers

1

0 = No
1 = Yes
7 = Don't Know
8 = Refuse to Answer
9 = Not Applicable

P3VRSUBP P3 last vag rec what substances: Viagra
1

0 = No
1 = Yes
7 = Don't Know
8 = Refuse to Answer
9 = Not Applicable

P3VRSUBQ P3 last vag rec what substances: Prescription drugs
1

0 = No
1 = Yes
7 = Don't Know
8 = Refuse to Answer
9 = Not Applicable

P3VRSUBR P3 last vag rec what substances: Other
1

0 = No
1 = Yes
7 = Don't Know
8 = Refuse to Answer
9 = Not Applicable

Q438. Did you have anal sex with [Response to Q407] during the last 6 months?

P3ANAL P3 anal sex
1

0 = No
1 = Yes
7 = Don't Know
8 = Refuse to Answer
9 = Not Applicable

Q439. How many times have you had insertive anal intercourse with [Response to Q407] in the past 6 months?

P3ANAI P3 # insertive anal sex
3

0 - 996 = range
997 = Don't Know
998 = Refuse to Answer
999 = Not Applicable

Q440. Of the [Response to Q439] times you had insertive anal sex,

how many times did you NOT use a condom?

P3ANAIC P3 # anal ins no condom

0 - 996 = range
997 = Don't Know
998 = Refuse to Answer
999 = Not Applicable

Q441. Of the [Response to Q440] times you had insertive anal sex without a condom, how many times were you high or drunk?

P3ANAIH P3 # anal ins unprot sex high or drunk
3

0 - 996 = range
997 = Don't Know
998 = Refuse to Answer
999 = Not Applicable

Q442. Now think of the last time you had insertive anal sex with [Response to Q407], did you use a condom?

P3ANAICL P3 last ins anal no condom
1

0 = No
1 = Yes
7 = Don't Know
8 = Refuse to Answer
9 = Not Applicable

Q443. Were you drinking or using drugs in the 2 hours before or during the last time you had insertive anal sex?

P3ANAIHL P3 last anal ins sex high or drunk
1

0 = No
1 = Yes
7 = Don't Know
8 = Refuse to Answer
9 = Not Applicable

Q444. What substances were you using?

P3AISUB P3 last anal ins what substances
2

0 - 18 = range
97 = Don't Know
98 = Refuse to Answer
99 = Not Applicable

P3AISUBA P3 last anal ins what substances: Alcohol
1

0 = No
1 = Yes
7 = Don't Know
8 = Refuse to Answer
9 = Not Applicable

P3AISUBB P3 last anal ins what substances: Meth
1

0 = No
1 = Yes
7 = Don't Know
8 = Refuse to Answer
9 = Not Applicable

P3AISUBC P3 last anal ins what substances: Crack
1

0 = No
1 = Yes
7 = Don't Know
8 = Refuse to Answer
9 = Not Applicable

P3AISUBD P3 last anal ins what substances: Cocaine
1

0 = No
1 = Yes
7 = Don't Know
8 = Refuse to Answer
9 = Not Applicable

P3AISUBE P3 last anal ins what substances: Poppers
1

0 = No
1 = Yes
7 = Don't Know
8 = Refuse to Answer
9 = Not Applicable

P3AISUBF P3 last anal ins what substances: GHB
1

0 = No
1 = Yes
7 = Don't Know
8 = Refuse to Answer
9 = Not Applicable

P3AISUBG P3 last anal ins what substances: Heroin
1

0 = No
1 = Yes
7 = Don't Know
8 = Refuse to Answer
9 = Not Applicable

P3AISUBH P3 last anal ins what substances: Marijuana
1

0 = No
1 = Yes
7 = Don't Know
8 = Refuse to Answer
9 = Not Applicable

P3AISUBI P3 last anal ins what substances: Synthetic cannabinoids
1

0 = No
1 = Yes
7 = Don't Know
8 = Refuse to Answer
9 = Not Applicable

P3AISUBJ P3 last anal ins what substances: Hallucinogens
1

0 = No
1 = Yes
7 = Don't Know
8 = Refuse to Answer
9 = Not Applicable

P3AISUBK P3 last anal ins what substances: Ecstasy
1

0 = No
1 = Yes
7 = Don't Know
8 = Refuse to Answer
9 = Not Applicable

P3AISUBL P3 last anal ins what substances: Rohypnol (roofies)
1

0 = No
1 = Yes
7 = Don't Know
8 = Refuse to Answer
9 = Not Applicable

P3AISUBM P3 last anal ins what substances: Ketamine
1

0 = No
1 = Yes
7 = Don't Know
8 = Refuse to Answer
9 = Not Applicable

P3AISUBN P3 last anal ins what substances: Benzos or barbituates
1

0 = No
1 = Yes
7 = Don't Know
8 = Refuse to Answer
9 = Not Applicable

P3AISUBO P3 last anal ins what substances: Painkillers
1

0 = No
1 = Yes
7 = Don't Know
8 = Refuse to Answer

9 = Not Applicable

P3AISUBP P3 last anal ins what substances: Viagra
1

0 = No
1 = Yes
7 = Don't Know
8 = Refuse to Answer
9 = Not Applicable

P3AISUBQ P3 last anal ins what substances: Prescription drugs
1

0 = No
1 = Yes
7 = Don't Know
8 = Refuse to Answer
9 = Not Applicable

P3AISUBR P3 last anal ins what substances: Other
1

0 = No
1 = Yes
7 = Don't Know
8 = Refuse to Answer
9 = Not Applicable

Q445. How many times have you had receptive anal intercourse with [Response to Q407] in the past six months.

P3ANAR P3 # receptive anal sex
3

0 - 996 = range
997 = Don't Know
998 = Refuse to Answer
999 = Not Applicable

Q446. Of the [Response to Q445] times you had receptive anal sex,

how many times did you NOT use a condom?

P3ANARC P3 # anal rec no condom
3

0 - 996 = range
997 = Don't Know
998 = Refuse to Answer
999 = Not Applicable

Q447. Of the [Response to Q446] times you had receptive anal sex without a condom,

how many times were you high or drunk?

P3ANARH P3 # anal rec unprot sex high or drunk
3

0 - 996 = range
997 = Don't Know
998 = Refuse to Answer

999 = Not Applicable

Q448. Now think of the last time you had receptive anal sex with [Response to Q407], did you use a condom?

P3ANARCL P3 last rec anal no condom
1

0 = No
1 = Yes
7 = Don't Know
8 = Refuse to Answer
9 = Not Applicable

Q449. Were you drinking or using drugs in the 2 hours before or during the last time you had receptive anal sex?

P3ANARHL P3 last anal rec sex high or drunk
1

0 = No
1 = Yes
7 = Don't Know
8 = Refuse to Answer
9 = Not Applicable

Q450. What substances were you using?

P3ARSUB P3 last anal rec what substances
2

0 - 18 = range
97 = Don't Know
98 = Refuse to Answer
99 = Not Applicable

P3ARSUBA P3 last anal rec what substances: Alcohol
1

0 = No
1 = Yes
7 = Don't Know
8 = Refuse to Answer
9 = Not Applicable

P3ARSUBB P3 last anal rec what substances: Meth
1

0 = No
1 = Yes
7 = Don't Know
8 = Refuse to Answer
9 = Not Applicable

P3ARSUBC P3 last anal rec what substances: Crack
1

0 = No
1 = Yes
7 = Don't Know
8 = Refuse to Answer
9 = Not Applicable

P3ARSUBD P3 last anal rec what substances: Cocaine
1

0 = No
1 = Yes
7 = Don't Know
8 = Refuse to Answer
9 = Not Applicable

P3ARSUBE P3 last anal rec what substances: Poppers
1

0 = No
1 = Yes
7 = Don't Know
8 = Refuse to Answer
9 = Not Applicable

P3ARSUBF P3 last anal rec what substances: GHB
1

0 = No
1 = Yes
7 = Don't Know
8 = Refuse to Answer
9 = Not Applicable

P3ARSUBG P3 last anal rec what substances: Heroin
1

0 = No
1 = Yes
7 = Don't Know
8 = Refuse to Answer
9 = Not Applicable

P3ARSUBH P3 last anal rec what substances: Marijuana
1

0 = No
1 = Yes
7 = Don't Know
8 = Refuse to Answer
9 = Not Applicable

P3ARSUBI P3 last anal rec what substances: Synthetic cannabinoids
1

0 = No
1 = Yes
7 = Don't Know
8 = Refuse to Answer
9 = Not Applicable

P3ARSUBJ P3 last anal rec what substances: Hallucinogens
1

0 = No
1 = Yes
7 = Don't Know
8 = Refuse to Answer

9 = Not Applicable

P3ARSUBK P3 last anal rec what substances: Ecstasy
1

0 = No
1 = Yes
7 = Don't Know
8 = Refuse to Answer
9 = Not Applicable

P3ARSUBL P3 last anal rec what substances: Rohypnol (roofies)
1

0 = No
1 = Yes
7 = Don't Know
8 = Refuse to Answer
9 = Not Applicable

P3ARSUBM P3 last anal rec what substances: Ketamine
1

0 = No
1 = Yes
7 = Don't Know
8 = Refuse to Answer
9 = Not Applicable

P3ARSUBN P3 last anal rec what substances: Benzos or barbituates
1

0 = No
1 = Yes
7 = Don't Know
8 = Refuse to Answer
9 = Not Applicable

P3ARSUBO P3 last anal rec what substances: Painkillers
1

0 = No
1 = Yes
7 = Don't Know
8 = Refuse to Answer
9 = Not Applicable

P3ARSUBP P3 last anal rec what substances: Viagra
1

0 = No
1 = Yes
7 = Don't Know
8 = Refuse to Answer
9 = Not Applicable

P3ARSUBQ P3 last anal rec what substances: Prescription drugs
1

0 = No
1 = Yes

7 = Don't Know
8 = Refuse to Answer
9 = Not Applicable

P3ARSUBR P3 last anal rec what substances: Other
1

0 = No
1 = Yes
7 = Don't Know
8 = Refuse to Answer
9 = Not Applicable

Q451. Does this partner inject drugs?

P3INJ P3 inject drugs
1

0 = No
1 = Yes
7 = Don't Know
8 = Refuse to Answer
9 = Not Applicable

Q452. To help guide you through the questions, please provide initials for this next person: we will refer to this person later using the initials you provide.

P4IN P4 Initials
20

0 - 20 = range
97 = Don't Know
98 = Refuse to Answer
99 = Not Applicable

Q453. What is [Response to Q452]s gender? [Do NOT read choices. Check ONE.]

P4GEN P4 gender
2

1 = Cisgender male
2 = Cisgender female
3 = Transgender female / transwoman (Male to female transgender)
4 = Transgender male/transman (Female to male transgender)
5 = Androgynous/ambigender
6 = Questioning
7 = Genderqueer/genderfluid
8 = Other
97 = Don't Know
98 = Refuse to Answer
99 = Not Applicable

Q454. Did [Response to Q452] have a penis?

P4PENIS P4 has penis
1

0 = No
1 = Yes

7 = Don't Know
8 = Refuse to Answer
9 = Not Applicable

Q455. How old was [Response to Q452]?

P4AGE — P4 Age
2

0 - 96 = range
97 = Don't Know
98 = Refuse to Answer
99 = Not Applicable

Q456. What type of partner was [Response to Q452]?

Main Partner: Someone who is your primary sexual partner and you feel committed to (boyfriend, lover, husband, girlfriend, wife).

Casual Partner: Someone you have sex with, but don't feel committed to or don't know very well.

Exchange partner/Trick: Someone you had sex with in exchange for things like money, goods, or a place to stay.

P4TYPE — P4 type
1

1 = Main partner
2 = Casual partner
3 = Exchange partner / Trick
4 = Sexual assault
7 = Don't Know
8 = Refuse to Answer
9 = Not Applicable

Q457. Did this partner pay you or did you pay this partner?

P4PAYOR — P4 paid/you paid partner
1

1 = I was paid
2 = I paid this partner
7 = Don't Know
8 = Refuse to Answer
9 = Not Applicable

Q458. Would you consider this partner to be trade?

P4TRADE — P4 considered trade
1

0 = No
1 = Yes
7 = Don't Know
8 = Refuse to Answer
9 = Not Applicable

Q459. What is [Response to Q452]'s race or ethnicity? Would you describe [Response to Q452] as:

[Do NOT read choices. Check ALL that apply]

P4RACE — P4 race
2

0 - 7 = range

97 = Don't Know
98 = Refuse to Answer
99 = Not Applicable

P4RACEA P4 race: Asian
1

0 = No
1 = Yes
7 = Don't Know
8 = Refuse to Answer
9 = Not Applicable

P4RACEB P4 race: Black / African American
1

0 = No
1 = Yes
7 = Don't Know
8 = Refuse to Answer
9 = Not Applicable

P4RACEC P4 race: Native American
1

0 = No
1 = Yes
7 = Don't Know
8 = Refuse to Answer
9 = Not Applicable

P4RACED P4 race: Native Hawaiian or Pacific Islander
1

0 = No
1 = Yes
7 = Don't Know
8 = Refuse to Answer
9 = Not Applicable

P4RACEE P4 race: White
1

0 = No
1 = Yes
7 = Don't Know
8 = Refuse to Answer
9 = Not Applicable

P4RACEF P4 race: Latino/Latina
1

0 = No
1 = Yes
7 = Don't Know
8 = Refuse to Answer
9 = Not Applicable

P4RACEG P4 race: Other
1

0 = No

1 = Yes
7 = Don't Know
8 = Refuse to Answer
9 = Not Applicable

Q460. Please specify race / ethnicity:

P4RACESP P4 other race
20

0 - 20 = range
97 = Don't Know
98 = Refuse to Answer
99 = Not Applicable

Q461. To your knowledge, in the past 6 months has [Response to Q452] had sex with other people who are:

[READ choices. Check ALL that apply.]

P4SEXP P4's sex partners
1

0 - 6 = range
7 = Don't Know
8 = Refuse to Answer
9 = Not Applicable

P4SEXPA P4's sex partners: Cisgender men
1

0 = No
1 = Yes
7 = Don't Know
8 = Refuse to Answer
9 = Not Applicable

P4SEXPB P4's sex partners: Cisgender women
1

0 = No
1 = Yes
7 = Don't Know
8 = Refuse to Answer
9 = Not Applicable

P4SEXPC P4's sex partners: Transgender men
1

0 = No
1 = Yes
7 = Don't Know
8 = Refuse to Answer
9 = Not Applicable

P4SEXPD P4's sex partners: Transgender women
1

0 = No
1 = Yes
7 = Don't Know
8 = Refuse to Answer

9 = Not Applicable

P4SEXPE P4's sex partners: Gender non-conforming or gender non-binary
1

0 = No
1 = Yes
7 = Don't Know
8 = Refuse to Answer
9 = Not Applicable

P4SEXPF P4's sex partners: None
1

0 = No
1 = Yes
7 = Don't Know
8 = Refuse to Answer
9 = Not Applicable

Q462. Where did you first meet [Response to Q452]? [Do NOT read choices. Check ONE.]

P4MET P4 where met
2

1 = Bar
2 = Cafe or restaurant
3 = Dance club
4 = Adult book/video store, retail, business
5 = Sex club
6 = Street, park, library, public, transportation
7 = Church, political function, social club
8 = Work or school
9 = Gym or athletic activity
10 = Online - Dating app (OKCupid, Tinder, etc.)
11 = Online - Craigslist
12 = Online - Other, please specify
13 = Dating services or newspaper
14 = Private party or social club
15 = Introduce by friends
16 = Some other way.
97 = Don't Know
98 = Refuse to Answer
99 = Not Applicable

Q463. Please specify on where online you have met [Response to Q452]:

P4METOSP P4 met online specify
50

0 - 50 = range
97 = Don't Know
98 = Refuse to Answer
99 = Not Applicable

Q464. Please specify on where you have met [Response to Q452]:

P4METSP P4 met specify
50

0 - 50 = range
97 = Don't Know
98 = Refuse to Answer
99 = Not Applicable

Q465. To the best of your knowledge what is [Response to Q452]'s HIV status?

P4SERO P4 sero status
1

1 = Negative
2 = Positive
3 = Unknown
7 = Don't Know
8 = Refuse to Answer
9 = Not Applicable

Q466. To your knowledge, was this partner taking PrEP? (anti-HIV medications)

P4PREP P4 taking prep
1

0 = No
1 = Yes
7 = Don't Know
8 = Refuse to Answer
9 = Not Applicable

Q467. To your knowledge, was this partner on antiretrovirals?

P4ART P4 on ART
1

0 = No
1 = Yes
7 = Don't Know
8 = Refuse to Answer
9 = Not Applicable

Q468. To your knowledge, was this partner virally suppressed or their viral load undetectable?

P4VIRSUP P4 virally suppressed
1

0 = No
1 = Yes
7 = Don't Know
8 = Refuse to Answer
9 = Not Applicable

Q469. Did you disclose your HIV status to this partner before having sex the first time?

P4DISCL Disclosed HIV status to P4
1

0 = No
1 = Yes
7 = Don't Know
8 = Refuse to Answer
9 = Not Applicable

Q470. Did you have vaginal sex with [Response to Q452] during the last 6 months?

P4VAG P4 vaginal sex
1

0 = No
1 = Yes
7 = Don't Know
8 = Refuse to Answer
9 = Not Applicable

Q471. How many times have you had insertive vaginal intercourse with [Response to Q452] in the past six months?

P4VAGI P4 # vaginal insertive sex
3

0 - 996 = range
997 = Don't Know
998 = Refuse to Answer
999 = Not Applicable

Q472. Of the [Response to Q471] times you had insertive vaginal sex with this partner,

how many times did you NOT use a condom?

P4VAGIC P4 # vag ins no condom
3

0 - 996 = range
997 = Don't Know
998 = Refuse to Answer
999 = Not Applicable

Q473. Of the [Response to Q472] times you had insertive vaginal sex without a condom,

how many times were you high or drunk?

P4VAGIH P4 # vag ins unprot sex high or drunk
3

0 - 996 = range
997 = Don't Know
998 = Refuse to Answer
999 = Not Applicable

Q474. Now think of the last time you had insertive vaginal sex with [Response to Q452], did you use a condom?

P4VAGICL P4 last ins vag no condom
1

0 = No
1 = Yes
7 = Don't Know
8 = Refuse to Answer
9 = Not Applicable

Q475. Were you drinking or using drugs in the 2 hours before or during the last time you had insertive vaginal sex?

P4VAGIHL P4 last vag ins sex high or drunk
1

0 = No
1 = Yes
7 = Don't Know

8 = Refuse to Answer
9 = Not Applicable

Q476. What substances were you using?

P4VISUB P4 last vag ins what substances
2

0 - 18 = range
97 = Don't Know
98 = Refuse to Answer
99 = Not Applicable

P4VISUBA P4 last vag ins what substances: Alcohol
1

0 = No
1 = Yes
7 = Don't Know
8 = Refuse to Answer
9 = Not Applicable

P4VISUBB P4 last vag ins what substances: Meth
1

0 = No
1 = Yes
7 = Don't Know
8 = Refuse to Answer
9 = Not Applicable

P4VISUBC P4 last vag ins what substances: Crack
1

0 = No
1 = Yes
7 = Don't Know
8 = Refuse to Answer
9 = Not Applicable

P4VISUBD P4 last vag ins what substances: Cocaine
1

0 = No
1 = Yes
7 = Don't Know
8 = Refuse to Answer
9 = Not Applicable

P4VISUBE P4 last vag ins what substances: Poppers
1

0 = No
1 = Yes
7 = Don't Know
8 = Refuse to Answer
9 = Not Applicable

P4VISUBF P4 last vag ins what substances: GHB
1

0 = No

1 = Yes
7 = Don't Know
8 = Refuse to Answer
9 = Not Applicable

P4VISUBG P4 last vag ins what substances: Heroin
1

0 = No
1 = Yes
7 = Don't Know
8 = Refuse to Answer
9 = Not Applicable

P4VISUBH P4 last vag ins what substances: Marijuana
1

0 = No
1 = Yes
7 = Don't Know
8 = Refuse to Answer
9 = Not Applicable

P4VISUBI P4 last vag ins what substances: Synthetic cannabinoids
1

0 = No
1 = Yes
7 = Don't Know
8 = Refuse to Answer
9 = Not Applicable

P4VISUBJ P4 last vag ins what substances: Hallucinogens
1

0 = No
1 = Yes
7 = Don't Know
8 = Refuse to Answer
9 = Not Applicable

P4VISUBK P4 last vag ins what substances: Ecstasy
1

0 = No
1 = Yes
7 = Don't Know
8 = Refuse to Answer
9 = Not Applicable

P4VISUBL P4 last vag ins what substances: Rohypnol (roofies)
1

0 = No
1 = Yes
7 = Don't Know
8 = Refuse to Answer
9 = Not Applicable

P4VISUBM P4 last vag ins what substances: Ketamine

1

0 = No
1 = Yes
7 = Don't Know
8 = Refuse to Answer
9 = Not Applicable

P4VISUBN P4 last vag ins what substances: Benzos or barbituates
1

0 = No
1 = Yes
7 = Don't Know
8 = Refuse to Answer
9 = Not Applicable

P4VISUBO P4 last vag ins what substances: Painkillers
1

0 = No
1 = Yes
7 = Don't Know
8 = Refuse to Answer
9 = Not Applicable

P4VISUBP P4 last vag ins what substances: Viagra
1

0 = No
1 = Yes
7 = Don't Know
8 = Refuse to Answer
9 = Not Applicable

P4VISUBQ P4 last vag ins what substances: Prescription drugs
1

0 = No
1 = Yes
7 = Don't Know
8 = Refuse to Answer
9 = Not Applicable

P4VISUBR P4 last vag ins what substances: Other
1

0 = No
1 = Yes
7 = Don't Know
8 = Refuse to Answer
9 = Not Applicable

Q477. How many times have you had receptive vaginal intercourse with [Response to Q452] in the past six months?

P4VAGR P4 # vaginal receptive sex
3

0 - 996 = range
997 = Don't Know

998 = Refuse to Answer
999 = Not Applicable

Q478. Of the [Response to Q477] times you had receptive vaginal sex, how many times did you NOT use a condom?

P4VAGRC P4 # vag rec no condom 3

0 - 996 = range
997 = Don't Know
998 = Refuse to Answer
999 = Not Applicable

Q479. Of the [Response to Q478] times you had receptive vaginal sex without a condom, how many times were you high or drunk?

P4VAGRH P4 # vag rec unprot sex high or drunk 3

0 - 996 = range
997 = Don't Know
998 = Refuse to Answer
999 = Not Applicable

Q480. Now think of the last time you had receptive vaginal sex with [Response to Q452], did you use a condom?

P4VAGRCL P4 last rec vag no condom 1

0 = No
1 = Yes
7 = Don't Know
8 = Refuse to Answer
9 = Not Applicable

Q481. Were you drinking or using drugs in the 2 hours before or during the last time you had receptive vaginal sex?

P4VAGRHL P4 last vag rec sex high or drunk 1

0 = No
1 = Yes
7 = Don't Know
8 = Refuse to Answer
9 = Not Applicable

Q482. What substances were you using?

P4VRSUB P4 last vag rec what substances 2

0 - 18 = range
97 = Don't Know
98 = Refuse to Answer
99 = Not Applicable

P4VRSUBA P4 last vag rec what substances: Alcohol 1

0 = No
1 = Yes

7 = Don't Know
8 = Refuse to Answer
9 = Not Applicable

P4VRSUBB P4 last vag rec what substances: Meth
1

0 = No
1 = Yes
7 = Don't Know
8 = Refuse to Answer
9 = Not Applicable

P4VRSUBC P4 last vag rec what substances: Crack
1

0 = No
1 = Yes
7 = Don't Know
8 = Refuse to Answer
9 = Not Applicable

P4VRSUBD P4 last vag rec what substances: Cocaine
1

0 = No
1 = Yes
7 = Don't Know
8 = Refuse to Answer
9 = Not Applicable

P4VRSUBE P4 last vag rec what substances: Poppers
1

0 = No
1 = Yes
7 = Don't Know
8 = Refuse to Answer
9 = Not Applicable

P4VRSUBF P4 last vag rec what substances: GHB
1

0 = No
1 = Yes
7 = Don't Know
8 = Refuse to Answer
9 = Not Applicable

P4VRSUBG P4 last vag rec what substances: Heroin
1

0 = No
1 = Yes
7 = Don't Know
8 = Refuse to Answer
9 = Not Applicable

P4VRSUBH P4 last vag rec what substances: Marijuana
1

0 = No
1 = Yes
7 = Don't Know
8 = Refuse to Answer
9 = Not Applicable

P4VRSUBI P4 last vag rec what substances: Synthetic cannabinoids 1

0 = No
1 = Yes
7 = Don't Know
8 = Refuse to Answer
9 = Not Applicable

P4VRSUBJ P4 last vag rec what substances: Hallucinogens 1

0 = No
1 = Yes
7 = Don't Know
8 = Refuse to Answer
9 = Not Applicable

P4VRSUBK P4 last vag rec what substances: Ecstasy 1

0 = No
1 = Yes
7 = Don't Know
8 = Refuse to Answer
9 = Not Applicable

P4VRSUBL P4 last vag rec what substances: Rohypnol (roofies) 1

0 = No
1 = Yes
7 = Don't Know
8 = Refuse to Answer
9 = Not Applicable

P4VRSUBM P4 last vag rec what substances: Ketamine 1

0 = No
1 = Yes
7 = Don't Know
8 = Refuse to Answer
9 = Not Applicable

P4VRSUBN P4 last vag rec what substances: Benzos or barbituates 1

0 = No
1 = Yes
7 = Don't Know
8 = Refuse to Answer
9 = Not Applicable

P4VRSUBO P4 last vag rec what substances: Painkillers 1

0 = No
1 = Yes
7 = Don't Know
8 = Refuse to Answer
9 = Not Applicable

P4VRSUBP P4 last vag rec what substances: Viagra 1

0 = No
1 = Yes
7 = Don't Know
8 = Refuse to Answer
9 = Not Applicable

P4VRSUBQ P4 last vag rec what substances: Prescription drugs 1

0 = No
1 = Yes
7 = Don't Know
8 = Refuse to Answer
9 = Not Applicable

P4VRSUBR P4 last vag rec what substances: Other 1

0 = No
1 = Yes
7 = Don't Know
8 = Refuse to Answer
9 = Not Applicable

Q483. Did you have anal sex with [Response to Q452] during the last 6 months?

P4ANAL P4 anal sex 1

0 = No
1 = Yes
7 = Don't Know
8 = Refuse to Answer
9 = Not Applicable

Q484. How many times have you had insertive anal intercourse with [Response to Q452] in the past 6 months?

P4ANAI P4 # insertive anal sex 3

0 - 996 = range
997 = Don't Know
998 = Refuse to Answer
999 = Not Applicable

Q485. Of the [Response to Q484] times you had insertive anal sex,

how many times did you NOT use a condom?

P4ANAIC P4 # anal ins no condom 3

0 - 996 = range
997 = Don't Know
998 = Refuse to Answer
999 = Not Applicable

Q486. Of the [Response to Q485] times you had insertive anal sex without a condom, how many times were you high or drunk?

P4ANAIH P4 # anal ins unprot sex high or drunk 3

0 - 996 = range
997 = Don't Know
998 = Refuse to Answer
999 = Not Applicable

Q487. Now think of the last time you had insertive anal sex with [Response to Q452], did you use a condom?

P4ANAICL P4 last ins anal no condom 1

0 = No
1 = Yes
7 = Don't Know
8 = Refuse to Answer
9 = Not Applicable

Q488. Were you drinking or using drugs in the 2 hours before or during the last time you had insertive anal sex?

P4ANAIHL P4 last anal ins sex high or drunk 1

0 = No
1 = Yes
7 = Don't Know
8 = Refuse to Answer
9 = Not Applicable

Q489. What substances were you using?

P4AISUB P4 last anal ins what substances 2

0 - 18 = range
97 = Don't Know
98 = Refuse to Answer
99 = Not Applicable

P4AISUBA P4 last anal ins what substances: Alcohol 1

0 = No
1 = Yes
7 = Don't Know
8 = Refuse to Answer
9 = Not Applicable

P4AISUBB P4 last anal ins what substances: Meth

1

0 = No
1 = Yes
7 = Don't Know
8 = Refuse to Answer
9 = Not Applicable

P4AISUBC P4 last anal ins what substances: Crack
1

0 = No
1 = Yes
7 = Don't Know
8 = Refuse to Answer
9 = Not Applicable

P4AISUBD P4 last anal ins what substances: Cocaine
1

0 = No
1 = Yes
7 = Don't Know
8 = Refuse to Answer
9 = Not Applicable

P4AISUBE P4 last anal ins what substances: Poppers
1

0 = No
1 = Yes
7 = Don't Know
8 = Refuse to Answer
9 = Not Applicable

P4AISUBF P4 last anal ins what substances: GHB
1

0 = No
1 = Yes
7 = Don't Know
8 = Refuse to Answer
9 = Not Applicable

P4AISUBG P4 last anal ins what substances: Heroin
1

0 = No
1 = Yes
7 = Don't Know
8 = Refuse to Answer
9 = Not Applicable

P4AISUBH P4 last anal ins what substances: Marijuana
1

0 = No
1 = Yes
7 = Don't Know
8 = Refuse to Answer
9 = Not Applicable

P4AISUBI P4 last anal ins what substances: Synthetic cannabinoids 1

0 = No
1 = Yes
7 = Don't Know
8 = Refuse to Answer
9 = Not Applicable

P4AISUBJ P4 last anal ins what substances: Hallucinogens 1

0 = No
1 = Yes
7 = Don't Know
8 = Refuse to Answer
9 = Not Applicable

P4AISUBK P4 last anal ins what substances: Ecstasy 1

0 = No
1 = Yes
7 = Don't Know
8 = Refuse to Answer
9 = Not Applicable

P4AISUBL P4 last anal ins what substances: Rohypnol (roofies) 1

0 = No
1 = Yes
7 = Don't Know
8 = Refuse to Answer
9 = Not Applicable

P4AISUBM P4 last anal ins what substances: Ketamine 1

0 = No
1 = Yes
7 = Don't Know
8 = Refuse to Answer
9 = Not Applicable

P4AISUBN P4 last anal ins what substances: Benzos or barbituates 1

0 = No
1 = Yes
7 = Don't Know
8 = Refuse to Answer
9 = Not Applicable

P4AISUBO P4 last anal ins what substances: Painkillers 1

0 = No
1 = Yes
7 = Don't Know

8 = Refuse to Answer
9 = Not Applicable

P4AISUBP P4 last anal ins what substances: Viagra
1

0 = No
1 = Yes
7 = Don't Know
8 = Refuse to Answer
9 = Not Applicable

P4AISUBQ P4 last anal ins what substances: Prescription drugs
1

0 = No
1 = Yes
7 = Don't Know
8 = Refuse to Answer
9 = Not Applicable

P4AISUBR P4 last anal ins what substances: Other
1

0 = No
1 = Yes
7 = Don't Know
8 = Refuse to Answer
9 = Not Applicable

Q490. How many times have you had receptive anal intercourse with [Response to Q452] in the past six months.

P4ANAR P4 # receptive anal sex
3

0 - 996 = range
997 = Don't Know
998 = Refuse to Answer
999 = Not Applicable

Q491. Of the [Response to Q490] times you had receptive anal sex,

how many times did you NOT use a condom?

P4ANARC P4 # anal rec no condom
3

0 - 996 = range
997 = Don't Know
998 = Refuse to Answer
999 = Not Applicable

Q492. Of the [Response to Q491] times you had receptive anal sex without a condom,

how many times were you high or drunk?

P4ANARH P4 # anal rec unprot sex high or drunk
3

0 - 996 = range
997 = Don't Know

998 = Refuse to Answer
999 = Not Applicable

Q493. Now think of the last time you had receptive anal sex with [Response to Q452], did you use a condom?

P4ANARCL P4 last rec anal no condom
1

0 = No
1 = Yes
7 = Don't Know
8 = Refuse to Answer
9 = Not Applicable

Q494. Were you drinking or using drugs in the 2 hours before or during the last time you had receptive anal sex?

P4ANARHL P4 last anal rec sex high or drunk
1

0 = No
1 = Yes
7 = Don't Know
8 = Refuse to Answer
9 = Not Applicable

Q495. What substances were you using?

P4ARSUB P4 last anal rec what substances
2

0 - 18 = range
97 = Don't Know
98 = Refuse to Answer
99 = Not Applicable

P4ARSUBA P4 last anal rec what substances: Alcohol
1

0 = No
1 = Yes
7 = Don't Know
8 = Refuse to Answer
9 = Not Applicable

P4ARSUBB P4 last anal rec what substances: Meth
1

0 = No
1 = Yes
7 = Don't Know
8 = Refuse to Answer
9 = Not Applicable

P4ARSUBC P4 last anal rec what substances: Crack
1

0 = No
1 = Yes
7 = Don't Know
8 = Refuse to Answer
9 = Not Applicable

P4ARSUBD P4 last anal rec what substances: Cocaine 1

0 = No
1 = Yes
7 = Don't Know
8 = Refuse to Answer
9 = Not Applicable

P4ARSUBE P4 last anal rec what substances: Poppers 1

0 = No
1 = Yes
7 = Don't Know
8 = Refuse to Answer
9 = Not Applicable

P4ARSUBF P4 last anal rec what substances: GHB 1

0 = No
1 = Yes
7 = Don't Know
8 = Refuse to Answer
9 = Not Applicable

P4ARSUBG P4 last anal rec what substances: Heroin 1

0 = No
1 = Yes
7 = Don't Know
8 = Refuse to Answer
9 = Not Applicable

P4ARSUBH P4 last anal rec what substances: Marijuana 1

0 = No
1 = Yes
7 = Don't Know
8 = Refuse to Answer
9 = Not Applicable

P4ARSUBI P4 last anal rec what substances: Synthetic cannabinoids 1

0 = No
1 = Yes
7 = Don't Know
8 = Refuse to Answer
9 = Not Applicable

P4ARSUBJ P4 last anal rec what substances: Hallucinogens 1

0 = No
1 = Yes
7 = Don't Know

8 = Refuse to Answer
9 = Not Applicable

P4ARSUBK P4 last anal rec what substances: Ecstasy
1

0 = No
1 = Yes
7 = Don't Know
8 = Refuse to Answer
9 = Not Applicable

P4ARSUBL P4 last anal rec what substances: Rohypnol (roofies)
1

0 = No
1 = Yes
7 = Don't Know
8 = Refuse to Answer
9 = Not Applicable

P4ARSUBM P4 last anal rec what substances: Ketamine
1

0 = No
1 = Yes
7 = Don't Know
8 = Refuse to Answer
9 = Not Applicable

P4ARSUBN P4 last anal rec what substances: Benzos or barbituates
1

0 = No
1 = Yes
7 = Don't Know
8 = Refuse to Answer
9 = Not Applicable

P4ARSUBO P4 last anal rec what substances: Painkillers
1

0 = No
1 = Yes
7 = Don't Know
8 = Refuse to Answer
9 = Not Applicable

P4ARSUBP P4 last anal rec what substances: Viagra
1

0 = No
1 = Yes
7 = Don't Know
8 = Refuse to Answer
9 = Not Applicable

P4ARSUBQ P4 last anal rec what substances: Prescription drugs
1

0 = No

1 = Yes
7 = Don't Know
8 = Refuse to Answer
9 = Not Applicable

P4ARSUBR P4 last anal rec what substances: Other
1

0 = No
1 = Yes
7 = Don't Know
8 = Refuse to Answer
9 = Not Applicable

Q496. Does this partner inject drugs?

P4INJ P4 inject drugs
1

0 = No
1 = Yes
7 = Don't Know
8 = Refuse to Answer
9 = Not Applicable

Q497. To help guide you through the questions, please provide initials for this next person: we will refer to this person later using the initials you provide.

P5IN P5 Initials
20

0 - 20 = range
97 = Don't Know
98 = Refuse to Answer
99 = Not Applicable

Q498. What is [Response to Q497]s gender? [Do NOT read choices. Check ONE.]

P5GEN P5 gender
2

1 = Cisgender male
2 = Cisgender female
3 = Transgender female / transwoman (Male to female transgender)
4 = Transgender male/transman (Female to male transgender)
5 = Androgynous/ambigender
6 = Questioning
7 = Genderqueer/genderfluid
8 = Other
97 = Don't Know
98 = Refuse to Answer
99 = Not Applicable

Q499. Did [Response to Q497] have a penis?

P5PENIS P5 has penis
1

0 = No

1 = Yes
7 = Don't Know
8 = Refuse to Answer
9 = Not Applicable

Q500. How old was [Response to Q497]?

P5AGE P5 Age 2

0 - 96 = range
97 = Don't Know
98 = Refuse to Answer
99 = Not Applicable

Q501. What type of partner was [Response to Q497]?

Main Partner: Someone who is your primary sexual partner and you feel committed to (boyfriend, lover, husband, girlfriend, wife).

Casual Partner: Someone you have sex with, but don't feel committed to or don't know very well.

Exchange partner/Trick: Someone you had sex with in exchange for things like money, goods, or a place to stay.

P5TYPE P5 type 1

1 = Main partner
2 = Casual partner
3 = Exchange partner / Trick
4 = Sexual assault
7 = Don't Know
8 = Refuse to Answer
9 = Not Applicable

Q502. Did this partner pay you or did you pay this partner?

P5PAYOR P5 paid/you paid partner 1

1 = I was paid
2 = I paid this partner
7 = Don't Know
8 = Refuse to Answer
9 = Not Applicable

Q503. Would you consider this partner to be trade?

P5TRADE P5 considered trade 1

0 = No
1 = Yes
7 = Don't Know
8 = Refuse to Answer
9 = Not Applicable

Q504. What is [Response to Q497]'s race or ethnicity? Would you describe [Response to Q497] as:

[Do NOT read choices. Check ALL that apply]

P5RACE P5 race 2

0 - 7 = range
97 = Don't Know
98 = Refuse to Answer
99 = Not Applicable

P5RACEA P5 race: Asian
1

0 = No
1 = Yes
7 = Don't Know
8 = Refuse to Answer
9 = Not Applicable

P5RACEB P5 race: Black / African American
1

0 = No
1 = Yes
7 = Don't Know
8 = Refuse to Answer
9 = Not Applicable

P5RACEC P5 race: Native American
1

0 = No
1 = Yes
7 = Don't Know
8 = Refuse to Answer
9 = Not Applicable

P5RACED P5 race: Native Hawaiian or Pacific Islander
1

0 = No
1 = Yes
7 = Don't Know
8 = Refuse to Answer
9 = Not Applicable

P5RACEE P5 race: White
1

0 = No
1 = Yes
7 = Don't Know
8 = Refuse to Answer
9 = Not Applicable

P5RACEF P5 race: Latino/Latina
1

0 = No
1 = Yes
7 = Don't Know
8 = Refuse to Answer
9 = Not Applicable

P5RACEG P5 race: Other
1

0 = No
1 = Yes
7 = Don't Know
8 = Refuse to Answer
9 = Not Applicable

Q505. Please specify race / ethnicity:

P5RACESP P5 other race
20

0 - 20 = range
97 = Don't Know
98 = Refuse to Answer
99 = Not Applicable

Q506. To your knowledge, in the past 6 months has [Response to Q497] had sex with other people who are:

[READ choices. Check ALL that apply.]

P5SEXP P5's sex partners
1

0 - 6 = range
7 = Don't Know
8 = Refuse to Answer
9 = Not Applicable

P5SEXPA P5's sex partners: Cisgender men
1

0 = No
1 = Yes
7 = Don't Know
8 = Refuse to Answer
9 = Not Applicable

P5SEXPB P5's sex partners: Cisgender women
1

0 = No
1 = Yes
7 = Don't Know
8 = Refuse to Answer
9 = Not Applicable

P5SEXPC P5's sex partners: Transgender men
1

0 = No
1 = Yes
7 = Don't Know
8 = Refuse to Answer
9 = Not Applicable

P5SEXPD P5's sex partners: Transgender women
1

0 = No
1 = Yes
7 = Don't Know

8 = Refuse to Answer
9 = Not Applicable

P5SEXPE P5's sex partners: Gender non-conforming or gender non-binary
1

0 = No
1 = Yes
7 = Don't Know
8 = Refuse to Answer
9 = Not Applicable

P5SEXPF P5's sex partners: None
1

0 = No
1 = Yes
7 = Don't Know
8 = Refuse to Answer
9 = Not Applicable

Q507. Where did you first meet [Response to Q497]? [Do NOT read choices. Check ONE.]

P5MET P5 where met
2

1 = Bar
2 = Cafe or restaurant
3 = Dance club
4 = Adult book/video store, retail, business
5 = Sex club
6 = Street, park, library, public, transportation
7 = Church, political function, social club
8 = Work or school
9 = Gym or athletic activity
10 = Online - Dating app (OKCupid, Tinder, etc.)
11 = Online - Craigslist
12 = Online - Other, please specify
13 = Dating services or newspaper
14 = Private party or social club
15 = Introduce by friends
16 = Some other way.
97 = Don't Know
98 = Refuse to Answer
99 = Not Applicable

Q508. Please specify on where online you have met [Response to Q497]:

P5METOSP P5 met online specify
50

0 - 50 = range
97 = Don't Know
98 = Refuse to Answer
99 = Not Applicable

Q509. Please specify on where you have met [Response to Q497]:

P5METSP P5 met specify

0 - 50 = range
97 = Don't Know
98 = Refuse to Answer
99 = Not Applicable

Q510. To the best of your knowledge what is [Response to Q497]'s HIV status?

P5SERO P5 sero status
1

1 = Negative
2 = Positive
3 = Unknown
7 = Don't Know
8 = Refuse to Answer
9 = Not Applicable

Q511. To your knowledge, was this partner taking PrEP? (anti-HIV medications)

P5PREP P5 taking prep
1

0 = No
1 = Yes
7 = Don't Know
8 = Refuse to Answer
9 = Not Applicable

Q512. To your knowledge, was this partner on antiretrovirals?

P5ART P5 on ART
1

0 = No
1 = Yes
7 = Don't Know
8 = Refuse to Answer
9 = Not Applicable

Q513. To your knowledge, was this partner virally suppressed or their viral load undetectable?

P5VIRSUP P5 virally suppressed
1

0 = No
1 = Yes
7 = Don't Know
8 = Refuse to Answer
9 = Not Applicable

Q514. Did you disclose your HIV status to this partner before having sex the first time?

P5DISCL Disclosed HIV status to P5
1

0 = No
1 = Yes
7 = Don't Know
8 = Refuse to Answer
9 = Not Applicable

Q515. Did you have vaginal sex with [Response to Q497] during the last 6 months?

P5VAG P5 vaginal sex
1

0 = No
1 = Yes
7 = Don't Know
8 = Refuse to Answer
9 = Not Applicable

Q516. How many times have you had insertive vaginal intercourse with [Response to Q497] in the past six months?

P5VAGI P5 # vaginal insertive sex
3

0 - 996 = range
997 = Don't Know
998 = Refuse to Answer
999 = Not Applicable

Q517. Of the [Response to Q516] times you had insertive vaginal sex with this partner,

how many times did you NOT use a condom?

P5VAGIC P5 # vag ins no condom
3

0 - 996 = range
997 = Don't Know
998 = Refuse to Answer
999 = Not Applicable

Q518. Of the [Response to Q517] times you had insertive vaginal sex without a condom,

how many times were you high or drunk?

P5VAGIH P5 # vag ins unprot sex high or drunk
3

0 - 996 = range
997 = Don't Know
998 = Refuse to Answer
999 = Not Applicable

Q519. Now think of the last time you had insertive vaginal sex with [Response to Q497], did you use a condom?

P5VAGICL P5 last ins vag no condom
1

0 = No
1 = Yes
7 = Don't Know
8 = Refuse to Answer
9 = Not Applicable

Q520. Were you drinking or using drugs in the 2 hours before or during the last time you had insertive vaginal sex?

P5VAGIHL P5 last vag ins sex high or drunk
1

0 = No

1 = Yes
7 = Don't Know
8 = Refuse to Answer
9 = Not Applicable

Q521. What substances were you using?

P5VISUB P5 last vag ins what substances
2

0 - 18 = range
97 = Don't Know
98 = Refuse to Answer
99 = Not Applicable

P5VISUBA P5 last vag ins what substances: Alcohol
1

0 = No
1 = Yes
7 = Don't Know
8 = Refuse to Answer
9 = Not Applicable

P5VISUBB P5 last vag ins what substances: Meth
1

0 = No
1 = Yes
7 = Don't Know
8 = Refuse to Answer
9 = Not Applicable

P5VISUBC P5 last vag ins what substances: Crack
1

0 = No
1 = Yes
7 = Don't Know
8 = Refuse to Answer
9 = Not Applicable

P5VISUBD P5 last vag ins what substances: Cocaine
1

0 = No
1 = Yes
7 = Don't Know
8 = Refuse to Answer
9 = Not Applicable

P5VISUBE P5 last vag ins what substances: Poppers
1

0 = No
1 = Yes
7 = Don't Know
8 = Refuse to Answer
9 = Not Applicable

P5VISUBF P5 last vag ins what substances: GHB

1

0 = No
1 = Yes
7 = Don't Know
8 = Refuse to Answer
9 = Not Applicable

P5VISUBG P5 last vag ins what substances: Heroin
1

0 = No
1 = Yes
7 = Don't Know
8 = Refuse to Answer
9 = Not Applicable

P5VISUBH P5 last vag ins what substances: Marijuana
1

0 = No
1 = Yes
7 = Don't Know
8 = Refuse to Answer
9 = Not Applicable

P5VISUBI P5 last vag ins what substances: Synthetic cannabinoids
1

0 = No
1 = Yes
7 = Don't Know
8 = Refuse to Answer
9 = Not Applicable

P5VISUBJ P5 last vag ins what substances: Hallucinogens
1

0 = No
1 = Yes
7 = Don't Know
8 = Refuse to Answer
9 = Not Applicable

P5VISUBK P5 last vag ins what substances: Ecstasy
1

0 = No
1 = Yes
7 = Don't Know
8 = Refuse to Answer
9 = Not Applicable

P5VISUBL P5 last vag ins what substances: Rohypnol (roofies)
1

0 = No
1 = Yes
7 = Don't Know
8 = Refuse to Answer
9 = Not Applicable

P5VISUBM P5 last vag ins what substances: Ketamine
1

0 = No
1 = Yes
7 = Don't Know
8 = Refuse to Answer
9 = Not Applicable

P5VISUBN P5 last vag ins what substances: Benzos or barbituates
1

0 = No
1 = Yes
7 = Don't Know
8 = Refuse to Answer
9 = Not Applicable

P5VISUBO P5 last vag ins what substances: Painkillers
1

0 = No
1 = Yes
7 = Don't Know
8 = Refuse to Answer
9 = Not Applicable

P5VISUBP P5 last vag ins what substances: Viagra
1

0 = No
1 = Yes
7 = Don't Know
8 = Refuse to Answer
9 = Not Applicable

P5VISUBQ P5 last vag ins what substances: Prescription drugs
1

0 = No
1 = Yes
7 = Don't Know
8 = Refuse to Answer
9 = Not Applicable

P5VISUBR P5 last vag ins what substances: Other
1

0 = No
1 = Yes
7 = Don't Know
8 = Refuse to Answer
9 = Not Applicable

Q522. How many times have you had receptive vaginal intercourse with [Response to Q497] in the past six months?

P5VAGR P5 # vaginal receptive sex
3

0 - 996 = range
997 = Don't Know
998 = Refuse to Answer
999 = Not Applicable

Q523. Of the [Response to Q522] times you had receptive vaginal sex, how many times did you NOT use a condom?

P5VAGRC P5 # vag rec no condom
3

0 - 996 = range
997 = Don't Know
998 = Refuse to Answer
999 = Not Applicable

Q524. Of the [Response to Q523] times you had receptive vaginal sex without a condom, how many times were you high or drunk?

P5VAGRH P5 # vag rec unprot sex high or drunk
3

0 - 996 = range
997 = Don't Know
998 = Refuse to Answer
999 = Not Applicable

Q525. Now think of the last time you had receptive vaginal sex with [Response to Q497], did you use a condom?

P5VAGRCL P5 last rec vag no condom
1

0 = No
1 = Yes
7 = Don't Know
8 = Refuse to Answer
9 = Not Applicable

Q526. Were you drinking or using drugs in the 2 hours before or during the last time you had receptive vaginal sex?

P5VAGRHL P5 last vag rec sex high or drunk
1

0 = No
1 = Yes
7 = Don't Know
8 = Refuse to Answer
9 = Not Applicable

Q527. What substances were you using?

P5VRSUB P5 last vag rec what substances
2

0 - 18 = range
97 = Don't Know
98 = Refuse to Answer
99 = Not Applicable

P5VRSUBA P5 last vag rec what substances: Alcohol
1

0 = No
1 = Yes
7 = Don't Know
8 = Refuse to Answer
9 = Not Applicable

P5VRSUBB P5 last vag rec what substances: Meth
1

0 = No
1 = Yes
7 = Don't Know
8 = Refuse to Answer
9 = Not Applicable

P5VRSUBC P5 last vag rec what substances: Crack
1

0 = No
1 = Yes
7 = Don't Know
8 = Refuse to Answer
9 = Not Applicable

P5VRSUBD P5 last vag rec what substances: Cocaine
1

0 = No
1 = Yes
7 = Don't Know
8 = Refuse to Answer
9 = Not Applicable

P5VRSUBE P5 last vag rec what substances: Poppers
1

0 = No
1 = Yes
7 = Don't Know
8 = Refuse to Answer
9 = Not Applicable

P5VRSUBF P5 last vag rec what substances: GHB
1

0 = No
1 = Yes
7 = Don't Know
8 = Refuse to Answer
9 = Not Applicable

P5VRSUBG P5 last vag rec what substances: Heroin
1

0 = No
1 = Yes
7 = Don't Know
8 = Refuse to Answer
9 = Not Applicable

P5VRSUBH P5 last vag rec what substances: Marijuana
1

0 = No
1 = Yes
7 = Don't Know
8 = Refuse to Answer
9 = Not Applicable

P5VRSUBI P5 last vag rec what substances: Synthetic cannabinoids
1

0 = No
1 = Yes
7 = Don't Know
8 = Refuse to Answer
9 = Not Applicable

P5VRSUBJ P5 last vag rec what substances: Hallucinogens
1

0 = No
1 = Yes
7 = Don't Know
8 = Refuse to Answer
9 = Not Applicable

P5VRSUBK P5 last vag rec what substances: Ecstasy
1

0 = No
1 = Yes
7 = Don't Know
8 = Refuse to Answer
9 = Not Applicable

P5VRSUBL P5 last vag rec what substances: Rohypnol (roofies)
1

0 = No
1 = Yes
7 = Don't Know
8 = Refuse to Answer
9 = Not Applicable

P5VRSUBM P5 last vag rec what substances: Ketamine
1

0 = No
1 = Yes
7 = Don't Know
8 = Refuse to Answer
9 = Not Applicable

P5VRSUBN P5 last vag rec what substances: Benzos or barbituates
1

0 = No
1 = Yes
7 = Don't Know
8 = Refuse to Answer

9 = Not Applicable

P5VRSUBO P5 last vag rec what substances: Painkillers
1

0 = No
1 = Yes
7 = Don't Know
8 = Refuse to Answer
9 = Not Applicable

P5VRSUBP P5 last vag rec what substances: Viagra
1

0 = No
1 = Yes
7 = Don't Know
8 = Refuse to Answer
9 = Not Applicable

P5VRSUBQ P5 last vag rec what substances: Prescription drugs
1

0 = No
1 = Yes
7 = Don't Know
8 = Refuse to Answer
9 = Not Applicable

P5VRSUBR P5 last vag rec what substances: Other
1

0 = No
1 = Yes
7 = Don't Know
8 = Refuse to Answer
9 = Not Applicable

Q528. Did you have anal sex with [Response to Q497] during the last 6 months?

P5ANAL P5 anal sex
1

0 = No
1 = Yes
7 = Don't Know
8 = Refuse to Answer
9 = Not Applicable

Q529. How many times have you had insertive anal intercourse with [Response to Q497] in the past 6 months?

P5ANAI P5 # insertive anal sex
3

0 - 996 = range
997 = Don't Know
998 = Refuse to Answer
999 = Not Applicable

Q530. Of the [Response to Q529] times you had insertive anal sex, how many times did you NOT use a condom?

P5ANAIC P5 # anal ins no condom 3

0 - 996 = range
997 = Don't Know
998 = Refuse to Answer
999 = Not Applicable

Q531. Of the [Response to Q530] times you had insertive anal sex without a condom, how many times were you high or drunk?

P5ANAIH P5 # anal ins unprot sex high or drunk 3

0 - 996 = range
997 = Don't Know
998 = Refuse to Answer
999 = Not Applicable

Q532. Now think of the last time you had insertive anal sex with [Response to Q497], did you use a condom?

P5ANAICL P5 last ins anal no condom 1

0 = No
1 = Yes
7 = Don't Know
8 = Refuse to Answer
9 = Not Applicable

Q533. Were you drinking or using drugs in the 2 hours before or during the last time you had insertive anal sex?

P5ANAIHL P5 last anal ins sex high or drunk 1

0 = No
1 = Yes
7 = Don't Know
8 = Refuse to Answer
9 = Not Applicable

Q534. What substances were you using?

P5AISUB P5 last anal ins what substances 2

0 - 18 = range
97 = Don't Know
98 = Refuse to Answer
99 = Not Applicable

P5AISUBA P5 last anal ins what substances: Alcohol 1

0 = No
1 = Yes
7 = Don't Know
8 = Refuse to Answer
9 = Not Applicable

P5AISUBB P5 last anal ins what substances: Meth

1

0 = No
1 = Yes
7 = Don't Know
8 = Refuse to Answer
9 = Not Applicable

P5AISUBC P5 last anal ins what substances: Crack

1

0 = No
1 = Yes
7 = Don't Know
8 = Refuse to Answer
9 = Not Applicable

P5AISUBD P5 last anal ins what substances: Cocaine

1

0 = No
1 = Yes
7 = Don't Know
8 = Refuse to Answer
9 = Not Applicable

P5AISUBE P5 last anal ins what substances: Poppers

1

0 = No
1 = Yes
7 = Don't Know
8 = Refuse to Answer
9 = Not Applicable

P5AISUBF P5 last anal ins what substances: GHB

1

0 = No
1 = Yes
7 = Don't Know
8 = Refuse to Answer
9 = Not Applicable

P5AISUBG P5 last anal ins what substances: Heroin

1

0 = No
1 = Yes
7 = Don't Know
8 = Refuse to Answer
9 = Not Applicable

P5AISUBH P5 last anal ins what substances: Marijuana

1

0 = No
1 = Yes
7 = Don't Know

8 = Refuse to Answer
9 = Not Applicable

P5AISUBI P5 last anal ins what substances: Synthetic cannabinoids
1

0 = No
1 = Yes
7 = Don't Know
8 = Refuse to Answer
9 = Not Applicable

P5AISUBJ P5 last anal ins what substances: Hallucinogens
1

0 = No
1 = Yes
7 = Don't Know
8 = Refuse to Answer
9 = Not Applicable

P5AISUBK P5 last anal ins what substances: Ecstasy
1

0 = No
1 = Yes
7 = Don't Know
8 = Refuse to Answer
9 = Not Applicable

P5AISUBL P5 last anal ins what substances: Rohypnol (roofies)
1

0 = No
1 = Yes
7 = Don't Know
8 = Refuse to Answer
9 = Not Applicable

P5AISUBM P5 last anal ins what substances: Ketamine
1

0 = No
1 = Yes
7 = Don't Know
8 = Refuse to Answer
9 = Not Applicable

P5AISUBN P5 last anal ins what substances: Benzos or barbituates
1

0 = No
1 = Yes
7 = Don't Know
8 = Refuse to Answer
9 = Not Applicable

P5AISUBO P5 last anal ins what substances: Painkillers
1

0 = No

1 = Yes
7 = Don't Know
8 = Refuse to Answer
9 = Not Applicable

P5AISUBP P5 last anal ins what substances: Viagra
1

0 = No
1 = Yes
7 = Don't Know
8 = Refuse to Answer
9 = Not Applicable

P5AISUBQ P5 last anal ins what substances: Prescription drugs
1

0 = No
1 = Yes
7 = Don't Know
8 = Refuse to Answer
9 = Not Applicable

P5AISUBR P5 last anal ins what substances: Other
1

0 = No
1 = Yes
7 = Don't Know
8 = Refuse to Answer
9 = Not Applicable

Q535. How many times have you had receptive anal intercourse with [Response to Q497] in the past six months.

P5ANAR P5 # receptive anal sex
3

0 - 996 = range
997 = Don't Know
998 = Refuse to Answer
999 = Not Applicable

Q536. Of the [Response to Q535] times you had receptive anal sex,

how many times did you NOT use a condom?

P5ANARC P5 # anal rec no condom
3

0 - 996 = range
997 = Don't Know
998 = Refuse to Answer
999 = Not Applicable

Q537. Of the [Response to Q536] times you had receptive anal sex without a condom,

how many times were you high or drunk?

P5ANARH P5 # anal rec unprot sex high or drunk
3

0 - 996 = range
997 = Don't Know
998 = Refuse to Answer
999 = Not Applicable

Q538. Now think of the last time you had receptive anal sex with [Response to Q497], did you use a condom?

P5ANARCL P5 last rec anal no condom
1

0 = No
1 = Yes
7 = Don't Know
8 = Refuse to Answer
9 = Not Applicable

Q539. Were you drinking or using drugs in the 2 hours before or during the last time you had receptive anal sex?

P5ANARHL P5 last anal rec sex high or drunk
1

0 = No
1 = Yes
7 = Don't Know
8 = Refuse to Answer
9 = Not Applicable

Q540. What substances were you using?

P5ARSUB P5 last anal rec what substances
2

0 - 18 = range
97 = Don't Know
98 = Refuse to Answer
99 = Not Applicable

P5ARSUBA P5 last anal rec what substances: Alcohol
1

0 = No
1 = Yes
7 = Don't Know
8 = Refuse to Answer
9 = Not Applicable

P5ARSUBB P5 last anal rec what substances: Meth
1

0 = No
1 = Yes
7 = Don't Know
8 = Refuse to Answer
9 = Not Applicable

P5ARSUBC P5 last anal rec what substances: Crack
1

0 = No
1 = Yes
7 = Don't Know

8 = Refuse to Answer
9 = Not Applicable

P5ARSUBD P5 last anal rec what substances: Cocaine
1

0 = No
1 = Yes
7 = Don't Know
8 = Refuse to Answer
9 = Not Applicable

P5ARSUBE P5 last anal rec what substances: Poppers
1

0 = No
1 = Yes
7 = Don't Know
8 = Refuse to Answer
9 = Not Applicable

P5ARSUBF P5 last anal rec what substances: GHB
1

0 = No
1 = Yes
7 = Don't Know
8 = Refuse to Answer
9 = Not Applicable

P5ARSUBG P5 last anal rec what substances: Heroin
1

0 = No
1 = Yes
7 = Don't Know
8 = Refuse to Answer
9 = Not Applicable

P5ARSUBH P5 last anal rec what substances: Marijuana
1

0 = No
1 = Yes
7 = Don't Know
8 = Refuse to Answer
9 = Not Applicable

P5ARSUBI P5 last anal rec what substances: Synthetic cannabinoids
1

0 = No
1 = Yes
7 = Don't Know
8 = Refuse to Answer
9 = Not Applicable

P5ARSUBJ P5 last anal rec what substances: Hallucinogens
1

0 = No

1 = Yes
7 = Don't Know
8 = Refuse to Answer
9 = Not Applicable

P5ARSUBK P5 last anal rec what substances: Ecstasy
1

0 = No
1 = Yes
7 = Don't Know
8 = Refuse to Answer
9 = Not Applicable

P5ARSUBL P5 last anal rec what substances: Rohypnol (roofies)
1

0 = No
1 = Yes
7 = Don't Know
8 = Refuse to Answer
9 = Not Applicable

P5ARSUBM P5 last anal rec what substances: Ketamine
1

0 = No
1 = Yes
7 = Don't Know
8 = Refuse to Answer
9 = Not Applicable

P5ARSUBN P5 last anal rec what substances: Benzos or barbituates
1

0 = No
1 = Yes
7 = Don't Know
8 = Refuse to Answer
9 = Not Applicable

P5ARSUBO P5 last anal rec what substances: Painkillers
1

0 = No
1 = Yes
7 = Don't Know
8 = Refuse to Answer
9 = Not Applicable

P5ARSUBP P5 last anal rec what substances: Viagra
1

0 = No
1 = Yes
7 = Don't Know
8 = Refuse to Answer
9 = Not Applicable

P5ARSUBQ P5 last anal rec what substances: Prescription drugs

1

0 = No
1 = Yes
7 = Don't Know
8 = Refuse to Answer
9 = Not Applicable

P5ARSUBR P5 last anal rec what substances: Other
1

0 = No
1 = Yes
7 = Don't Know
8 = Refuse to Answer
9 = Not Applicable

Q541. Does this partner inject drugs?

P5INJ P5 inject drugs
1

0 = No
1 = Yes
7 = Don't Know
8 = Refuse to Answer
9 = Not Applicable

Q542. In your life, have you ever had a sexual partner who had previous or current transfemale sex partners?

FIREWORK Partner who had transfemale partners
1

0 = No
1 = Yes
7 = Don't Know
8 = Refuse to Answer
9 = Not Applicable

Q543. Have you ever been tested for HIV?

HIVTEST Tested for HIV
1

0 = No
1 = Yes
7 = Don't Know
8 = Refuse to Answer
9 = Not Applicable

Q544. When did you have your most recent HIV test?

MOSTRCT Date of most recent HIV test
6

1/1985 - Unlimited = mm/yyyy
2097 = Don't Know (Year)
2098 = Refuse to Answer (Year)
2099 = Not Applicable (Year)

Q545. When you got tested in [Response to Q544], where did you get tested? [Do NOT read

choices. Check ONE.]

WHRTEST Where most recently tested 2

- **1** = Tom Waddell (Trans Tuesdays)
- **2** = API Wellness (Trans Thrive)
- **3** = City Clinic
- **4** = Glide
- **5** = Kaiser
- **6** = SF General Hospital
- **7** = Ward 86/Positive Health Program
- **8** = Other
- **97** = Don't Know
- **98** = Refuse to Answer
- **99** = Not Applicable

Q546. Please specify where you were most recently tested for HIV.

WHTESTSP Where most recently tested specify 50

- **0 - 50** = range
- **97** = Don't Know
- **98** = Refuse to Answer
- **99** = Not Applicable

Q547. What was the result of your most recent HIV test? [Do NOT read choices. Check ONE.]

HIVRES Result of most recent HIV test 1

- **1** = Negative
- **2** = Positive
- **3** = Never obtained results
- **4** = Indeterminate
- **7** = Don't Know
- **8** = Refuse to Answer
- **9** = Not Applicable

Q548. Was this the first time you tested positive for HIV?

FIRSTPOS First time tested positive 1

- **0** = No
- **1** = Yes
- **7** = Don't Know
- **8** = Refuse to Answer
- **9** = Not Applicable

Q549. When did you first test positive for HIV?

WHNPOS When first tested positive 6

- **Unlimited - Unlimited** = mm/yyyy
- **2097** = Don't Know (Year)
- **2098** = Refuse to Answer (Year)
- **2099** = Not Applicable (Year)

Q550. What is the main reason you have not been tested for HIV in the past 6 months? [Write

down reason.]

NOTEST Why not tested past 6 months 250

0 - 250 = range
997 = Don't Know
998 = Refuse to Answer
999 = Not Applicable

Q551. INTERVIEWER: Please classify [Response to Q550] into one of the following categories. [Do NOT read choices. Check ONE.]

NOTEST2 Choose why no test in past 6 months 1

1 = Thought at low risk for HIV
2 = Didn't have money or insurance to pay for the test
3 = Cant find a place to get tested where feel welcome
4 = Felt unwelcome due to gender presentation
5 = Didnt think could get treatment if positive
6 = Other
7 = Don't Know
8 = Refuse to Answer
9 = Not Applicable

Q552. What do you believe your HIV status is today?

BELIVHIV Believe current HIV status 1

1 = Negative
2 = Positive
7 = Don't Know
8 = Refuse to Answer
9 = Not Applicable

Calculated Variable

HIVCHCK HIV status tested or believed

HIVCHCK = IF(HIVRES=2,"TESTED",IF(HIVRES=1,"NEGATIV",IF(BELIVHIV=2,"BELIEVE",IF(BELIVHIV=1,"NEGATIV","NEITHER"))))

Q553. Could you tell me in a couple sentences about where you were in your gender transition when you believe you contracted HIV? [Interviewer: probe for sexual orientation, gender identity/transition at time of acquisition.]

HIVGEND Gender transition at time contracted HIV 200

0 - 200 = range
997 = Don't Know
998 = Refuse to Answer
999 = Not Applicable

Q554. Who do you believe you contracted HIV from? [Interviewer: probe for partner characteristics (gender identity, perceived sexual orientation, partner type).]

HIVWHO Who contracted HIV from 200

0 - 200 = range

997 = Don't Know
998 = Refuse to Answer
999 = Not Applicable

Q555. Have you ever been seen by a doctor, nurse, or health care provider for a medical evaluation or care related to your HIV infection?

HIVCARE Ever received HIV care
1

0 = No
1 = Yes
7 = Don't Know
8 = Refuse to Answer
9 = Not Applicable

Q556. Are you currently seeing a health care provider for HIV care?

CURRCARE Currently receive HIV care
1

0 = No
1 = Yes
7 = Don't Know
8 = Refuse to Answer
9 = Not Applicable

Q557. Are you currently taking HIV medicines? These medicines are also known as antiretroviral medicines, ART, or HAART.

CURRMED Currently taking meds
1

0 = No
1 = Yes
7 = Don't Know
8 = Refuse to Answer
9 = Not Applicable

Q558. Have you ever had an HIV Viral Load Test?

VIRALLD Viral load test ever
1

0 = No
1 = Yes
7 = Don't Know
8 = Refuse to Answer
9 = Not Applicable

Q559. Was your most recent viral load detectable or undetectable?

VIRALLD2 Most recent viral load count
1

0 = Undetectable
1 = Detectable
7 = Don't Know
8 = Refuse to Answer
9 = Not Applicable

Q560. In the past 6 months, has a doctor, nurse or other health care provider told you that you had

any of the following STDs? [READ choices. Check ALL that apply.]

STDS6MO STDs in past 6 months
2

0 - 8 = range
97 = Don't Know
98 = Refuse to Answer
99 = Not Applicable

STDS6MOA STDs in past 6 months: Syphilis
1

0 = No
1 = Yes
7 = Don't Know
8 = Refuse to Answer
9 = Not Applicable

STDS6MOB STDs in past 6 months: Gonorrhea
1

0 = No
1 = Yes
7 = Don't Know
8 = Refuse to Answer
9 = Not Applicable

STDS6MOC STDs in past 6 months: Chlamydia
1

0 = No
1 = Yes
7 = Don't Know
8 = Refuse to Answer
9 = Not Applicable

STDS6MOD STDs in past 6 months: Herpes
1

0 = No
1 = Yes
7 = Don't Know
8 = Refuse to Answer
9 = Not Applicable

STDS6MOE STDs in past 6 months: HPV (Genital Warts)
1

0 = No
1 = Yes
7 = Don't Know
8 = Refuse to Answer
9 = Not Applicable

STDS6MOF STDs in past 6 months: Other
1

0 = No
1 = Yes
7 = Don't Know
8 = Refuse to Answer
9 = Not Applicable

STDS6MOG STDs in past 6 months: None
1

0 = No
1 = Yes
7 = Don't Know
8 = Refuse to Answer
9 = Not Applicable

STDS6MOH STDs in past 6 months: Have not tested in past 6 months
1

0 = No
1 = Yes
7 = Don't Know
8 = Refuse to Answer
9 = Not Applicable

Q561. Please specify what other STDs you have been told you had in the past 6 months

STDSOTH Other STDs specify
50

0 - 50 = range
97 = Don't Know
98 = Refuse to Answer
99 = Not Applicable

Q562. Have you ever been tested for Hepatitis C?

HCVTEST Ever tested for Hepatitis C
1

0 = No
1 = Yes
7 = Don't Know
8 = Refuse to Answer
9 = Not Applicable

Q563. What was the result of your Hepatitis C test?

HCVRES HCV test result
1

0 = Negative
1 = Positive
7 = Don't Know
8 = Refuse to Answer
9 = Not Applicable

Q564. Did you have a Hepatitis C viral load test?

HCVVL HCV viral load test
1

0 = No
1 = Yes
7 = Don't Know
8 = Refuse to Answer
9 = Not Applicable

Q565. What was the result of your Hepatitis C viral load test?

HCVVLRES HCV viral load test result 1

0 = Negative
1 = Positive
7 = Don't Know
8 = Refuse to Answer
9 = Not Applicable

Q566. Have you ever received treatment for your Hepatitis C?

HCVTX Ever received HCV treatment 1

0 = No
1 = Yes
7 = Don't Know
8 = Refuse to Answer
9 = Not Applicable

Q567. Have you ever been told by a doctor or nurse that you cleared the Hepatitis C virus?

HCVCLR Cleared HCV virus 1

0 = No
1 = Yes
7 = Don't Know
8 = Refuse to Answer
9 = Not Applicable

Q568. Have you ever experienced any of the following health conditions? [READ choices. Check ALL that apply.]

HLTHCON Health conditions 2

0 - 13 = range
97 = Don't Know
98 = Refuse to Answer
99 = Not Applicable

HLTHCONA Health conditions: Thromboembolism 1

0 = No
1 = Yes
7 = Don't Know
8 = Refuse to Answer
9 = Not Applicable

HLTHCONB Health conditions: Liver dysfunction 1

0 = No
1 = Yes
7 = Don't Know
8 = Refuse to Answer
9 = Not Applicable

HLTHCONC Health conditions: Cardiovascular disease

1

0 = No
1 = Yes
7 = Don't Know
8 = Refuse to Answer
9 = Not Applicable

HLTHCOND Health conditions: Cancer
1

0 = No
1 = Yes
7 = Don't Know
8 = Refuse to Answer
9 = Not Applicable

HLTHCONE Health conditions: Mood swings
1

0 = No
1 = Yes
7 = Don't Know
8 = Refuse to Answer
9 = Not Applicable

HLTHCONF Health conditions: Weight changes
1

0 = No
1 = Yes
7 = Don't Know
8 = Refuse to Answer
9 = Not Applicable

HLTHCONG Health conditions: Fibroadenoma (cysts)
1

0 = No
1 = Yes
7 = Don't Know
8 = Refuse to Answer
9 = Not Applicable

HLTHCONH Health conditions: Type II Diabetes
1

0 = No
1 = Yes
7 = Don't Know
8 = Refuse to Answer
9 = Not Applicable

HLTHCONI Health conditions: Osteoporosis
1

0 = No
1 = Yes
7 = Don't Know
8 = Refuse to Answer
9 = Not Applicable

HLTHCONJ Health conditions: Lupus
1

0 = No
1 = Yes
7 = Don't Know
8 = Refuse to Answer
9 = Not Applicable

HLTHCONK Health conditions: Hepatitis B
1

0 = No
1 = Yes
7 = Don't Know
8 = Refuse to Answer
9 = Not Applicable

HLTHCONL Health conditions: Hepatitis C
1

0 = No
1 = Yes
7 = Don't Know
8 = Refuse to Answer
9 = Not Applicable

HLTHCONM Health conditions: None of the above
1

0 = No
1 = Yes
7 = Don't Know
8 = Refuse to Answer
9 = Not Applicable

Q569. Was your thromboembolism related to hormone use?

HORMCOMA Hormones complications thromboembolism
1

0 = No
1 = Yes
7 = Don't Know
8 = Refuse to Answer
9 = Not Applicable

Q570. Was your liver dysfunction related to hormone use?

HORMCOMB Hormones complications liver dysfunction
1

0 = No
1 = Yes
7 = Don't Know
8 = Refuse to Answer
9 = Not Applicable

Q571. Was your cardiovascular disease related to hormone use?

HORMCOMC Hormones complications cardiovascular disease
1

0 = No
1 = Yes
7 = Don't Know
8 = Refuse to Answer
9 = Not Applicable

Q572. Was your cancer related to hormone use?

HORMCOMD Hormones complications cancer
1

0 = No
1 = Yes
7 = Don't Know
8 = Refuse to Answer
9 = Not Applicable

Q573. Were your mood swings related to hormone use?

HORMCOME Hormones complications mood swings
1

0 = No
1 = Yes
7 = Don't Know
8 = Refuse to Answer
9 = Not Applicable

Q574. Were your weight changes related to hormone use?

HORMCOMF Hormones complications weight changes
1

0 = No
1 = Yes
7 = Don't Know
8 = Refuse to Answer
9 = Not Applicable

Q575. Was your fibroadenoma (cysts) related to hormone use?

HORMCOMG Hormones complications fibroadenoma
1

0 = No
1 = Yes
7 = Don't Know
8 = Refuse to Answer
9 = Not Applicable

Q576. Was your Type II diabetes related to hormone use?

HORMCOMH Hormones complications diabetes
1

0 = No
1 = Yes
7 = Don't Know
8 = Refuse to Answer
9 = Not Applicable

Q577. Was your osteoporosis related to hormone use?

HORMCOMI Hormones complications osteoporosis
1

0 = No
1 = Yes
7 = Don't Know
8 = Refuse to Answer
9 = Not Applicable

Q578. Was your lupus related to hormone use?

HORMCOMJ Hormones complications lupus
1

0 = No
1 = Yes
7 = Don't Know
8 = Refuse to Answer
9 = Not Applicable

Q579. Have you heard of PrEP before today?

PREPHRD Heard of PrEP to prevent HIV
1

0 = No
1 = Yes
7 = Don't Know
8 = Refuse to Answer
9 = Not Applicable

Q580. In the past 12 months, have you taken PrEP? (This is different from PEP, which you can take <u>after</u> sex to prevent HIV.)

PREP12M Taken PrEP before sex to prevent HIV
1

0 = No
1 = Yes
7 = Don't Know
8 = Refuse to Answer
9 = Not Applicable

Q581. How interested would you be in taking PrEP?

PREPINT Interest in PrEP
1

0 = Not at all interested
1 = Not interested
2 = Neutral
3 = Interested
4 = Very interested
7 = Don't Know
8 = Refuse to Answer
9 = Not Applicable

Q582. Next I'm going to read a list of reasons some people may not take PrEP. Please let me know if any of them apply to you.

WHYNOPR Why no PrEP
2

0 - 15 = range
97 = Don't Know
98 = Refuse to Answer
99 = Not Applicable

WHYNOPRA Why no PrEP: No insurance/cost
1

0 = No
1 = Yes
7 = Don't Know
8 = Refuse to Answer
9 = Not Applicable

WHYNOPRB Why no PrEP: Don't know where to go for it
1

0 = No
1 = Yes
7 = Don't Know
8 = Refuse to Answer
9 = Not Applicable

WHYNOPRC Why no PrEP: Don't feel it's necessary for me
1

0 = No
1 = Yes
7 = Don't Know
8 = Refuse to Answer
9 = Not Applicable

WHYNOPRD Why no PrEP: Happy using other methods of HIV prevention
1

0 = No
1 = Yes
7 = Don't Know
8 = Refuse to Answer
9 = Not Applicable

WHYNOPRE Why no PrEP: Not concerned if I get HIV
1

0 = No
1 = Yes
7 = Don't Know
8 = Refuse to Answer
9 = Not Applicable

WHYNOPRF Why no PrEP: Worried about side effects
1

0 = No
1 = Yes
7 = Don't Know
8 = Refuse to Answer
9 = Not Applicable

WHYNOPRG Why no PrEP: Worried about stigma about sexual activities 1

0 = No
1 = Yes
7 = Don't Know
8 = Refuse to Answer
9 = Not Applicable

WHYNOPRH Why no PrEP: Worried about stigma if seen taking an HIV med 1

0 = No
1 = Yes
7 = Don't Know
8 = Refuse to Answer
9 = Not Applicable

WHYNOPRI Why no PrEP: Don't want to take another pill 1

0 = No
1 = Yes
7 = Don't Know
8 = Refuse to Answer
9 = Not Applicable

WHYNOPRJ Why no PrEP: Wasn't aware of it 1

0 = No
1 = Yes
7 = Don't Know
8 = Refuse to Answer
9 = Not Applicable

WHYNOPRK Why no PrEP: Didn't ask for it 1

0 = No
1 = Yes
7 = Don't Know
8 = Refuse to Answer
9 = Not Applicable

WHYNOPRL Why no PrEP: Wasn't offered it 1

0 = No
1 = Yes
7 = Don't Know
8 = Refuse to Answer
9 = Not Applicable

WHYNOPRM Why no PrEP: Worried about interactions with hormones 1

0 = No
1 = Yes
7 = Don't Know
8 = Refuse to Answer

9 = Not Applicable

WHYNOPRN Why no PrEP: Worried about interactions with other meds
1

0 = No
1 = Yes
7 = Don't Know
8 = Refuse to Answer
9 = Not Applicable

WHYNOPRO Why no PrEP: Other
1

0 = No
1 = Yes
7 = Don't Know
8 = Refuse to Answer
9 = Not Applicable

Q583. Please specify other reasons for not taking PrEP.

NOPRSPEC Specify other reasons for no PrEP
50

0 - 50 = range
97 = Don't Know
98 = Refuse to Answer
99 = Not Applicable

Q584. What kind of health insurance or coverage do you currently have? [Do NOT read choices. Check ALL that apply.]

INSURTY Type of health insurance
2

0 - 8 = range
97 = Don't Know
98 = Refuse to Answer
99 = Not Applicable

INSURTYA Type of health insurance: No current health insurance
1

0 = No
1 = Yes
7 = Don't Know
8 = Refuse to Answer
9 = Not Applicable

INSURTYB Type of health insurance: Private insurance or HMO
1

0 = No
1 = Yes
7 = Don't Know
8 = Refuse to Answer
9 = Not Applicable

INSURTYC Type of health insurance: Medi-Cal/Medicaid
1

0 = No
1 = Yes
7 = Don't Know
8 = Refuse to Answer
9 = Not Applicable

INSURTYD Type of health insurance: Medicare
1

0 = No
1 = Yes
7 = Don't Know
8 = Refuse to Answer
9 = Not Applicable

INSURTYE Type of health insurance: Veterans Administration Coverage
1

0 = No
1 = Yes
7 = Don't Know
8 = Refuse to Answer
9 = Not Applicable

INSURTYF Type of health insurance: Tricare (Champus)
1

0 = No
1 = Yes
7 = Don't Know
8 = Refuse to Answer
9 = Not Applicable

INSURTYG Type of health insurance: Healthy San Francisco
1

0 = No
1 = Yes
7 = Don't Know
8 = Refuse to Answer
9 = Not Applicable

INSURTYH Type of health insurance: Other
1

0 = No
1 = Yes
7 = Don't Know
8 = Refuse to Answer
9 = Not Applicable

Q585. Please specify your current type of health insurance

SPECINS Specify health insurance
50

0 - 50 = range
97 = Don't Know
98 = Refuse to Answer
99 = Not Applicable

Q586. Have you seen a doctor, nurse, or other health care provider in the past 12 months?

HCPST12M Health care provider in past 12 months 1

- **0** = No, but I didn't need to
- **1** = No, and I needed to
- **2** = Yes
- **7** = Don't Know
- **8** = Refuse to Answer
- **9** = Not Applicable

Q587. In the past year, have you had any problems getting <u>medical health services</u> because of your gender identity or gender presentation?

HCPROBS Problems getting health care 1

- **0** = No, but I did not seek these services
- **1** = No, and I sought these services
- **2** = Yes
- **7** = Don't Know
- **8** = Refuse to Answer
- **9** = Not Applicable

Q588. Do the health care services you have received meet your trans-specific medical health care needs?

HCNEEDS Health care meets trans specific needs 1

- **0** = No, but I did not need/access them
- **1** = No, and I needed/accessed them
- **2** = Yes
- **7** = Don't Know
- **8** = Refuse to Answer
- **9** = Not Applicable

Q589. There are different types of mental health issues people deal with. Have you been diagnosed with any of the following mental health issues?

MHLTTYP Types of mental health issues 1

- **0 - 4** = range
- **7** = Don't Know
- **8** = Refuse to Answer
- **9** = Not Applicable

MHLTTYPA Types of mental health issues: Depression 1

- **0** = No
- **1** = Yes
- **7** = Don't Know
- **8** = Refuse to Answer
- **9** = Not Applicable

MHLTTYPB Types of mental health issues: PTSD (Post-traumatic stress disorder) 1

- **0** = No
- **1** = Yes

7 = Don't Know
8 = Refuse to Answer
9 = Not Applicable

MHLTTYPC Types of mental health issues: Anxiety
1

0 = No
1 = Yes
7 = Don't Know
8 = Refuse to Answer
9 = Not Applicable

MHLTTYPD Types of mental health issues: None of the above
1

0 = No
1 = Yes
7 = Don't Know
8 = Refuse to Answer
9 = Not Applicable

Q590. Have you been seen by a professional for mental health services in the past 12 months?

MENTHLT Mental health care provider in past 12 months
1

0 = No, but I didn't need to
1 = No, and I needed to
2 = Yes
7 = Don't Know
8 = Refuse to Answer
9 = Not Applicable

Q591. In the past year, have you had any problems getting mental health services because of your gender identity or gender presentation?

MHCPROBS Problems getting mental health care
1

0 = No, but I didn't seek these services
1 = No, and I sought these services
2 = Yes
7 = Don't Know
8 = Refuse to Answer
9 = Not Applicable

Q592. Do/did the mental health services you have received meet your trans-specific mental health care needs?

MHCNEED Mental health care meets trans needs
1

0 = No, but I didn't need/access them
1 = No, and I needed/accessed them
2 = Yes
7 = Don't Know
8 = Refuse to Answer
9 = Not Applicable

Q593. Did you use any of the following services in 2015? [READ choices. Check ALL that apply.]

SRVCS Service utilization in 2015 2

0 - 7 = range
97 = Don't Know
98 = Refuse to Answer
99 = Not Applicable

SRVCSA Service utilization in 2015: TransThrive/API Wellness Center 1

0 = No
1 = Yes
7 = Don't Know
8 = Refuse to Answer
9 = Not Applicable

SRVCSB Service utilization in 2015: Transgender Surgery Access Program at SFDPH Transgender Health Services 1

0 = No
1 = Yes
7 = Don't Know
8 = Refuse to Answer
9 = Not Applicable

SRVCSC Service utilization in 2015: LGBT Center/Transgender Employment Services 1

0 = No
1 = Yes
7 = Don't Know
8 = Refuse to Answer
9 = Not Applicable

SRVCSD Service utilization in 2015: Lyon Martin Health Services 1

0 = No
1 = Yes
7 = Don't Know
8 = Refuse to Answer
9 = Not Applicable

SRVCSE Service utilization in 2015: Tri-City Health Center 1

0 = No
1 = Yes
7 = Don't Know
8 = Refuse to Answer
9 = Not Applicable

SRVCSF Service utilization in 2015: None 1

0 = No
1 = Yes
7 = Don't Know
8 = Refuse to Answer

9 = Not Applicable

SRVCSG Service utilization in 2015: Other
1

0 = No
1 = Yes
7 = Don't Know
8 = Refuse to Answer
9 = Not Applicable

Q594. Which services at TransThrive/API Wellness Center did you access in 2015? [READ choices. Check ALL that apply.]

APIWC Services accessed at API Wellness
2

0 - 13 = range
97 = Don't Know
98 = Refuse to Answer
99 = Not Applicable

APIWCA Services accessed at API Wellness: Primary care
1

0 = No
1 = Yes
7 = Don't Know
8 = Refuse to Answer
9 = Not Applicable

APIWCB Services accessed at API Wellness: Hormones
1

0 = No
1 = Yes
7 = Don't Know
8 = Refuse to Answer
9 = Not Applicable

APIWCC Services accessed at API Wellness: Family planning
1

0 = No
1 = Yes
7 = Don't Know
8 = Refuse to Answer
9 = Not Applicable

APIWCD Services accessed at API Wellness: Mental health
1

0 = No
1 = Yes
7 = Don't Know
8 = Refuse to Answer
9 = Not Applicable

APIWCE Services accessed at API Wellness: Substance use counseling
1

0 = No

1 = Yes
7 = Don't Know
8 = Refuse to Answer
9 = Not Applicable

APIWCF Services accessed at API Wellness: HIV testing
1

0 = No
1 = Yes
7 = Don't Know
8 = Refuse to Answer
9 = Not Applicable

APIWCG Services accessed at API Wellness: Health education workshop
1

0 = No
1 = Yes
7 = Don't Know
8 = Refuse to Answer
9 = Not Applicable

APIWCH Services accessed at API Wellness: Support group
1

0 = No
1 = Yes
7 = Don't Know
8 = Refuse to Answer
9 = Not Applicable

APIWCI Services accessed at API Wellness: HIV treatment
1

0 = No
1 = Yes
7 = Don't Know
8 = Refuse to Answer
9 = Not Applicable

APIWCJ Services accessed at API Wellness: Case management/peer navigation
1

0 = No
1 = Yes
7 = Don't Know
8 = Refuse to Answer
9 = Not Applicable

APIWCK Services accessed at API Wellness: Trans:Thrive drop-in
1

0 = No
1 = Yes
7 = Don't Know
8 = Refuse to Answer
9 = Not Applicable

APIWCL Services accessed at API Wellness: Needle exchange

1

0 = No
1 = Yes
7 = Don't Know
8 = Refuse to Answer
9 = Not Applicable

APIWCM Services accessed at API Wellness: Other
1

0 = No
1 = Yes
7 = Don't Know
8 = Refuse to Answer
9 = Not Applicable

Q595. How many transwomen would you say live in San Francisco?

WOCSF Wisdom of the crowd San Francisco
7

0 - 999999 = range
9999997 = Don't Know
9999998 = Refuse to Answer
9999999 = Not Applicable

Q596. How many transwomen would you say live in the Greater Bay Area (<u>NOT</u> including San Francisco)?

WOCBAY Wisdom of the crowd Greater Bay Area
7

0 - 999999 = range
9999997 = Don't Know
9999998 = Refuse to Answer
9999999 = Not Applicable

Q597. How would you rate your quality of life?

QOL Quality of life
1

1 = Very poor
2 = Poor
3 = Neither poor nor good
4 = Good
5 = Very good
7 = Don't Know
8 = Refuse to Answer
9 = Not Applicable

Q598. If you could change one thing for transwomen in the Bay Area, what would it be?

CHANGE Change one thing for transwomen
200

0 - 200 = range
997 = Don't Know
998 = Refuse to Answer
999 = Not Applicable

yy1. **You did not consent to HIV testing in the beginning of this interview. Just to be sure, do you want to consent to an HIV test?**

CONTEST2 Consent for HIV test revisit
1

0 = No
1 = Yes
7 = Don't Know
8 = Refuse to Answer
9 = Not Applicable

yy2. **You did not consent to Dried Blood Spot storage in the beginning of this interview. Just to be sure, do you want to consent to Dried Blood Spot storage?**

CONDBS2 Consent for DBS revisit
1

0 = No
1 = Yes
7 = Don't Know
8 = Refuse to Answer
9 = Not Applicable

yy3. **You did not consent to Hepatitis C testing in the beginning of this interview. Just to be sure, do you want to consent to a Hepatitis C test?**

CONHCV2 Consent for HCV test revisit
1

0 = No
1 = Yes
7 = Don't Know
8 = Refuse to Answer
9 = Not Applicable

yy4. **INTERVIEWER: How confident are you of the validity of the respondent's answers?**

VALIDITY Confidence in validity of answers
1

1 = Confident
2 = Some doubts
3 = Not confident at all
7 = Don't Know
8 = Refuse to Answer
9 = Not Applicable

yy5. **Interviewer: Please explain why you are not confident in the respondent's answers.**

VALSPEC Specify why not confident in answers
200

0 - 200 = range
997 = Don't Know
998 = Refuse to Answer
999 = Not Applicable

yy6. **Interviewer: Do you have any comments to add?**

INTCOMM Interviewer comments
1

0 = No

1 = Yes
7 = Don't Know
8 = Refuse to Answer
9 = Not Applicable

yy7. Interviewer: Enter comments

INTTXT — Interviewer comments text
1000

0 - 1000 = range
9997 = Don't Know
9998 = Refuse to Answer
9999 = Not Applicable

yy8. Do you want to end this survey?

ENDSURV — End survey
1

0 = No
1 = Yes
7 = Don't Know
8 = Refuse to Answer
9 = Not Applicable

Calculated Variable

END — Interview end time

END = Current time

Calculated Variable

INTTIME

INTTIME = END-START
