# Supplementary material for: Prevalence and correlates of substance use and associations with HIV-related outcomes among trans women in the San Francisco Bay Area
Source: BMC Infect Dis. 2022 Nov 26;22:886. doi: 10.1186/s12879-022-07868-4 (PMC9701418; doi:10.1186/s12879-022-07868-4)
Supplement: Supplementary file 2 — Additional file 2: Table 1. Bivariates. Selected descriptive bivariates for sociodemographic and health-related characteristics. [file 12879_2022_7868_MOESM2_ESM.docx]

**Supplemental Table 1. Descriptive bivariates for selected characteristics among trans women, San Francisco, 2016-2017 (N=629)**

| **Sociodemographic or Health-related Characteristic** | **Any substance** | **Marijuana** | **Methamphetamine** | **Crack/cocaine** | **Any substance with sex** | **Marijuana with sex** | **Methamphetamine with sex** | **Crack/cocaine with sex** | **Injection drug use** | **Polysubstance use** |
| --- | --- | --- | --- | --- | --- | --- | --- | --- | --- | --- |
|  | *Odds Ratio^a^ (95% CI)* | | | | | | | | | |
| **Age group in years** |  |  |  |  |  |  |  |  |  |  |
| 18-24 (ref) |  |  |  |  |  |  |  |  |  |  |
| 25-34 | 0.792 (0.434 - 1.445) | 0.845 (0.482 - 1.482) | 0.953 (0.489 - 1.857) | 0.895 (0.476 - 1.683) | 0.657* (0.375 - 1.151) | 0.578* (0.327 - 1.022) | 1.304 (0.600 - 2.837) | 0.813 (0.346 - 1.910) | 0.895 (0.326 - 2.457) | 0.780 (0.446 - 1.364) |
| 35-44 | 0.441** (0.240 - 0.808) | 0.399** (0.222 - 0.716) | 1.635* (0.847 - 3.157) | 0.433** (0.213 - 0.878) | 0.506** (0.284 - 0.902) | 0.299** (0.161 - 0.558) | 1.855* (0.857 - 4.014) | 0.561* (0.221 - 1.423) | 1.067 (0.388 - 2.936) | 0.487** (0.272 - 0.872) |
| 45-54 | 0.345** (0.187 - 0.636) | 0.224** (0.121 - 0.415) | 1.270 (0.649 - 2.484) | 0.505* (0.252 - 1.013) | 0.433** (0.241 - 0.778) | 0.188** (0.0958 - 0.369) | 1.868* (0.860 - 4.055) | 0.759 (0.311 - 1.851) | 1.486 (0.559 - 3.955) | 0.368** (0.203 - 0.669) |
| 55-64 | 0.253** (0.129 - 0.493) | 0.232** (0.117 - 0.458) | 0.684 (0.311 - 1.503) | 0.340** (0.146 - 0.789) | 0.310** (0.160 - 0.601) | 0.280** (0.138 - 0.569) | 0.935 (0.378 - 2.310) | 0.579 (0.204 - 1.640) | 1.200 (0.406 - 3.548) | 0.207** (0.101 - 0.423) |
| 65+ | 0.250** (0.0863 - 0.724) | 0.346* (0.118 - 1.016) | - | 0.149* (0.0186 - 1.195) | 0.212** (0.0639 - 0.703) | 0.223** (0.0595 - 0.833) | - | 0.377 (0.0447 - 3.174) | - | 0.177** (0.0473 - 0.662) |
| **Race/ethnicity** |  |  |  |  |  |  |  |  |  |  |
| White (ref) |  |  |  |  |  |  |  |  |  |  |
| Asian/Asian American | 0.896 (0.354 - 2.269) | 0.372* (0.130 - 1.067) | 2.184* (0.813 - 5.869) | 0.206* (0.0267 - 1.591) | 1.067 (0.415 - 2.739) | 0.466* (0.131 - 1.659) | 2.821* (0.989 - 8.049) | 0.546 (0.0685 - 4.350) | - | 0.521* (0.181 - 1.497) |
| Black/African American | 1.010 (0.623 - 1.639) | 0.780 (0.481 - 1.263) | 1.889** (1.094 - 3.262) | 1.133 (0.634 - 2.024) | 1.692** (1.044 - 2.742) | 1.177 (0.697 - 1.988) | 2.685** (1.475 - 4.888) | 1.960* (0.945 - 4.064) | 1.120 (0.533 - 2.355) | 1.010 (0.619 - 1.648) |
| Native American | 0.244* (0.0249 - 2.395) | 0.372 (0.0380 - 3.644) | - | - | - | - | - | - | - | - |
| Native Hawaiian/Pacific Islander | 0.587 (0.153 - 2.257) | 0.558 (0.135 - 2.300) | 2.028 (0.484 - 8.500) | 0.49 (0.0594 - 4.040) | 1.280 (0.332 - 4.929) | 0.754 (0.152 - 3.754) | 3.292* (0.771 - 14.05) | 1.297 (0.152 - 11.04) | 2.314 (0.450 - 11.91) | 0.782 (0.189 - 3.226) |
| Other or Multiple | 0.825 (0.507 - 1.343) | 0.583** (0.353 - 0.963) | 1.460* (0.825 - 2.585) | 1.141 (0.634 - 2.055) | 0.873 (0.527 - 1.445) | 0.726 (0.409 - 1.288) | 1.606* (0.838 - 3.078) | 1.383 (0.627 - 3.052) | 0.979 (0.449 - 2.134) | 0.890 (0.539 - 1.470) |
| Hispanic/Latinx | 0.646** (0.432 - 0.966) | 0.507** (0.335 - 0.767) | 1.173 (0.718 - 1.916) | 0.620* (0.362 - 1.061) | 0.943 (0.625 - 1.423) | 0.743* (0.467 - 1.181) | 1.355 (0.772 - 2.379) | 0.702 (0.328 - 1.503) | 0.504* (0.239 - 1.061) | 0.662* (0.434 - 1.010) |
| **At or below extremely low-income limit for San Francisco** | |  |  |  |  |  |  |  |  |  |
| No (ref) |  |  |  |  |  |  |  |  |  |  |
| Yes | 0.822 (0.559 - 1.208) | 0.663** (0.450 - 0.977) | 2.070** (1.236 - 3.467) | 0.959 (0.586 - 1.569) | 0.790* (0.536 - 1.163) | 0.507** (0.335 - 0.769) | 1.925** (1.093 - 3.391) | 0.806 (0.434 - 1.496) | 2.470** (1.036 - 5.888) | 1.067 (0.712 - 1.598) |
| **Ever experienced adult housing instability** | |  |  |  |  |  |  |  |  |  |
| No (ref) |  |  |  |  |  |  |  |  |  |  |
| Yes | 1.449** (1.020 - 2.059) | 1.339* (0.927 - 1.936) | 4.214** (2.427 - 7.318) | 1.735** (1.050 - 2.867) | 1.589** (1.097 - 2.300) | 1.283* (0.844 - 1.952) | 4.113** (2.201 - 7.687) | 2.295** (1.104 - 4.769) | 7.792** (2.404 - 25.25) | 2.061** (1.385 - 3.068) |
| **Ever sex work** |  |  |  |  |  |  |  |  |  |  |
| No (ref) |  |  |  |  |  |  |  |  |  |  |
| Yes | 1.879** (1.349 - 2.618) | 1.476** (1.046 - 2.084) | 6.525** (3.769 - 11.30) | 2.201** (1.365 - 3.550) | 2.415** (1.692 - 3.448) | 1.714** (1.146 - 2.563) | 10.02** (4.786 - 20.97) | 2.335** (1.213 - 4.496) | 6.456** (2.541 - 16.40) | 2.439** (1.681 - 3.537) |
| **HIV status** |  |  |  |  |  |  |  |  |  |  |
| Negative (ref) |  |  |  |  |  |  |  |  |  |  |
| Positive | 1.073 (0.760 - 1.515) | 0.720* (0.502 - 1.035) | 2.042** (1.393 - 2.994) | 1.029 (0.660 - 1.603) | 1.208 (0.852 - 1.713) | 0.827 (0.551 - 1.244) | 2.211** (1.463 - 3.341) | 1.696* (0.980 - 2.933) | 1.410* (0.801 - 2.482) | 0.958 (0.667 - 1.375) |
| **Health insurance status** |  |  |  |  |  |  |  |  |  |  |
| Insured (ref) |  |  |  |  |  |  |  |  |  |  |
| Uninsured | 2.930** (1.301 - 6.602) | 2.356** (1.158 - 4.794) | 2.736** (1.343 - 5.571) | 1.017 (0.410 - 2.524) | 3.741** (1.748 - 8.006) | 2.374** (1.160 - 4.857) | 2.585** (1.234 - 5.415) | 1.740 (0.646 - 4.689) | 2.299* (0.908 - 5.823) | 2.364** (1.167 - 4.789) |
| **Unmet gender-affirming health care needs** | |  |  |  |  |  |  |  |  |  |
| No (ref) |  |  |  |  |  |  |  |  |  |  |
| Yes | 1.856** (1.252 - 2.751) | 1.760** (1.199 - 2.583) | 0.946 (0.605 - 1.478) | 1.301 (0.811 - 2.086) | 1.537** (1.048 - 2.254) | 1.722** (1.137 - 2.608) | 1.017 (0.627 - 1.648) | 1.638* (0.910 - 2.950) | 1.061 (0.554 - 2.030) | 1.724** (1.171 - 2.540) |
| **Ever experienced gender-based discrimination** | |  |  |  |  |  |  |  |  |  |
| No (ref) |  |  |  |  |  |  |  |  |  |  |
| Yes | 1.989** (1.049 - 3.769) | 1.622* (0.816 - 3.223) | 1.706* (0.743 - 3.917) | 1.737 (0.668 - 4.515) | 2.273** (1.099 - 4.700) | 2.615** (1.011 - 6.765) | 3.291** (1.000 - 10.82) | 1.437 (0.431 - 4.792) | 2.170 (0.511 - 9.213) | 1.829* (0.884 - 3.786) |
| **Ever experienced race-based violence** | |  |  |  |  |  |  |  |  |  |
| No (ref) |  |  |  |  |  |  |  |  |  |  |
| Yes | 1.127 (0.816 - 1.555) | 0.967 (0.694 - 1.347) | 1.615** (1.117 - 2.335) | 0.970 (0.638 - 1.473) | 1.203 (0.867 - 1.670) | 0.951 (0.654 - 1.382) | 1.848** (1.236 - 2.765) | 1.827** (1.071 - 3.118) | 2.142** (1.242 - 3.694) | 0.917 (0.654 - 1.287) |
| **Condomless anal sex in the past 6 months** | |  |  |  |  |  |  |  |  |  |
| No (ref) |  |  |  |  |  |  |  |  |  |  |
| Yes | 2.104** (1.501 - 2.948) | 1.714** (1.226 - 2.397) | 2.604** (1.793 - 3.782) | 1.875** (1.243 - 2.830) | 2.982** (2.124 - 4.188) | 2.519** (1.737 - 3.653) | 2.852** (1.897 - 4.288) | 2.298** (1.344 - 3.929) | 2.501** (1.448 - 4.318) | 2.406** (1.710 - 3.386) |

^a^p-values for odds ratios are indicated as follows: *p<0.25, **p<0.05
